# Beaver in tidal habitat: Examples from the Pacific Northwest

**DOI:** 10.1371/journal.pone.0349313

**Published:** 2026-07-08

**Authors:** W. Gregory Hood

**Affiliations:** Skagit River System Cooperative, Burlington, Washington, United States of America; Universite Libre de Bruxelles, BELGIUM

## Abstract

Beaver are typically considered fluvial or lacustrine animals, often converting lotic habitats into lentic ones with their dams. This results in extensive changes in ecosystem structure and processes so that beaver are considered the quintessential ecosystem engineer. Here I broaden our appreciation of the adaptability of beaver by describing their widespread presence in tidal river deltas and estuaries of the Pacific Northwest (coastal British Columbia, Washington, and Oregon), where tides can range between 1.5 and 5.0 m. These observations expand the known habitat distribution of beaver and invite investigation of the ecosystem consequences of beaver in tidal wetlands. In these oligohaline to fresh tidal systems, channel profile surveys with real-time kinematic (RTK)-GPS show that beaver dams are typically flooded on higher high tides, only impounding water at low tide to allow beaver movement during this time. Tidal beaver dam density per km was more than twice that reported in the fluvial literature, while mean dam head was about 80% and mean dam height about 60% of fluvial dams. Low-tide beaver pool depths were 75% of fluvial beaver ponds, while pool areas were 67% of their fluvial counterparts. The comparable beaver dam metrics between fluvial and tidal systems suggests their role in tidal ecosystems may be comparably significant. Inspection of Google Earth aerial photographs back to 1990 indicated that tidal beaver dams can persist for at least 35 years, spanning several generations of beaver. Given the ecosystem importance of beaver in fluvial and lacustrine habitats, better understanding of the distribution and ecosystem role of beaver in tidal wetland habitat, and the geometry of their dams and low-tide pools, would likely allow more effective restoration of estuarine habitat that is critical to a variety of fish and wildlife, including threatened species such as Chinook and coho salmon.

## Introduction

Beaver (*Castor canadensis* [North America] and *C. fiber* [Eurasia]) are iconic and charismatic animals, so known for their industrious construction of dams in streams that they have inspired the phrase, “busy as a beaver”. A result of their dam construction and excavation of small channels in floodplains is thorough alteration of their environment to benefit wetland and riparian vegetation which provide food for the beaver. Fish, birds, and other wildlife also benefit from the habitat changes that result from increased ponding along otherwise lotic systems [1–4]. Beaver dams and their ponds can profoundly alter fluvial hydrology, geomorphology, and ecosystem function [5]. For this reason, beaver are the archetype ecosystem engineer [6,7].

While the importance of beaver to fluvial ecosystems has long been known, the presence of beaver in tidal systems has been largely overlooked. In the Skagit Delta (Puget Sound, Washington, USA), where twice-daily tides can vary by four meters, intertidal beaver dams and lodges are common, but little is known about how beaver in tidal systems compare to those in more “traditional” fluvial systems. Yet there are indications that their influences on tidal ecosystems could be as significant as those on fluvial ecosystem. For example, recent work has shown that Skagit Delta beaver dams quadruple tidal channel (low-tide) pool habitat for fish, compared to tidal channels without beaver [8]. The Skagit beaver pools also contain triple the density (by volume) of threatened juvenile Chinook salmon (*Oncorhynchus tshawytscha*) compared to low-tide channel shallows. At low tide, channels without pools often dewater almost completely, forcing most fish to emigrate to larger, deeper channels with subtidal habitat. Channels with low-tide pools may allow (1) longer residence of small fish in productive tidal marsh channel habitat, (2) lower energy expenditure because they fish are not forced to move to other areas, (3) perhaps lower predation pressure because they are not forced into deeper waters on the ebb tide, and (4) perhaps greater food resources because low-tide beaver ponds appear to trap a lot of organic detritus [8], though these hypothesized benefits to fish have yet to be proven.

Tidal estuarine ecosystems have generally been considered an unexpected place to find beaver. The scientific literature on fluvial beaver is extensive, while for tidal beaver it is nearly non-existent. There are significant differences between tidal and fluvial systems that beaver must confront. The most prominent are bidirectional flow (flood vs. ebb tides) vs. unidirectional flow; typically twice daily water level fluctuations in systems that are micro-tidal (< 2 m fluctuations), meso-tidal (2–4 m), macro-tidal (4–6), or hyper-tidal (6–15 m), where low tides in most systems can drain many channels entirely, vs. seasonal and episodic floods or droughts (which can also occur in tidal systems fed by rivers); and often the presence of a range of salinities ranging from freshwater (< 0.5 ppt) to seawater (35 ppt) to hypersaline in arid areas, which contrasts with solely freshwater in fluvial systems. However, salinity stratification (freshwater floating on heavier saltwater) is common, so some estuarine plants and animals can reduce their exposure to salt stress. Tidal marshes tend to have significantly higher drainage density than uplands; their networks are frequently interconnected by loops so that they are reticulated rather than dendritic like fluvial networks; and tidal marshes are very flat so that drainage divides are often hard to define, while fluvial divides are characterized by significant topographic relief [9]. Altogether, these network differences make tidal systems very two-dimensional while fluvial systems are comparatively one-dimensional.

Because so little is known about beaver in tidal wetlands, knowledge gaps are extensive. Thus, the goal of this paper is to provide fundamental natural history information on intertidal beaver dams and to highlight the need for further investigation of beaver in tidal habitats. This paper is structured by two foci of investigation. The first focus is an exploratory analysis of local-scale patterns of intertidal beaver dam and lodge distributions in tidal wetlands of the Skagit and Snohomish deltas, the two largest rivers draining into Puget Sound (Washington, USA). The goal of this focus is to explore spatial patterns in dam and lodge locations (a) to provide insight into the abundance of beaver structures and their distribution relative to estuarine habitats, e.g., characterized by vegetation communities and channel networks; (b) to allow comparison with fluvial beaver, e.g., are there significant differences in dam or lodge density that might suggest differences in habitat quality or beaver behavior; (c) to understand the function of dams for beaver in tidal environments and how that compares with fluvial dams; and (d) to contribute guidance for tidal wetland restoration design that accommodates beaver and the ecosystem functions they may provide. The second focus is an assessment of the region-scale distribution of intertidal beaver dams in the Pacific Northwest, from British Columbia to Oregon, which shows that tidal beaver are widespread in the region. Region-scale comparisons are also needed to understand variation in beaver behavior that might be associated with system variation, e.g., large rivers with their greater freshwater discharge provide more freshwater tidal habitat, while small coastal wetlands with limited freshwater inflow may be more saline and thus more challenging habitat for beaver. Both foci support the argument that tidal wetlands are not marginal habitat for beaver, rather, they are a place where highly adaptable beaver can thrive. They are also places where we need to pay more attention to the ecosystem role of beaver.

## Methods

### Study areas

This study was focused on the tidal wetlands at the mouths of the two largest rivers in Puget Sound: the Skagit and Snohomish rivers, which respectively deliver 34% and 19% of the freshwater input to Puget Sound as a consequence of mean annual flows of 475 and 270 m^3^s^-1^ [10]. The Skagit River has a classic, triangular delta lying on top of extensive lahar deposits from volcanic eruptions 6000 and 2000 years ago that extended the river mouth 25 km into Puget Sound and created extensive, low, flat areas [11–13]. Most of the delta has been isolated from riverine and tidal influence by levees and dikes, and converted to agriculture and other uses. Consequently, the historically dynamic delta has been tamed by anthropogenic flood control so that geomorphic activity has been reduced and constrained to the two active sub-deltas at the termini of the two principal river distributaries (branches), the North Fork and South Fork, of the Skagit River where historical tidal marsh remains and where marsh progradation has since occurred [14]. The Snohomish estuary is comparatively narrow, 3–4 km wide and 20 km long, and confined by hills on both sides all the way to Puget Sound. Within this stretch, the river consists of anastomosing distributaries in a tidal floodplain dissected by blind-end tidal channels (henceforth, “tidal channels”).

Coastal systems of the Pacific Northwest are generally characterized by mixed semidiurnal tides, i.e., two high and two low tides per day of unequal range. The mean diurnal tide range (difference between higher high and lower low tides) in the Skagit and Snohomish deltas is 3.6 m, but this can vary geographically in Puget Sound from 2.6 m in the north to 4.6 m in the south [15]. Mature marsh surfaces are generally at the elevation of mean higher high water (MHHW), i.e., the average of the daily higher high tides, but during higher high spring tides, marsh surfaces can be inundated by up to 1 m of water, depending on marsh location and elevation. Salinity in coastal wetlands is inversely related to the amount of freshwater inflow and with distance from the mouth of the estuary. These salinity patterns influence vegetation zonation, with salt tolerant species typical of small coastal systems with limited freshwater inflows, and freshwater marsh and swamps (dominated by woody species) typical of systems with significant freshwater inflows or areas further inland along the estuarine salinity gradient.

Field surveys were focused on tidal shrub and Sitka spruce (*Picea sitchensis*) dominated zones of the Skagit and Snohomish deltas, because previous work [8] and local field experience showed that beaver dams and lodges were usually absent in tidal marshes dominated by herbaceous plants such as the sedges and bulrushes (Cyperaceae) that are typical of Pacific Northwest oligohaline tidal marshes. Tidal shrub habitat provides the woody building material that beaver need for dam and lodge construction. Thus, beaver infrastructure is rarely far from tidal shrub habitat. Tidal shrub and forest are found in three principal areas of the Snohomish Delta: the Quilceda marsh near the mouth of the river; Heron Point, about 5300 m upstream from the river mouth; and Otter Island, 5600 m upstream of the river mouth. These areas are separated by herbaceous marsh that were historically flood-breached and abandoned farmlands, and various intentional tidal marsh habitat restoration sites. The Quilceda marsh consists mostly of herbaceous marsh dominated by sedge (*Carex lyngbyei*), soft-stem bulrush (*Schoenoplectus tabernaemontani*), and non-native cattail (*Typha angustifolia* and *T.* x *glauca*) in increasing order of marsh elevation. At still higher elevations, and in a smaller portion of the marsh, typical shrubs such as willow (*Salix* spp.), black twinberry (*Lonicera involucrata*), and wild rose (*Rosa nutkana*) are found, followed finally by Sitka spruce. Heron Point consists mostly of Sitka spruce tidal swamp with an understory of shrubs, while Otter Island is mostly shrub dominated, especially wild rose, with about a quarter of the island comprised of Sitka spruce tidal swamp.

Compared to the Snohomish, the Skagit delta marshes in the North Fork and South Fork sub-deltas are a patchier mosaic of herbaceous and shrub vegetation, with scattered Sitka spruce trees. Shrubs and trees predominate along channel banks, but some channels can have one bank with woody vegetation and the other with herbaceous cover. Unlike the Snohomish, sweetgale (*Myrica gale*) is common in the Skagit marshes and forms dense, nearly impenetrable thickets. This aromatic shrub is shaded out by willows at slightly higher elevations. Wild rose is not as common in the Skagit as the Snohomish delta. More information on the shrub and tree communities in the Skagit can be found in [16].

Within the South Fork sub-delta there are several areas that have been restored from agricultural use to tidal wetlands to provide rearing habitat for juvenile Chinook salmon, a threatened species and an important regional food source and cultural icon. One of these areas, Milltown Island, had its protective dikes breached by a flood in the 1970s, and was subsequently abandoned and allowed to passively return to tidal wetland habitat. Because of its relatively high elevation in the tidal frame, willows have colonized significant portions of the site, as have beaver. Thus, beaver dams and lodges were surveyed on this site to compare with reference marshes in the Skagit and Snohomish deltas.

### RTK-GPS field surveys

Field surveys were conducted at low tide with a real-time kinematic (RTK)-GPS (3-cm horizontal and vertical resolution) in the Snohomish delta (October 2023; March, April, June 2024), the North Fork sub-delta (September 2023; April 2025), and Milltown Island (September 2020). Elevations were referenced to the North American Vertical Datum of 1988 (NAVD88). Tidal channels in the Snohomish and Skagit delta study areas were chosen for accessibility due to logistic and safety challenges working in a meso- to macro-tidal environment. Milltown Island, on the other hand, was almost completely censused except for a few areas where access was impossible due to deep water (even at low tide) and dense shrub thickets. Other dams and lodges were mapped in the South Fork sub-delta in an older effort using hip-chains and field photos [8]; the South Fork data set was periodically updated as incidental field observations during other work produced new sightings of intertidal beaver dams and lodges. RTK-GPS surveyed channel profiles included measurements on the channel bottom, the water surface at low tide, channel banks, the lowest top of beaver dams (where water would spill over), and the ground elevation and tops of beaver lodges (to estimate lodge height). Beaver scent mounds were rarely observed, but when they were, they were also located with RTK-GPS. ArcMap GIS was used to plot RTK-GPS data, generate channel profile traces, and produce maps of the field data.

### Statistical analysis

Simple linear regression was done using an on-line calculator [17]. Residuals were evaluated for deviations from normality by the Shapiro-Wilk test and examination of the residuals plot. Analysis of Covariance (ANCOVA) was done using an on-line calculator, with normality and homoscedasticity checked by the Shapiro-Wilk and Levine’s test, respectively [18].

G-tests of goodness of fit were done using the Statology calculator [19] and applied to comparison of the count of movement routes from observed beaver lodge locations vs. the locations of random points on channel banks. Random points were generated in GIS (ArcGIS Pro, ESRI) using the “Create Random Points” geoprocessing tool, applied independently to a shapefiles of the Skagit River distributaries and of Skagit Delta saltmarsh tidal channels. For each shapefile, 100 points were generated and the number of movement routes within 15 m and within 50 m were counted for each observed lodge and each random point. The observed lodge count for each category of route count was sometimes too low for G-test analysis, so this count was standardized by conversion to a percentage of the total number of observed lodges and multiplied by 100 for comparison with the 100 random points. Categories of route counts that still had too few observations for analysis were lumped together in logical groups to allow analysis.

## Results

### Local-scale field surveys

**Fig 1:**
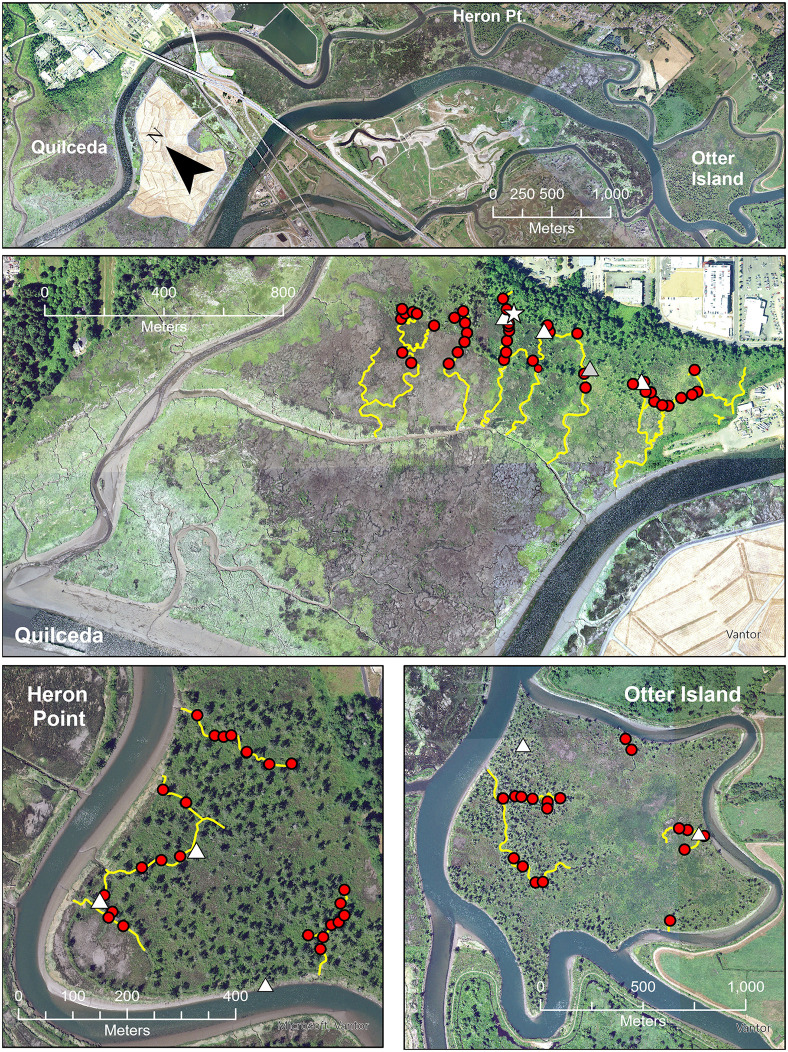
Overview of beaver dam and lodge locations in the Snohomish estuary. Yellow lines are traces of field-surveyed tidal channels; red dots are beaver dams; white triangles are beaver lodges; gray triangle is an abandoned lodge; white star is a scent mound. Areas without tidal shrub vegetation were not surveyed, because previous work had shown that beaver structures were absent there. Map Data © 2019 Google.

Intertidal beaver dams and lodges (S1-S9 Figs in S1 File) were surveyed in three locations in the Snohomish Delta. In the Quilceda marsh, near the mouth of the river, 41 intertidal dams and 3 lodges were encountered in a little over 2000 m of tidal channels in the portion of the marsh that was characterized by tidal shrub vegetation and Sitka spruce trees (Fig 1). Due to challenging field conditions only 67% of the terrain could be sampled, so total dam and lodge counts could be 50% higher than observed. At Heron Point the surveyed tidal channels were extensively dammed, and flooded understory channel-ponds ramified throughout. Thus, unsurveyed channel was similarly difficult to assess as for Quilceda, but at least 1000 m of channel could be detected in aerial photographs that were not surveyed for beaver. Thus, only 45% of the available tidal channel may have been surveyed, so that almost twice as many dams and lodges may be present here as observed. Only a portion of Otter Island is densely covered by Sitka spruce canopy; the rest is covered in a variety of water-tolerant shrubs. Thus, aerial photo inspection could identify approximately 2000 m of likely channel that was not surveyed, so total dam and lodge count could be as much as 2.4 times what was observed.

**Fig 2:**
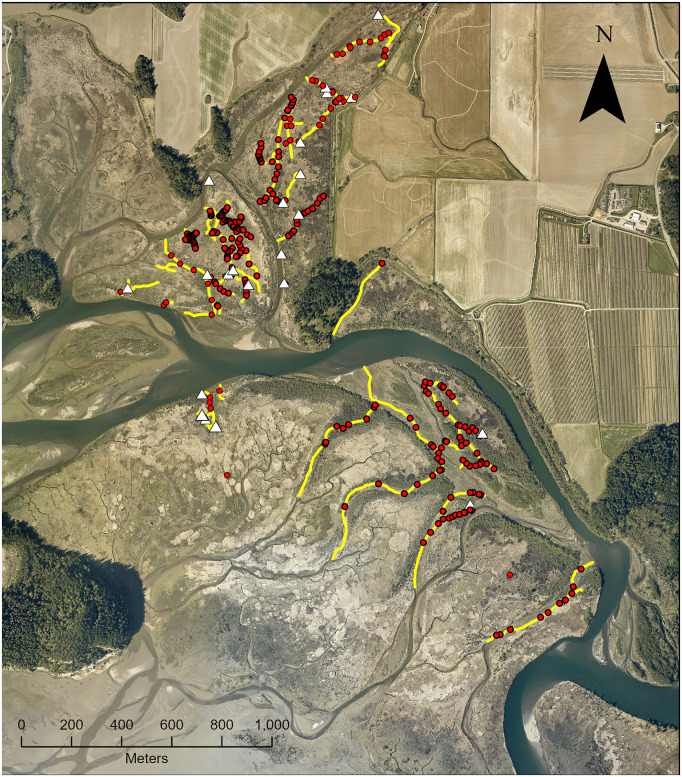
Overview of beaver dam and lodge locations in the North Fork Skagit sub-delta tidal wetlands. Yellow lines are traces of field-surveyed tidal channels; red dots are beaver dams; white triangles are beaver lodges. Areas without tidal shrub vegetation were not surveyed, because previous work had shown that beaver structures were absent there. Heavily forested areas are non-tidal, rocky outcrops. River flow is from the east side of the frame. Farmland borders the study area to the north. Imagery © 2021 Eagleview.

**Fig 3:**
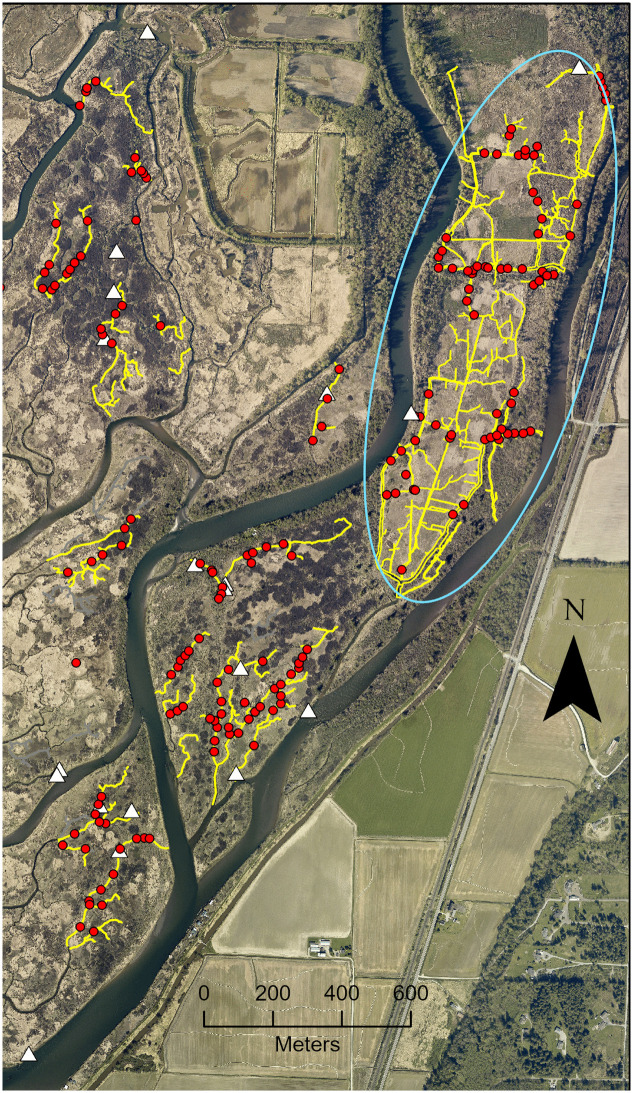
Tidal beaver structures in the eastern half of the South Fork Skagit delta marshes. Yellow lines are surveyed channel traces; red dots are beaver dams; white triangles are lodges. The Milltown Island restoration site is highlighted (blue oval). Imagery © 2021 Eagleview.

The North Fork and South Fork Skagit tidal marshes are a mosaic of shrub and herbaceous marsh, so total unsurveyed channel could be confidently estimated from aerial photographs. In the North Fork sub-delta, approximately 5100 m of likely channel were not surveyed, nearly half the amount that was surveyed (Fig 2), so total dam and lodge count could be 50% higher than observed. In the larger South Fork sub-delta, 28.7 km of tidal shrub channel were not surveyed compared to the 13.5 km that were surveyed (Fig 3), so total beaver dam and lodge count could be three times as high as observed. In the Milltown Island tidal habitat restoration site on the east side of the South Fork delta, channel surveys were exhaustive so that 12.0 km of channels were canvassed, while only 1.1 km could not be accessed due to dense shrub thickets and deep water. Thus, it is likely that only 9% of the site’s dams were missed.

Dam density (per km of surveyed tidal channel) ranged from 10.3 to 29.6 km^-1^, and averaged 19.1 km^-1^ for the Snohomish and Skagit reference marshes (Table 1). In contrast the Milltown Island restoration site value was 6.0 km^-1^, less than 1/3 of the reference marsh average. Lodge density ranged from 1.4 to 3.7 km^-1^, and averaged 2.1 km^-1^. The value for Milltown Island was 0.25 km^-1^, which is less than 1/8th the reference marsh average. Beaver colonies can occupy more than one lodge, but this study did not distinguish between active versus inactive lodges. For fluvial and tidal colony densities to be comparable, tidal beaver would have to construct 3.4 lodges per colony (*cf*. S1 Table in S1 File).

**Table 1:** Summary statistics on estuarine tidal dam and lodge density.

Location	Channel length surveyed (m)	Count of dams	Count of lodges	Dams per km of channel	Lodges per km of channel
**Snohomish Delta** **Quilceda marsh**	2,010	41	3	20.4	1.5
**Heron Point**	810	24	3	29.6	3.7
**Otter Island**	1,400	18	2	12.9	1.4
**North Fork Skagit delta**	10,520	235	20	22.3	1.9
**South Fork Skagit delta**	13,450	139	29	10.3	2.2
**Milltown Island** ^**a**^	12,020	72	3	6.0	0.25
**Tidal mean (sans Milltown Island)**				19.1	2.1

^a^Milltown Island is abandoned farmland, whose dikes were breached by a flood in the 1970s.

In all surveys of the Skagit and Snohomish deltas, only one bank lodge was found; it was located on a large river distributary levee that was augmented in size by turn of the century (19^th^-20^th^) deposition of dredge spoils to allow ship navigation. All other beaver lodges were constructed on marsh surfaces and consisted of the classic mounds of sticks and mud. Mound lodges were invariably located on the banks of either tidal channels or river distributary channels. Of the 59 lodges found in the Skagit and Snohomish deltas, 16 were found on distributary banks and 43 on tidal channel banks. In the vicinity of the lodges, distributaries averaged 74 m wide (median = 42 m, range = 7–350 m) while tidal channels averaged 3.1 m wide (median = 2.6 m, range = 0.7–19 m).

**Fig 4:**
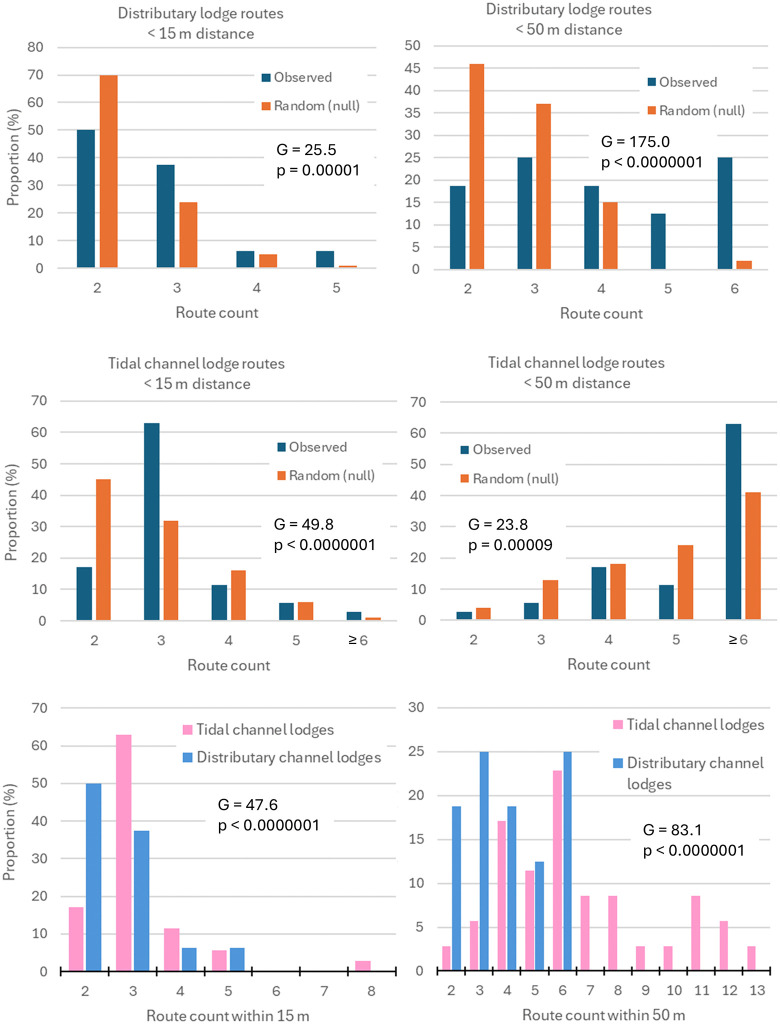
Frequency distributions of the number of aquatic movement routes in the vicinity (< 15 m and < 50 m) of beaver lodges vs. random points located on tidal channel or distributary banks.

Lodges on distributary banks had at least two aquatic movement routes from the lodges, upstream and downstream, but they were often located near distributary junctions or tributary tidal channels, which increased beaver movement options in the lodge vicinity. Lodges located within tidal channel networks were typically located in the vicinity (within 15 m or within 50 m) of tributary junctions, often multiple junctions (Fig 4), which may be a way of maximizing beaver movement routes away from the lodge. Comparison of observed lodge locations with random points suggests that beaver bias the location of their lodges towards sites with high connectivity in channel networks, i.e., the number of potential aquatic movement routes. Their lodges had significantly higher connectivity than points randomly located along channel network banks (Fig 4). This was true at two spatial scales: within 15 m of a lodge (or random point) and within 50 m, and it was true for lodges located on distributary banks as well as tidal channel banks. Tidal channel lodges had much higher connectivity than distributary bank lodges, especially at the 50-m scale, which is consistent with most lodges being located on tidal channels. However, the presence of a large fraction, 27%, of all lodges on distributary banks suggests there may possibly be some compensatory benefit to location on a distributary.

### Channel profiles

**Fig 5:**
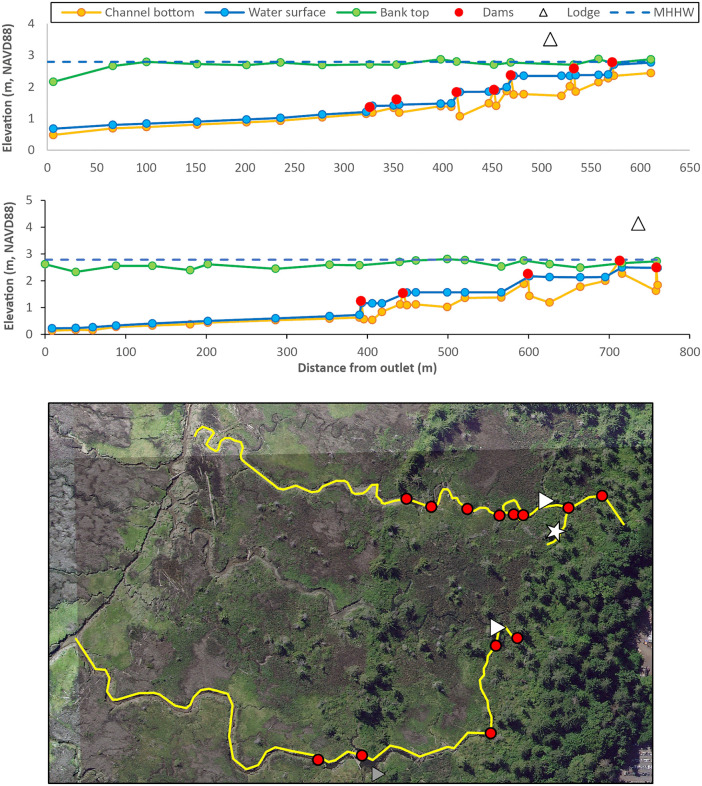
Typical beaver-dammed tidal channel profiles. Water surface elevations are at low tide; compare with mean higher high water (MHHW) [top graphs]. Associated channel traces (yellow lines) are shown over a 2019 air photograph with beaver dams (red dots) plotted [bottom photo]. Other channel trace profile points are not shown to avoid graphic clutter. White triangles are beaver lodges; the small gray triangle is the remains of a former lodge; the white star is a scent mound. Areas further upstream were not mapped because they were diffusely anastomosing, pond-like channels that were difficult to map. Map Data © 2019 Google.

Beaver-dammed tidal channel profiles show striking contrasts between downstream channel reaches that flow through herbaceous marsh and upstream reaches that flow through tidal shrub and Sitka spruce vegetation. The downstream reaches have no beaver dams for 300–400 m, so at low tide there are no pools, and the water is typically 9–15 cm deep while flowing over relatively narrow (< 1-m wide) thalwegs at low tide; the broader (4–8 m wide) channel bottoms outside of the thalweg are essentially drained at low tide. The upstream reaches over the next 300–400 m have a series of five to seven dams that pond water in a stair-step fashion so that the water surface at low tide eventually attains the channel bank top. The most seaward dams are clearly overtopped by flood tides, but the highest, landward dams are likely only overtopped on higher-high spring tides (compare dam height vs. mean higher high water [MHHW] in Fig 5). At low tide, beaver pond depths in the tidal channels typically range from 30 to 95 cm ($\bar{x}$ = 50.6, *n* = 23). Similar profiles can be found in the South Fork Skagit Delta marshes when downstream tidal channel reaches are bereft of tidal shrub vegetation and instead are characterized by typical Pacific Northwest sedges.

**Fig 6:**
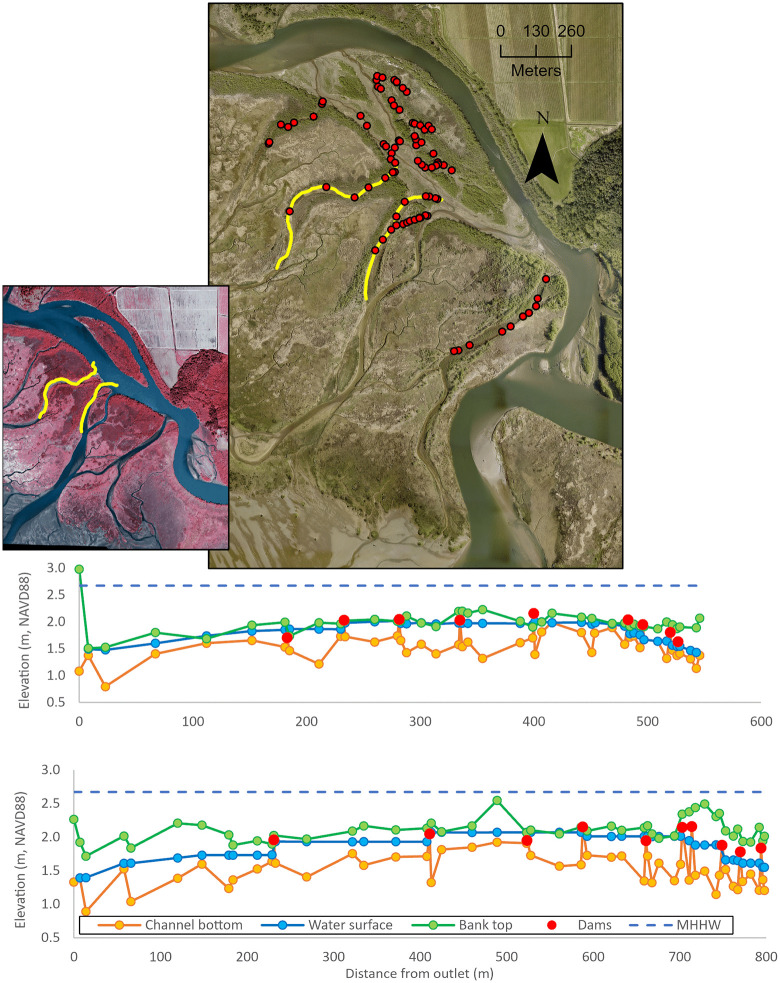
Profiles of two senescing delta distributaries. Surveyed channel traces (yellow lines) are superimposed on a 2021 aerial photo (Imagery © 2021 Eagleview); red dots are beaver dams [top photo]. Insert is 2004 photo prior to distributary senescence (Imagery © 2004 Triathlon). Surveyed channel profiles [bottom graphs] show low-tide conditions. The mature marsh surface is at approximately MHHW (e.g., the first profile point on the top graph), while the marsh surface in the shoaling distributaries is lower. For more examples of beaver channel profiles see S10-S13 Figs in S1 File.

Beaver lodges (S9 Fig in S1 File) were not always located where dams raised low-tide water levels to near the marsh surface, but they were generally located where dams raised the low-tide water level sufficiently to cover the lodge entrances. However, the purpose of tidal beaver dams is not merely to flood lodge entrances, it is also to allow beaver to swim in tidal channels during low tide. This is illustrated by dam construction in two senescing river distributaries in the North Fork Skagit delta where lodges are not present (Fig 6). The North Fork mainstem channel has avulsed (starting in 2004) into a new course that bypasses most of the delta [20]. The avulsion channel has become the new mainstem channel of the North Fork, while the former mainstem channel and its distributaries are filling with sediment and shoaling. Prior to shoaling the distributaries hosted no beaver dams, likely because they were deep enough to contain substantial subtidal waters that allowed low-tide beaver movement, and because their large size allowed tidal flows or river floods strong enough to wash away beaver dams. As the distributaries have shoaled, they have also narrowed and filled with vegetation, but the aggrading vegetated surface is still lower than the adjacent, older marsh platform. Thus, these shoaling distributaries do not host any beaver lodges, neither on their current low-elevation banks (because the lodges would be flooded at high tide), nor on the nearby older, pre-avulsion distributary banks (one such pre-existing lodge was abandoned because it became land-locked). Because the shoaling distributaries drain in two directions at low tide, the distributary channel profiles are convex upward, and the beaver dams create low-tide pools that never stair-step the water surface elevation up to MHHW, the approximate elevation of a mature marsh platform. The only possible function of these dams for beaver is to create pools that allow low-tide beaver movement.

Intertidal beaver dams were always constructed of mostly sticks, from nearby shrubs, and supplemented with mud. Sometimes bank slumps were incorporated into dams, suggesting beaver opportunistically taking advantage of such situations to reduce construction effort. On one occasion a derelict boat that filled a tidal channel was incorporated into a large beaver dam. Dam heights ranged from 5 to 172 cm and averaged 48 cm (Table 2). This average is a little over half the average height reported for fluvial dams (Table S2 in S1 File). Water head for tidal beaver dams ranged from 0 cm for dams in disrepair to 134 cm, with an average of 13 cm. If dams in disrepair are ignored, then the average water head is 25 cm, which is 78% of the average reported for fluvial dams. Intertidal beaver dam pool depths ranged from 4 to 137 cm and averaged 51 cm, which is 75% of the average for fluvial beaver dam pools. Intertidal beaver dam pools are generally linear in form, i.e., tidal channels that are not allowed to drain on low tide. But some dam pools flood beyond the banks of the tidal channels into adjacent topographic lows, so they are more irregular in shape. There is likely some negative bias in the tidal beaver dam pools size estimates because they were measured in GIS from air photos, so canopy cover prevented measurement of some pools, and very narrow channels were a challenge for distinguishing pools. Reported fluvial beaver dam pool areas averaged about 50% larger than tidal beaver dam pools.

**Table 2:** Beaver dam and pool metrics for tidal systems. Mean (standard deviation) and range of tidal beaver dam water heads and heights, and pool depths and areas, as measured from surveyed tidal channel profiles and GIS analysis of aerial photographs. *N* is the sample size for each metric, respectively. Fluvial weighted means from Table S1 in S1 File are shown for comparison.

Site	*N*	Water head (cm)	Dam height (cm)	Pool depth (cm)	Pool area (m^2^)
Snohomish Delta – Quilceda	24, 27, 27, 21	36 (19) 10–85	57 (24) 21–114	47 (21) 15 - 95	70 (73) 10 - 356
Snohomish Delta – Heron Point	19, 21, 18, 8	45 (37) 8 - 127	71 (31) 23–141	68 (23) 26 - 105	49 (33) 21 - 109
Snohomish Delta – Otter Island	17, 19, 13, 15	34 (34) 7 - 134	73 (36) 23 - 189	70 (22) 21 - 101	251 (173) 24 - 540
Skagit Delta – North Fork	143, 223, 216, 62	19 (12) 7 - 63	43 (26) 5 - 143	49 (25) 4 - 137	120 (165) 3 - 874
**Tidal weighted mean** ^**a**^		**25**	**48**	**51**	**123**
**Fluvial weighted mean** ^**b**^		**32**	**82.3**	**68**	**184**

^a^Weighted by sample size; ^b^See Table S1 in S1 File for more details.

It is reasonable to think that channel size could affect beaver dam construction. If we have a simplistic hypothesis that assumes tidal dams are generally relatively uniform in design (with some variation around a mean), then each dam would have a similar height and water head, so that a nearly constant number of dams would be found in tidal channels regardless of channel size (length), because the difference between low tide channel bottom elevations and MHHW is similar for almost all channels (except small tributaries to tidal channels). If true, then short channels should have steeper gradient and closely spaced dams while long tidal channels should have lower gradient and more widely spaced dams.

**Fig 7:**
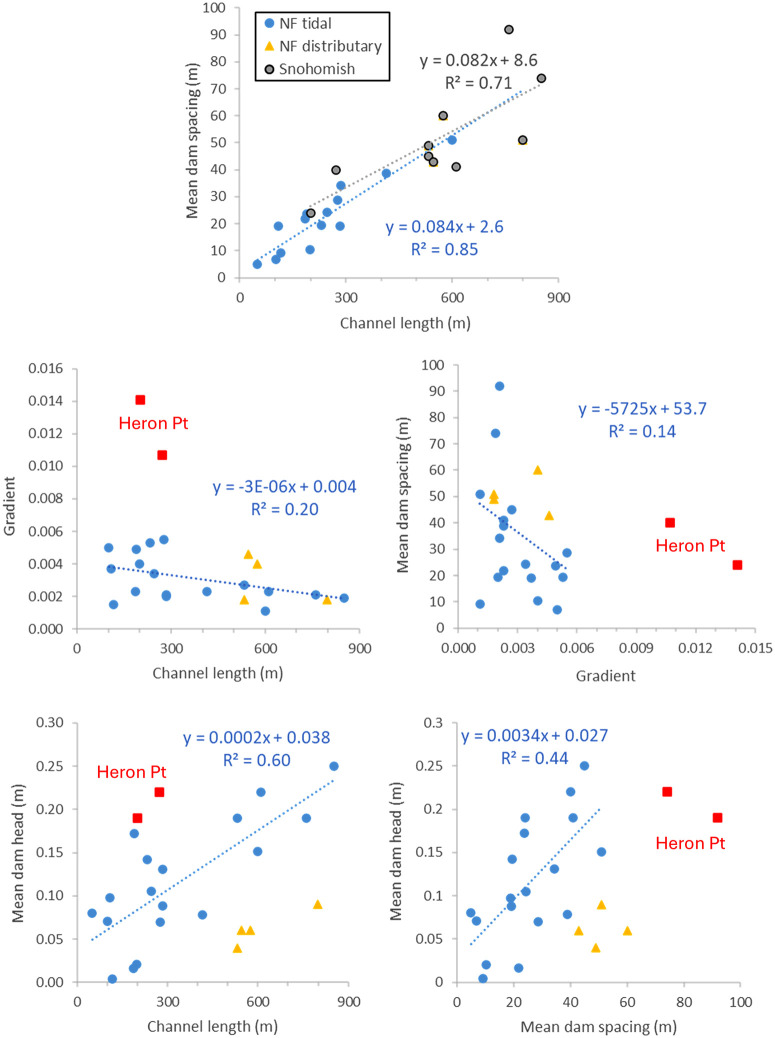
Exploratory regression analysis of beaver dam spacing and dam head relative to channel length and gradient for North Fork (NF) Skagit tidal channels, NF senescing distributary channels, and Snohomish tidal channels.

Exploratory statistical analysis indicated that the spacing of beaver dams was not uniform or random, but instead increased with channel length (Fig 7), consistent with the hypothesis. The linear regression relationship was calculated for the fourteen North Fork Skagit Delta tidal channels (R^2^ = 0.85, p < 0.000004) and the six Snohomish tidal channels (R^2^ = 0.71, p < 0.035), with ANCOVA showing no significant difference in dam spacing between the two groups (F_1, 17_ = 0.96, p = 0.34), but significant influence of the covariate, channel length (F_1, 17_ = 55.2, p < 0.001). Points for the senescing North Fork distributary channels plotted in the vicinity of the two regression lines.

In addition to channel length, channel water surface gradient might also correlate with beaver dam spacing, so gradient was compared to channel length. The two Heron Point channels (Snohomish Delta) were distinct outliers from all other channels, with gradients four times the average of the other channels. For the other channels, channel length was inversely related to gradient, as expected, but only explained 20% of the variance in gradient (R^2^ = 0.20, F_1,19_ = 4.89, p = 0.04). Nevertheless, dam spacing was not related to channel gradient (R^2^ = 0.14, F_1,19_ = 2.93, p = 0.10), although the trend appeared consistent with the hypothesis.

Because the Quilceda Marsh (Snohomish) channels had similar gradients as the North Fork Skagit channels, they were no longer distinguished in subsequent analyses, but Heron Point channels continued to be unexpected outliers. Channel length and mean dam spacing each affected mean dam head (R^2^ = 0.60, F_1,16_ = 23.9, p = 0.0002 and R^2^ = 0.44, F_1,16_ = 12.5, p = 0.003, respectively). The dams in the senescing distributaries were also outliers, but this was unsurprising given the immature morphology of these channels, whose banks were well below MHHW. Maximum dam head showed similar relationships to channel length and mean dam spacing, indicating that dams are not generally uniform as assumed in the hypothesis, but instead respond to channel size. Muddier sediments in the Snohomish Delta compared to the sandier Skagit, and on the convex bank of a Snohomish distributary meander (Heron Point) may allow construction of taller and less leaky dams in the Heron Point marshes.

### Region-scale aerial photo surveys

Aerial photo inspection to identify likely locations of beaver dams and lodges could allow planning of focused field work to maximize beneficial effort. To this end, aerial photo

interpretations of likely dam and lodge locations were compared to field observations, and the degree of agreement was summarized in error matrices (Table 3). Only dam presences were predicted from aerial photo interpretation, not dam absences, to avoid inflating estimates of prediction accuracy. Thus, the error matrices are missing the quadrant containing verified negative predictions.

**Table 3:** Error matrices for aerial photo interpretation of tidal beaver dams. “Predictions” are aerial photo interpretations; “Observations” are ground-truthed.

	*North Fork Skagit Delta*		
	Dam observed	Dam not observed	Total
**Dam predicted**	69	52	121
**Dam not predicted**	197	–	
**Total**	266		
	*Snohomish Delta*		
	**Dam observed**	**Dam not observed**	**Total**
**Dam predicted**	31	20	51
**Dam not predicted**	57	–	
**Total**	88		
*Fraser, Nisqually, Chehalis, Grays Harbor, Willapa Bay, Oregon Coast*			
	**Dam observed**	**Dam not observed**	**Total**
**Dam predicted**	43	1	44
**Dam not predicted**	14	–	
**Total**	57		

True dams were 57% of all predicted dams for the North Fork Skagit Delta and 60% for the Snohomish Delta. Predicted dams were 26% of all field-observed dams for the North Fork Skagit Delta and 35% for the Snohomish Delta, which means that when using aerial photos, I identified only 1/4–1/3 of the dams that were actually present on the landscape. The sources of error in photo interpretation stem primarily from the presence of shrub canopies that obscure channels, location of small dams in narrow, steep-sided channels that makes their observation difficult, and general photo ambiguity (Table 4). Photo ambiguity generally consisted of difficulty in distinguishing a dam from an accumulation of wrack or sticks in a tidal channel, or of distinguishing a dam from a local high spot in the channel bottom that has been exposed by low tide.

**Table 4:** Sources of photo interpretation error for tidal beaver dam prediction. “Success” = true positive; “Fail” = false positive; “Missed dam” = false negative.

North Fork Skagit Delta	Success	Fail	Missed dam	TOTAL
**Ambiguity**	31	39	84	154
**Shrub canopy**		5	45	50
**Small channel**		4	64	68
**Classic impoundment**	29			29
**Visible dam**	5			5
**TOTAL**	65	48	193	306
**Snohomish Delta**	**Success**	**Fail**	**Missed dam**	**TOTAL**
**Ambiguity**	19	18	17	54
**Shrub canopy**		1	34	35
**Small channel**	1	1	6	8
**Classic impoundment**	11			11
**Visible dam**				
**TOTAL**	31	20	57	108

**Fig 8:**
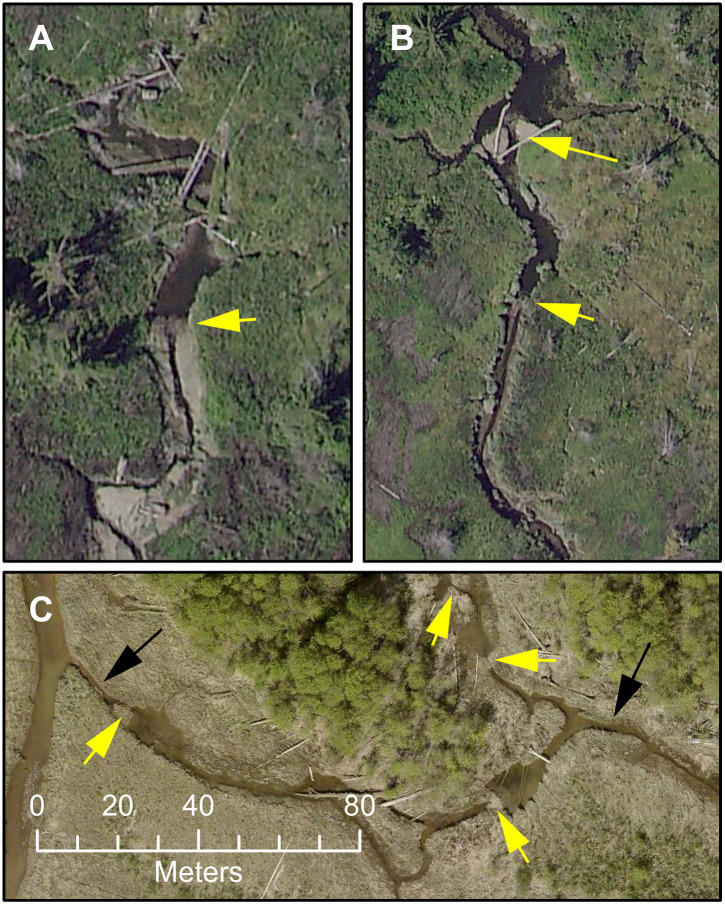
Examples of photo interpretation of intertidal beaver dam locations. All photos are at low tide. Yellow arrows indicate correctly predicted dams; black arrows indicate missed dams, i.e., not predicted, but observed in the field. Photos A and B are examples from the Quilceda marsh in the Snohomish estuary, where ebb-tide flow is from top to bottom in each photo (Map Data © 2022 Google). Photo C is from the North Fork Skagit sub-delta, where ebb-tide flow is from right to left (Imagery © 2023 Eagleview).

Successfully predicted dams were most confidently identified by clearly visible dams and by photo-signatures I termed “classic impoundments”, which consisted of a combination of a channel obstruction that demarked a narrow downstream channel from a strikingly wider upstream channel (Fig 8). When the dam was the first in a series, the downstream channel often had clearly exposed substrate at low tide, while the upstream channel was clearly inundated.

**Fig 9:**
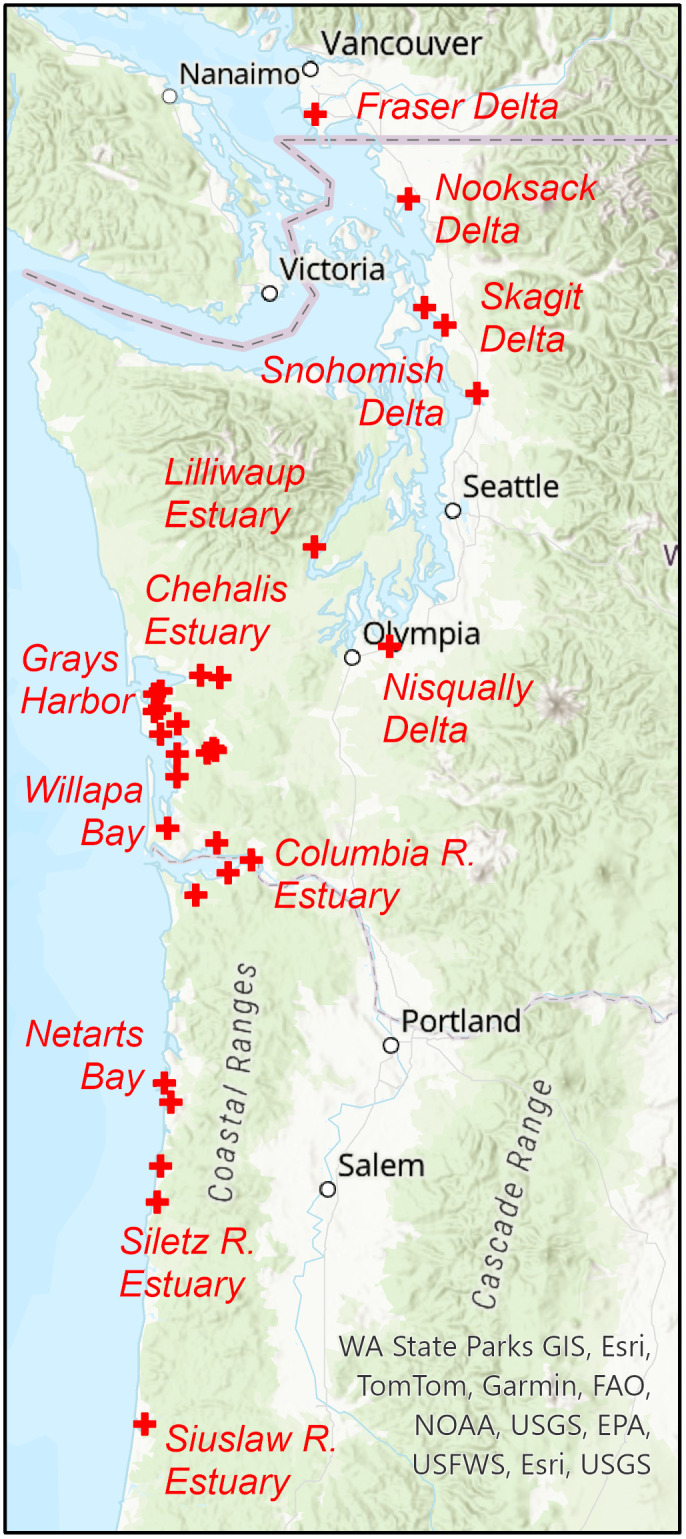
General locations of confirmed and likely presence of intertidal beaver dams in the Pacific Northwest (red crosses). See Supplementary Materials for more specific locations.

The lessons learned from the error analysis were applied to a search of Google Earth imagery for intertidal dams in other coastal wetlands in the Pacific Northwest to determine how widespread the distribution of tidal beaver might be. Google Earth photos from 2012 to 2024 (earlier photos generally had insufficient resolution to be useful) were examined for reliable indicators of tidal beaver dams. Ambiguous photo-signatures, canopy covered areas, and narrow channels were ignored in favor of clearly observable dams and classic impoundments. Intertidal beaver dam photo-signatures were observed from the Fraser River delta near Vancouver, Canada to the Siuslaw River estuary, Oregon (Fig 9), where mean diurnal tidal range varies from 2.6 to 4.6 m in the Salish Sea, and from 2.1 to 3.1 m on the outer coasts of Washington and Oregon. Ground-truthing of a sample of these conservatively identified dams revealed much lower rates of false positives, i.e., 43 out of 44 photo-based predictions were verified in the field (the one error was due to anthropogenic widening of a short channel reach), but 14 of 57 dams observed in the field were still missed in photo examination (Table 3).

**Fig 10:**
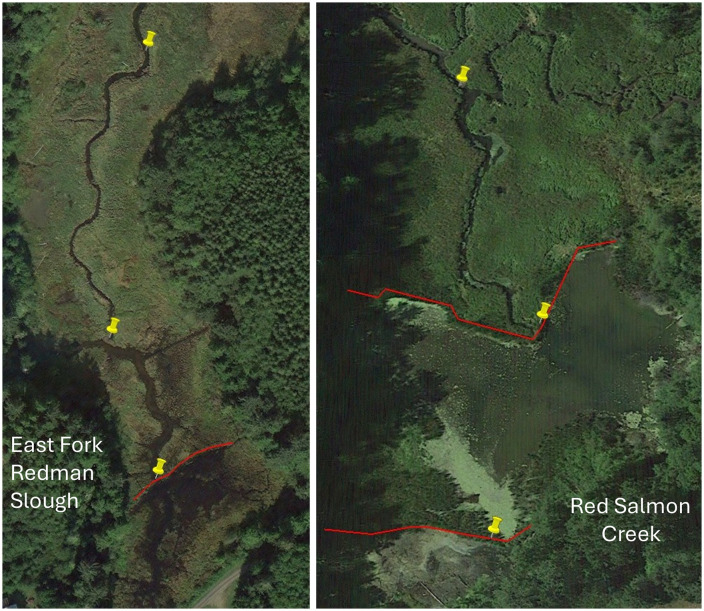
Examples of marsh surface dams (red lines) and impoundments for small tidal stream systems. Channel spanning dams are noted by yellow push-pins in low-tide Google Earth imagery. Redman Slough and Red Salmon Creek were ground-truthed. For scale, the Redman slough surface dam was 65 m long; the longer Red Salmon Creek surface dam was 120 m long. Map Data © 2016 Google.

These coastal systems can be classified into two broad geomorphological categories: (1) large river systems with substantial freshwater input (e.g., Fraser, Nooksack, Skagit, and Snohomish deltas; Chehalis, Willapa, Columbia, Gray’s, Young’s, Salmon, and Siletz river estuaries), and (2) small coastal streams with modest freshwater input. The structure of beaver dam networks in large river systems has already been described earlier using the Skagit and Snohomish deltas as examples, but in small coastal streams the beaver dam network is sometimes different. There, a few ordinary channel-spanning tidal dams may precede a large, ½-m to 1-m high, marsh-spanning dam that impounds a large pond on the marsh surface (Fig 10). These marsh surface dams ranged from 40 m to 120 m long and impounded ponds that ranged from 400 m^2^ to 6000 m^2^ in surface area. The ponds typically had a small tributary creek that provided freshwater inflow.

Being able to confidently identify tidal beaver dams in aerial photos allows estimation of minimum dam age from available historical aerial photos, where the estimate depends on image quality. Examination of aerial photos shows that Pacific Northwest tidal beaver dams can easily reach a couple of decades in age; in a couple of locations, they are at least 35 years old (Table 5).

**Table 5:** Estimated minimum tidal beaver dam age from earliest observation in an aerial photograph.

Location	Earliest observation	Latest absence	Minimum age
**Fraser Delta**	2004	–	21
**Snohomish-Quilceda**	2003	1990	22
**Snohomish-Otter Isl.**	2010	–	15
**Skagit North Fork** ^**a**^	2004	–	21
**Nisqually**	2005	2003	20
**Chehalis**	2006	–	19
**East Fork Redman Slough**	1990	–	35
**Johns River trail sloughs**	1990	–	35
**Elk River Estuary**	2011	–	14
**Columbia Estuary**	2012	–	13
**Youngs River**	2004	–	21

“Latest absence” refers to the oldest photograph in which the beaver dam is not observed. Dam age is thus bounded by earliest observation and latest absence. Latest absence is not noted when photo quality was too poor to reliably indicate that dam was present or absent.

^a^An associated lodge was also clearly visible and present since 2004.

Lodges are nearly impossible to identify from aerial photographs because they are typically either obscured by shrub canopy, or their coloration differs little from background vegetation. They were successfully predicted only three times, out of 45 lodges found in the Skagit tidal wetlands and 8 found in the Snohomish tidal wetlands, on the three rare occasions when there was a suspicious contrast in coloration.

## Discussion

Beaver have been considered a riverine and lacustrine species, but recent observations indicate they can also inhabit estuarine systems with significant tidal ranges (up to 5 m). This paper clearly documents the presence of beaver in tidal wetlands from the Fraser Delta in southern British Columbia to the Siuslaw estuary midway along the Oregon coast. This is likely not just a west coast phenomenon as beaver have also been observed in tidal wetlands of the Tivoli Bays in the Hudson River National Estuarine Research Reserve (New York) where there is a 1.7-m tidal range (Jack Loud, Bard College student, personal communication; S38 Fig in S1 File).

Tidal beaver dams and lodges are abundant in large river deltas and estuaries such as the Snohomish, Skagit, Fraser (S14-S16 Figs in S1 File), Chehalis (S19-S22 Figs in S1 File), and Columbia (S33 Fig in S1 File), which have abundant freshwater discharge, low marsh salinities (fresh to oligohaline), and abundant woody vegetation in the tidal zone. In these systems, tidal beaver dams and lodges are found in close association with tidal shrub and tidal forest vegetation. More surprisingly, beaver can also be found in smaller coastal systems with sparse patches of woody vegetation or woody vegetation along the upland margins of the tidal marsh. To find woody material to build a dam, beaver have to travel as far as 70 m, 74 m, and 200 m from a tidal dam to a forested border for Netarts Bay (S34-S35 Figs in S1 File), the Siletz estuary (S36-S37 Figs in S1 File), and a marsh along the Johns River (S23-S24 Figs in S1 File), respectively, although almost all of that distance can be traversed by tidal channels. In contrast to larger river deltas or estuaries with high freshwater inflows and large coastal freshwater plumes, beaver in small coastal tidal systems may have limited scope in downstream movement because of high downstream salinities. To compensate, beaver in small, coastal, tidally-influenced streams with small freshwater inflows may impound large ponds with marsh surface dams to provide greater amounts of freshwater habitat. The in-channel dams downstream of the marsh-surface dam may not only assist in damming the upstream pond, they may also serve as salinity barriers by blocking the densest, saltiest tidal floodwaters. While small coastal systems may have a relatively small amount of freshwater input, inland portions of the systems may be stratified, with lighter freshwater floating on top of denser saltwater, so that beaver experience minimal salt stress. Vegetation observed in the more landward portions of these small coastal systems was invariably typical of fresh to oligohaline tidal marshes.

Tidal habitat use by beaver may appear surprising given more common observations in fluvial systems, but this perception may be a consequence of range contraction following near extinction of the species by 19^th^ century trapping, as well as greater habitat loss in estuarine ecosystems (e.g., 85% loss on the west coast [21]) compared to riverine and lacustrine systems. Tidal shrub and forest wetlands, preferred tidal beaver habitat, have been disproportionately lost compared to tidal emergent marsh [22]. Compared to tidal marsh, these areas are also more challenging to human movement and are thus less studied. Given the abundance of beaver dams and lodges in tidal wetlands throughout the Pacific Northwest, and the abundance of food resources for beaver in these wetlands, it seems unlikely that tidal wetlands are sub-optimal beaver habitat compared to “normal” fluvial habitat. They are simply additional, but different habitat. However, conclusively demonstrating this will require collecting data on body condition and breeding statistics for tidal beaver.

### The role of tidal beaver dams

From the perspective of a beaver, the function of a dam is to provide water deep enough to inundate lodge entrances and allow beaver to safely swim to foraging sites [23]. This is true in fluvial systems and appears to also be the case in tidal systems, except that in tidal systems the need for inundation is focused on low tides. During high tides, tidal channels and most beaver dams are completely inundated. Indeed, the marsh surface itself can be flooded by up to a meter, depending on the tide, weather, and location in the estuarine elevation gradient. Thus, during high tides, beaver dams are generally irrelevant. However, during low tides, beaver dams shine. Channels without beaver dams often convey shallow streams of water, as little as only a few centimeters deep. In contrast, channels with beaver dams have low-tide pools averaging 51 cm deep, enough for beaver to swim at low tide. Because low-tide beaver dams can quadruple the length of low-tide pools compared to channels without beaver dams (other pools can be formed by scour in meander cut banks, at tributary junctions, and around logs [8]), their role in increasing beaver mobility at low tide appears to be significant. This is starkly illustrated in the profiles of the Quilceda tidal channels, where the downstream half of the channels have no beaver dams and convey a relative trickle of water, while the upstream half is impounded by a series of dams that provide almost continuous swimming routes, interrupted only by the dams themselves.

Contrasts between the Quilceda channel profiles and those of the senescing North Fork Skagit distributaries raise the question of beaver mobility, especially during low tide. All of the Quilceda channel profiles are without dams in their lower half, and consequently have water depths of only a few centimeters there. This suggests that beaver residing in this area have relatively restricted movements, at least during low tides, moving around only in the upper half of the channel networks where dams pond water at low tide. The North Fork Skagit channel profiles, on the other hand, have dams down to or near the channel outlets, and the senescing distributaries have dams well distant from any lodges or tidal channel networks. This suggests that beaver in the North Fork delta are potentially more mobile during low tides, traveling farther from their lodges and traveling through river distributaries, even those that are senescing and shoaling. Why the apparent difference between Quilceda and the North Fork Skagit? Habitat does not appear to be limiting; there is an abundance of vegetation to eat in the North Fork delta, especially willows. Are population densities higher in the North Fork? Or are the Quilceda marshes distinctive because they are located so close to the river mouth and experience relatively high salinity? During summer low river flows the salinity in this area averages 18.7 ppt, while Heron Point and Otter Island average 9.4 and 3.8 ppt, respectively [24]. The Skagit marsh areas that host beaver are typically 0–5 ppt during summer low flows. Perhaps the high late-summer salinities discourage Quilceda beaver from venturing far from their Sitka spruce strongholds and produce the more restricted distributions of their dams.

Beaver dams in fluvial systems have ecosystem roles beyond those intended by beaver; plant and wildlife species richness increase as a result of beaver dam ponding, and fluvial beaver dams can more generally affect stream hydrology, geomorphology, and ecosystem function [5]. Tidal beaver dams increase low-tide, volumetric fish density by 3- to 7-fold depending on species, indicating that tidal beaver ponds may provide important low-tide refuge for many fish [8]. In the current study, dam functions were not evaluated, but incidental observations suggest at least some dams, typically those higher in the channel network, can accumulate significant amounts of unconsolidated sediment. Walking through those dam ponds was hazardous, with soft sediments a meter or more in depth. Several small, headward channel reaches in the North Fork delta were so full of semi-consolidated sediment that mud reached to within a half meter of the marsh surface and the channels appeared to be in the process of senescing to the point of disappearance as a result of sediment trapping by the beaver dams. Tidal beaver dams appear to trap abundant organic detritus as well as mineral sediments, and this may result in high production of invertebrate detritivores [8]. Low-tide beaver ponds may also increase the residence time of juvenile salmon and other fish in the channel networks; and predation on fish in these ponds may be relatively low. Finally, a provocative thought is that beaver dams might serve as salinity barriers that reduce saltwater intrusion into tidal channel networks. Because saltwater is heavier than freshwater, a series of beaver dams might sequentially freshen tidal flood waters along a tidal channel profile. Perhaps this is why beaver, and Sitka spruce, can inhabit the brackish Quilceda marsh. Clearly, there is scope for a great deal of further investigation into the possible ecosystem roles of tidal beaver dams and their low-tide ponds.

### Single large or several small beaver dams

In many tidal channels, a series of beaver dams stair-steps water up to marsh surface to create extensive areas of constant ponding, even during low tides. Why does stair-stepping occur? Why not just build one tall dam that ponds up water throughout the tidal channel? This option was observed, but only once, on the east side of Otter Island in the Snohomish Delta where a nearly 2-m tall beaver dam was located 20 m from the channel outlet. At low tide, the water head for the dam was 1.38 m, forming an 87-cm deep pool upstream of the dam, compared to a trickle of water downstream (the difference between dam head and pool depth suggesting perhaps 51 cm of sediment trapping in the pool). More commonly, intertidal beaver dams occur in a series and average 48 cm tall with water heads averaging 25 cm (i.e., the water depth on the downstream face of the dam averages 23 cm). Water head is related to pressure or force on the dam, and is proportional to seepage through the dam [25–27]. For dams generally, whether man-made, formed by landslides, or beaver built, seepage refers to leakage of water through the dam or its foundation. Excessive seepage can cause internal erosion within the dam body or erosion of its foundations, which weakens the dam’s structure and can lead to failure. Seepage can be controlled by injecting grout (mud for beaver) into permeable areas to fill voids and reduce permeability, and by blanketing the dam face on its upstream side with impermeable material (mud for beaver dams). Consequently, high water head beaver dams likely have higher risk of failure than low-head dams and likely require more maintenance by beaver to reduce this risk. These risks are likely exacerbated by building on soft sediments typical of muddy to sandy tidal channels. Stair-stepping reduces the hydraulic head for an individual dam thereby reducing seepage, instability, and maintenance. A single large dam also leads to greater risk in catastrophic loss of beaver habitat in a channel compared to a series of dams which provides some redundancy that results in only partial loss of beaver habitat with a single dam failure. The nearly 2-m high dam that was observed, was only 24 m distant from a beaver lodge, the proximity perhaps facilitating frequent dam maintenance.

### Habitat restoration

Puget Sound Chinook salmon and Oregon Coast coho salmon (*O. kisutch*) are threatened species under the Endangered Species Act. Because juveniles of both species benefit significantly from rearing in tidal marsh habitats [28], there has been considerable investment in the region for tidal marsh habitat restoration to recover these species. With the recognition that beaver are a natural part of tidal wetlands in the Pacific Northwest, and likely elsewhere, and given their status as ecosystem engineers, their role in ecosystem restoration and recovery should be investigated [8]. Tidal marsh restoration can potentially benefit beaver as well as juvenile salmon and other fish and wildlife, as long as the habitat needs of beaver are considered, i.e., sufficient supply of freshwater, tidal channels, shrubs, trees, and herbaceous food plants. Beaver colonization of the Milltown Island restoration site in the South Fork Skagit Delta shows that restoration sites can support beaver, though the lower density of beaver dams and lodges on the site, compared to reference marsh conditions, suggests thoughtful consideration of beaver habitat needs could improve beaver responses to restoration projects. Beaver occupancy of habitat restoration sites could in turn benefit juvenile salmon rearing on the site [8].

Further research on how beaver adapt to tidal marsh conditions and how their dams and low-tide pools interact with other biota and eco-geomorphic processes is necessary before rashly rushing to translocate beaver to tidal marsh systems. It is important, for example, to understand what the beaver carrying capacity of different types of tidal marshes might be, and whether beaver are already pushing those limits in any given location. Where do dispersing estuarine juveniles go once they are ready to establish their own territories? Are they more likely to migrate along the coast as they search for territorial opportunities, or move inland? How do we account for beaver landscape connectivity in the course of tidal marsh restoration so that juvenile beaver have opportunities for dispersal?

Tidal marsh restoration planners and engineers in the Pacific Northwest (e.g., the Washington Department of Fish and Wildlife and the Columbia River Estuary Task Force) have shown interest in constructing beaver dam analogs (BDAs [29,30]) in tidal channels. However, there are no design guidelines for BDAs in tidal systems (though this paper provides data that could be used for this purpose), and it is unclear whether they are appropriate for tidal systems. In fluvial systems, BDAs are typically installed in incised streams to trap sediment and raise the stream bed to restore floodplain connectivity. The geomorphic role of tidal beaver dams is still unknown, and their ecological role still needs better resolution, so it seems premature to incautiously adapt BDAs to tidal channels. Uncritically applying fluvial paradigms to tidal systems is likely unwise given the many differences between fluvial and tidal systems, particularly bidirectional flow, salinity stratification, and twice-daily fluctuations of 1–5 m in water surface elevation in tidal systems. There is a great deal of tidal beaver natural history that needs to be understood before we can wisely proceed with beaver reintroductions or BDA installations in tidal marsh restoration sites.

## Summary

In the Pacific Northwest, at least, beaver are not unusual in tidal wetlands that contain or are bordered by woody vegetation. Tidal beaver are widespread and occur in a wide variety of coastal settings, reflecting the adaptability of beaver and the likelihood that they are important ecosystem engineers in estuarine as well as riverine systems.

In tidal systems, beaver build at least two types of dams. The most common type of dam is located in tidal channels and is composed of sticks and mud, sometimes incorporating bank slumps and sometimes overgrown with herbaceous wetland vegetation, such as sedge. Typically, these dams range in size from 1 to 4 m wide and from 20 to 100 cm tall, though larger dams are sometimes encountered. The dams typically stair-step low tide water levels up to the marsh surface providing extensive areas of low-tide refuge for beaver and other tidal channel denizens. During high tides most, if not all, of the beaver dams are completely inundated so that movement by tidal channel fauna, such as fish, is unimpeded. The dams are therefor functional mostly at low tide, serving to allow beaver movement for foraging and to permanently inundate lodge entrances.

The other kind of dam is much less common. It is located on marsh surfaces, oriented perpendicularly to a tidal channel, and includes a dam within the channel. This surficial dam ranges from 0.5 to 1.0 m in height, but can be several hundred meters long to produce large ponds on the marsh surface. These dams are found principally in small coastal systems with limited freshwater inflow from small streams. The function of the dams appears to be to trap the freshwater and augment the amount of freshwater habitat available to the resident beaver.

The widespread occurrence of beaver in low-salinity tidal wetlands that allow easy access to herbaceous and woody vegetation for forage and building materials suggests that this habitat is perfectly suitable for beaver, rather than sub-optimal compared to better-documented fluvial beaver systems. It also suggests that neglecting the role of beaver in coastal wetlands will lead to an incomplete understanding of these systems and deficient management, including for habitat restoration and protection.

## Supporting information

S1 File Tables S1-S2; Figs S1-S38. (DOCX)
