# Supplementary material for: Beaver in tidal habitat: Examples from the Pacific Northwest
Source: PLoS One. 2026 Jul 8;21(7):e0349313. doi: 10.1371/journal.pone.0349313 (PMC13345279; doi:10.1371/journal.pone.0349313)
Supplement: S1 File — (DOCX) [file pone.0349313.s001.docx]

Supporting Information for

“Beaver in Estuarine Tidal Habitat: Examples from the Pacific Northwest”

W. Gregory Hood

Skagit River System Cooperative

P.O. Box 368

La Conner, WA

ghood@skagitcoop.org

**Table S1**. Summary of the literature on statistics for riverine and lacustrine dam and lodge density.

| **Dams per km of channel** | **Reference** | **Colonies per km of fluvial channel or lacustrine shoreline** | **Reference** |
| --- | --- | --- | --- |
| 8.6^a^ | [1] | 0.83 | [11] |
| 16.0^b^ | [1] | 0.90 | [11] quoting [12] |
| 2.5^c^ | [2] | 1.25 | [11] quoting [13] |
| 0.14 | [3] | 0.74 | [14] |
| 14.3 | [4] | 0.48 | [15] |
| 1.2 | [5] | 0.62 | [15] quoting [16] |
| 25.0 | [6] | 0.33 | [15] quoting [17] |
| 2.6 | [7] | 1.00 | [15] quoting [18] |
| 3.0 | [8] | 0.39 | [15] quoting [19] |
| 15.8^d^ | [9] | 0.83 | [15] quoting [20] |
| 5.1^e^ | [9] | 0.74 | [21] |
| 5.9 | [10] | 0.32 | [22] |
|  |  | 0.35 | [23] |
|  |  | 0.63 | [24] quoting [25] |
|  |  | 0.78 | [26] |
|  |  | 0.76 | [27] |
|  |  | 0.22 | [28] |
|  |  | 0.19 | [29] |
|  |  | 0.30 | [30] |
| **Mean = 7.8** |  | **Mean = 0.61** |  |

^a^ Cran Carré; ^b^ Ross Creek; ^c^ Minnesota; ^d^ Coyote Creek; ^e^ Cimmaron Canyon

**Table S2**. Summary of the literature on statistics for riverine beaver dam and pool metrics.

| **Reference** | ***N*** | **Water head (cm)** | **Dam height (cm)** | **Pool depth (cm)** | **Pool area (m^2^)** |
| --- | --- | --- | --- | --- | --- |
| [31] | 44 |  | 70 |  |  |
| [26] | 16^b^ |  | 67^b^ |  |  |
| [26] | 16^c^ |  | 47^c^ |  |  |
| [27] | 128 |  | 74 |  |  |
| [32] |  |  |  | 80 | 120 |
| [33] | 325 |  | 94 |  |  |
| [34] | 21 |  | 99 |  |  |
| [35] | 40 |  | 90 |  |  |
| [36] | 10 |  | 100 |  |  |
| [20] | 14 |  | 55 |  |  |
| [37] | 10 |  | 94 |  |  |
| [38] | 29 |  | 61 |  |  |
| [39] | 51 |  | ~65 |  |  |
| [40] | 77 | 42 |  |  |  |
| [41] | 1208^e^ |  |  |  | 168^e^ |
| [41] | 161^f^ |  |  |  | 311^f^ |
| [41] | 35^g^ |  |  |  | 158^g^ |
| [21] | 39 | 22 | 15 | 55 |  |
| **Fluvial weighted mean^a^** |  | **32** | **82.3** | **68** | **184** |

^a^ Weighted by sample size

**References for Tables S1 and S2**

1. Naiman RJ, Melillo JM, Hobbie JE. Ecosystem alteration of boreal forest streams by beaver (castor canadensis). Ecology. 1986; 67:1254–1269.
2. Naiman RJ, Johnston CA, Kelley JC. Alteration of North American streams by beaver. BioScience. 1988; 38:753–762.
3. McComb WC, Sedell JR, Buchholz TD. Dam-site selection by beavers in an eastern Oregon basin. Great Basin Nat. 1990; 50(3):273-281.
4. Woo M, Waddington JM. Effects of beaver dams on sub-arctic wetland hydrology. Arctic. 1990; 43:223-230. doi:10.14430/arctic1615
5. Leidholt-Bruner K, Hibbs DE, McComb, WC. Beaver dam locations and their effects on distribution and abundance of coho salmon fry in two coastal Oregon streams. Northwest Sci. 1992; 66(4):218-223.
6. Butler DR, Malanson GP. Beaver landforms. Can Geogr. 1994; 38:76-79.
7. Suzuki N, McComb WC. Habitat classification models for beaver (*Castor canadensis*) in the streams of the central Oregon Coast Range. Northwest Sci. 1998; 72:102–110.
8. MacCracken JG, Lebovitz AD. Selection of in-stream wood structures by beaver in the Bear River, southwest Washington. Northwestern Nat. 2005; 86(2):49-58.
9. Cavin RM. Beaver Dam dimensions and distribution in northeastern New Mexico. Beaver dam dimensions and distribution in northeastern New Mexico [thesis]. Texas State University; 2015.
10. Gusarov AV, Sharifullin AG, Beylich AA, Lisetskii FN. Features of the distribution of beaver dams and ponds along small rivers: The Volga-Kama region, European Russia. Hydrology. 2024; 11:53.
11. Howard RJ, Larson JS. A stream habitat classification system for beaver. J Wildl Manage. 1985; 49:19-25.
12. Collins TC. Population characteristics and habitat relationships of beaver, Castor canadensis, in northwest Wyoming [dissertation]. Laramie (WY): University of Wyoming; 1976.
13. Nordstrom WR. Comparison of trapped and untrapped beaver populations in New Brunswick [thesis]. Frederickton (NB): University of New Brunswick; 1972.
14. Beier P, Barrett RH. Beaver habitat use and impact in Truckee River Basin, California. J Wildl Manage. 1987; 51:794-799.
15. Cotton FE. Potential beaver colony density in parts of Québec [dissertation]. Blacksburg (VA): Virginia Polytechnic Institute and State University; 1990.
16. Payne NF. Trapline management and population biology of Newfoundland beaver [dissertation]. Logan (UT): Utah State University; 1975.
17. Lawrence WH. Michigan beaver populations as influenced by fire and logging [dissertation]. Ann Arbor (MI): University of Michigan; 1954.
18. Northcott THA. An investigation of the factors affecting carrying capacity of selected areas in Newfoundland for the beaver, Castor canadensis caecator Bangs, 1913 [thesis]. St-John's (NF): Memorial University; 1964.
19. Novakowski NS. Population dynamics of beaver population in northern latitudes [dissertation]. Saskatoon (SK): University of Saskatchewan; 1965.
20. Howard RJ. Beaver habitat classification in Massachusetts [thesis]. Amherst (MA): University of Massachusetts; 1982.
21. Robel RJ, Fox LB. Comparison of aerial and ground survey techniques to determine beaver colony densities in Kansas. Southwest Nat. 1993; 1:357-361.
22. McCall TC, Hodgman TP, Diefenbach DR, Owen RB. Beaver populations and their relation to wetland habitat and breeding waterfowl in Maine. Wetlands. 1996; 16(2):163-172.
23. Smith DW. Beaver Survey: Yellowstone National Park. Yellowstone National Park, Mammoth Hot Springs, Wyoming; 1998. Report YCR-NR-99-3.
24. Gurnell AM. The hydrogeomorphological effects of beaver dam-building activity. Progr Phys Geogr. 1998; 22(2):167-189.
25. Boyce MS. Habitat ecology of an unexploited population of beavers in interior Alaska. In: Chapman JA, Pursley D. editors. Worldwide furbearer conference proceedings. Falls Chard, Virginia: Donnelly; 1983. p. 155-186.
26. Murphy SC, Smith DW. Documenting trends in Yellowstone’s beaver population: A comparison of aerial and ground surveys in the Yellowstone Lake Basin. In: Anderson RJ, Harmon D. editors. Yellowstone Lake: Hotbed of chaos or reservoir of resilience. Yellowstone National Park (WY): Yellowstone Center for Resources; 2002. p.172-178.
27. Rosell F, Bergan F, Parker H. Scent-marking in the Eurasian beaver (Castor fiber) as a means of territory defense. J Chem Ecol. 1998; 24(2):207-219.
28. Ribic CA, Donner DM, Beck AJ, Rugg DJ, Reinecke S, Eklund D. Beaver colony density trends on the Chequamegon-Nicolet National Forest, 1987-2013. PLoS ONE. 2017; 12(1): e0170099. doi:10.1371/journal.pone.0170099.
29. Westbrook CJ, Waldner Z, Dzus E, Dzus C, Stoll NL. Beavers in the boreal: A lodge census of Besnard Lake. Blue Jay. 2022; 80(2):6-12.
30. Rosell F, Lodberg-Holm HK, Meijer F, Midbøe M. A home for the many? Beaver Lodges as hotspots for bird and mammal diversity. Sci Total Environ. 2025; 990: 179898. doi:10.1016/j.scitotenv.2025.179898.
31. Beedle DL. Physical dimensions and hydrologic effects of beaver ponds on Kuiu Island in southeast Alaska [thesis]. Corvallis (OR): Oregon State University; 1991.
32. Demmer R, Beschta RL. Recent history (1988–2004) of beaver dams along Bridge Creek in central Oregon. Northwest Sci. 2008; 82(4):309-318.
33. Hafen KC, Wheaton JM, Roper BB, Bailey P, Bouwes N. Influence of topographic, geomorphic, and hydrologic variables on beaver dam height and persistence in the intermountain western United States. Earth Surf Process Landf. 2020; 45:2664-2674.
34. Lokteff RL, Roper BB, Wheaton JM. Do beaver dams impede the movement of trout? Trans Am Fish Soc. 2013; 142(4):1114-1125.
35. Karran DJ, Westbrook CJ, Wheaton JM, Johnston CA, Bedard-Haughn A. Rapid surface-water volume estimations in beaver ponds. Hydrol Earth Syst Sci. 2017; 21(2):1039-1050.
36. Majerova M, Neilson BT, Schmadel NM, Wheaton JM, Snow CJ. Impacts of beaver dams on hydrologic and temperature regimes in a mountain stream. Hydrol Earth Syst Sci. 2015; 19(8):3541-3556.
37. Meentemeyer RK, Butler DR. Hydrogeomorphic effects of beaver dams in Glacier National Park, Montana. Phys Geogr. 1999; 20(5):436-446.
38. Nagle S. Dam dimensions and surface porosity affect the water storage capacity of beaver dam analogs compared to natural Beaver dams [thesis]. Ellensburg (WA): Eastern Washington University; 2024.
39. Neumayer M, Teschemacher S, Schloemer S, Zahner V, Rieger W. Hydraulic modeling of beaver dams and evaluation of their impacts on flood events. Water. 2020; 12:300. doi:10.3390/w12010300.
40. Ronnquist AL. Dam different! How the physical properties of beaver dams influence water storage dynamics [thesis]. Saskatoon (SK): University of Saskatchewan; 2021.
41. Wan L, Fairfax E, Maher K. Factors influencing surface water accumulation in beaver pond complexes across the Western United States. Commun Earth Environ. 2025; 6(1):614. https://doi.org/10.1038/s43247-025-02573-x.

*Diversity of tidal beaver dams*

This section of the Supporting Information intends to show the diversity of tidal beaver dams that can be encountered in tidal wetlands of the Skagit Delta and the Snohomish Estuary.


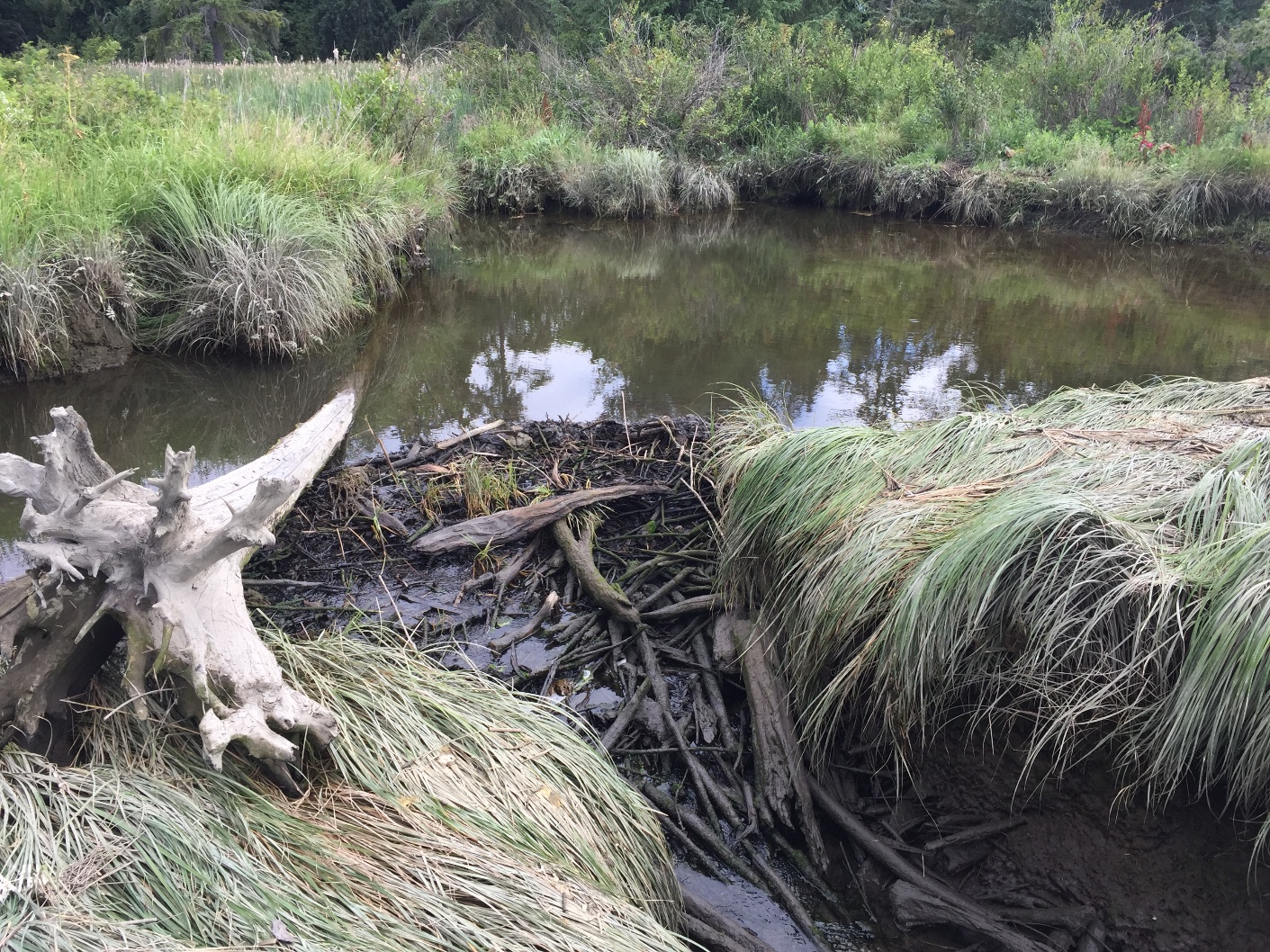


**Figure S1**. Beaver dam and pond in the Snohomish Estuary’s Quilceda marsh (48.05° N, 122.19° W). Note shrubs and spruce trees in background, tidal sedge (*Carex lyngbyei*) in the foreground. A log has drifted in on a higher high tide and has come to rest on the side of the beaver dam. The pond is waist to chest deep with an accumulation of soft, unconsolidated sediments. During higher high tides water reaches the marsh surface. Photo by the author.


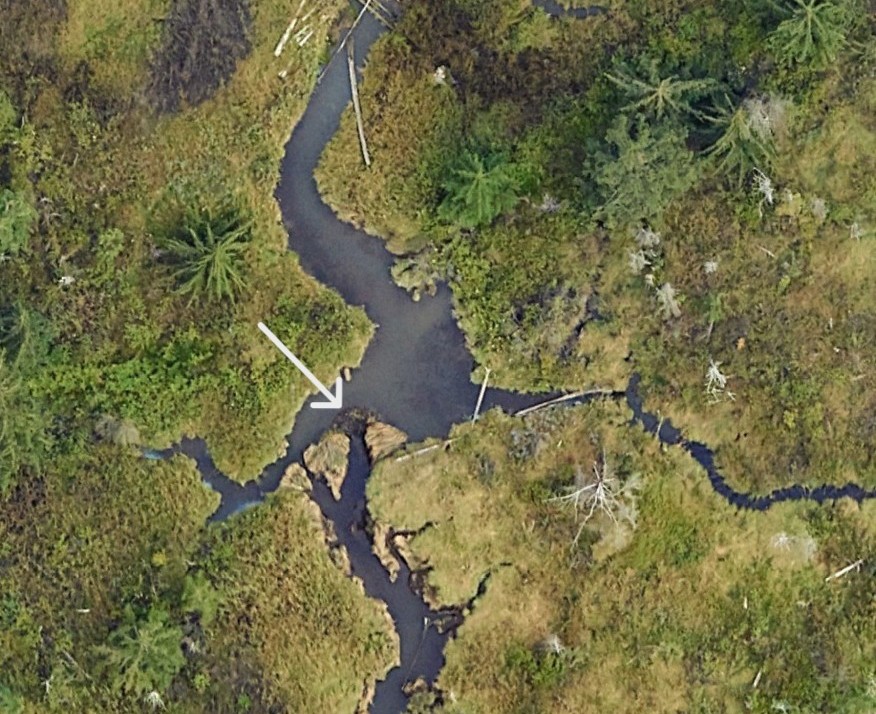


**Figure S2**. Google Earth view of the beaver dam in Fig. S1. Map Data © 2022 Google.


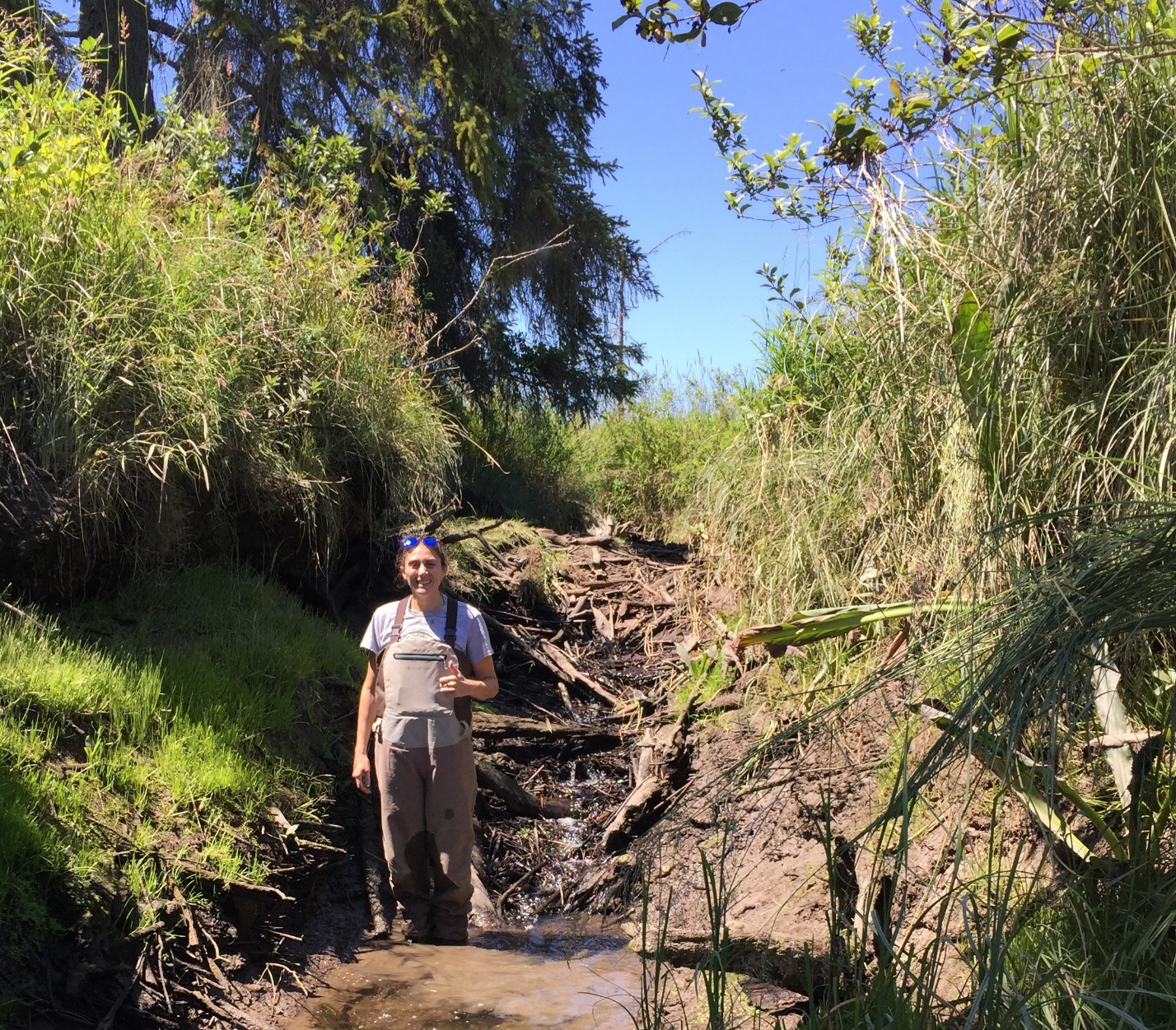


**Figure S3**. One of the tallest beaver dams that I have encountered; on the east side of Otter Island in the Snohomish Estuary (48.01° N, 122.14° W). Molly Alves (~170 cm), Tulalip Tribes biologist for scale. During higher high tides water is at least 1 m above the dam top. Photo by the author.


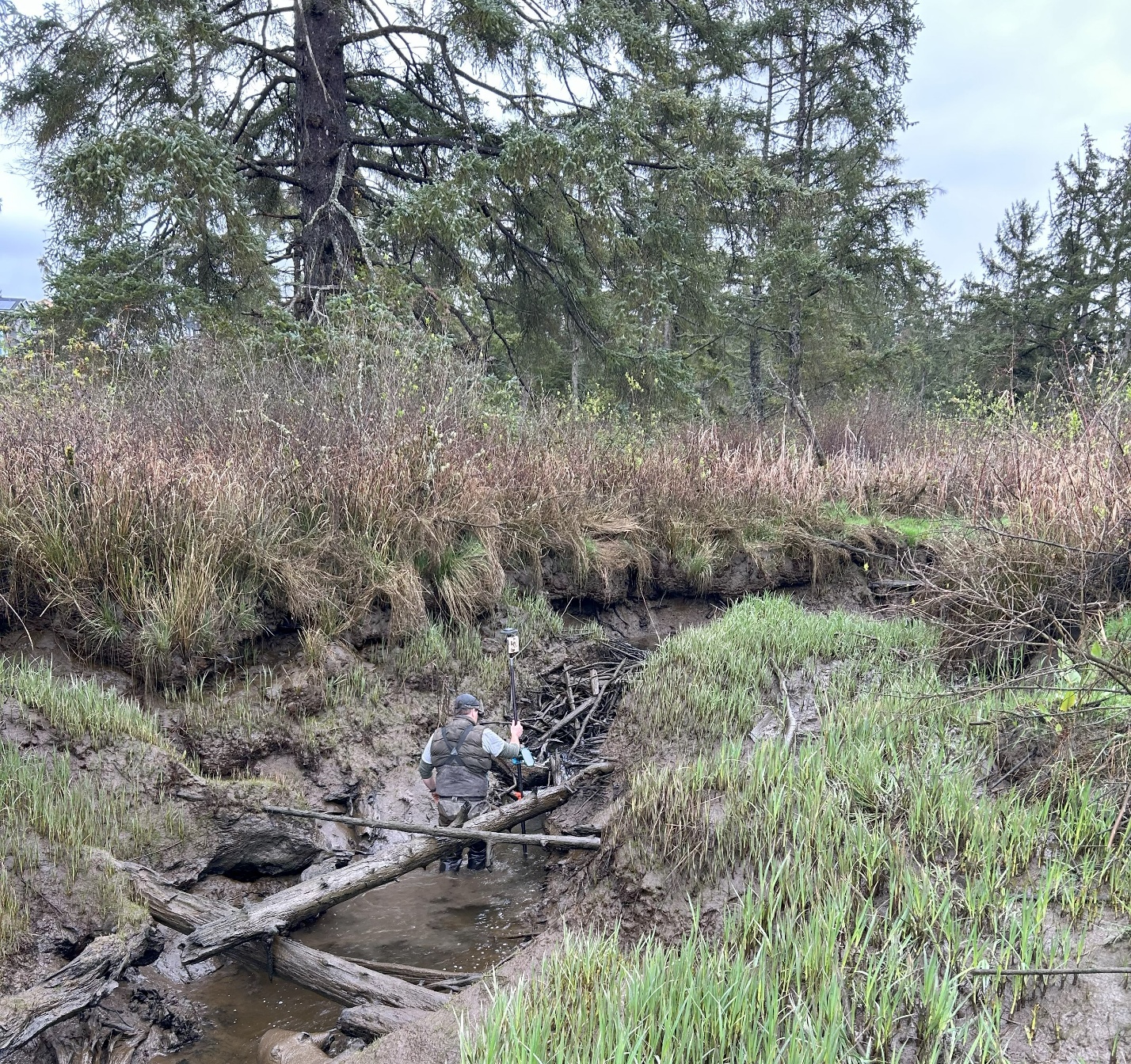


**Figure S4**. Another tall beaver dam, at Heron Point in the Snohomish Estuary (48.03° N, 122.155° W). Todd Zackey (180 cm), Tulalip Tribes biologist is surveying the channel profile with an RTK-GPS. Note tidal sedge in foreground, wild rose and other shrubs on the channel bank with Sitka spruce (*Picea sitchensis*) in the background. At higher high tide, water reaches the top of the bank. Photo by the author.


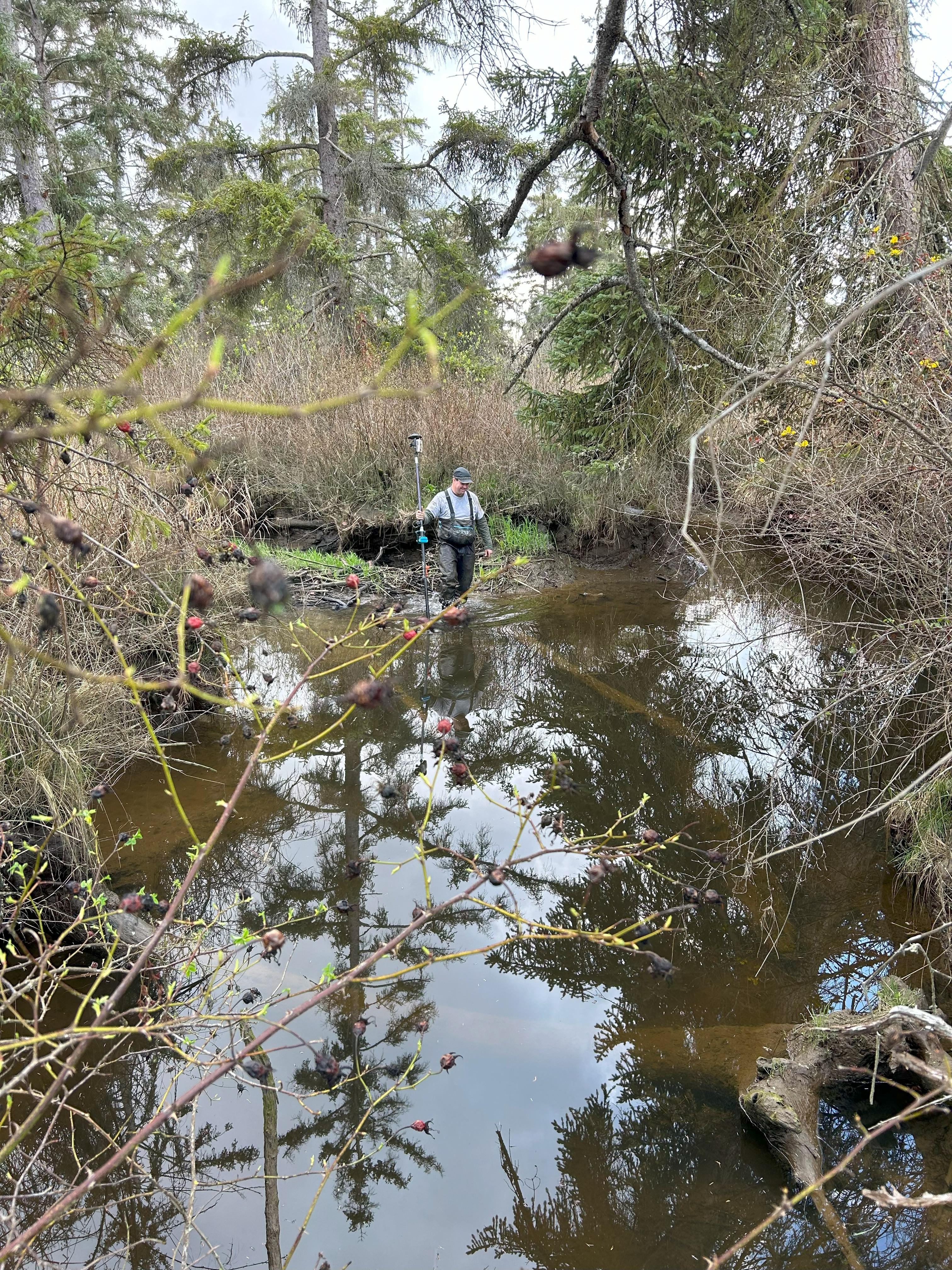


**Figure S5**. Beaver dam and pond at Heron Point in the Snohomish Estuary (48.03° N, 122.154° W). The dam is just behind Todd Zackey, Tulalip Tribes biologist who is surveying the channel profile with an RTK-GPS. Note Sitka spruce trees in background. During higher high tides water is at least 0.5 m higher. Photo by the author.


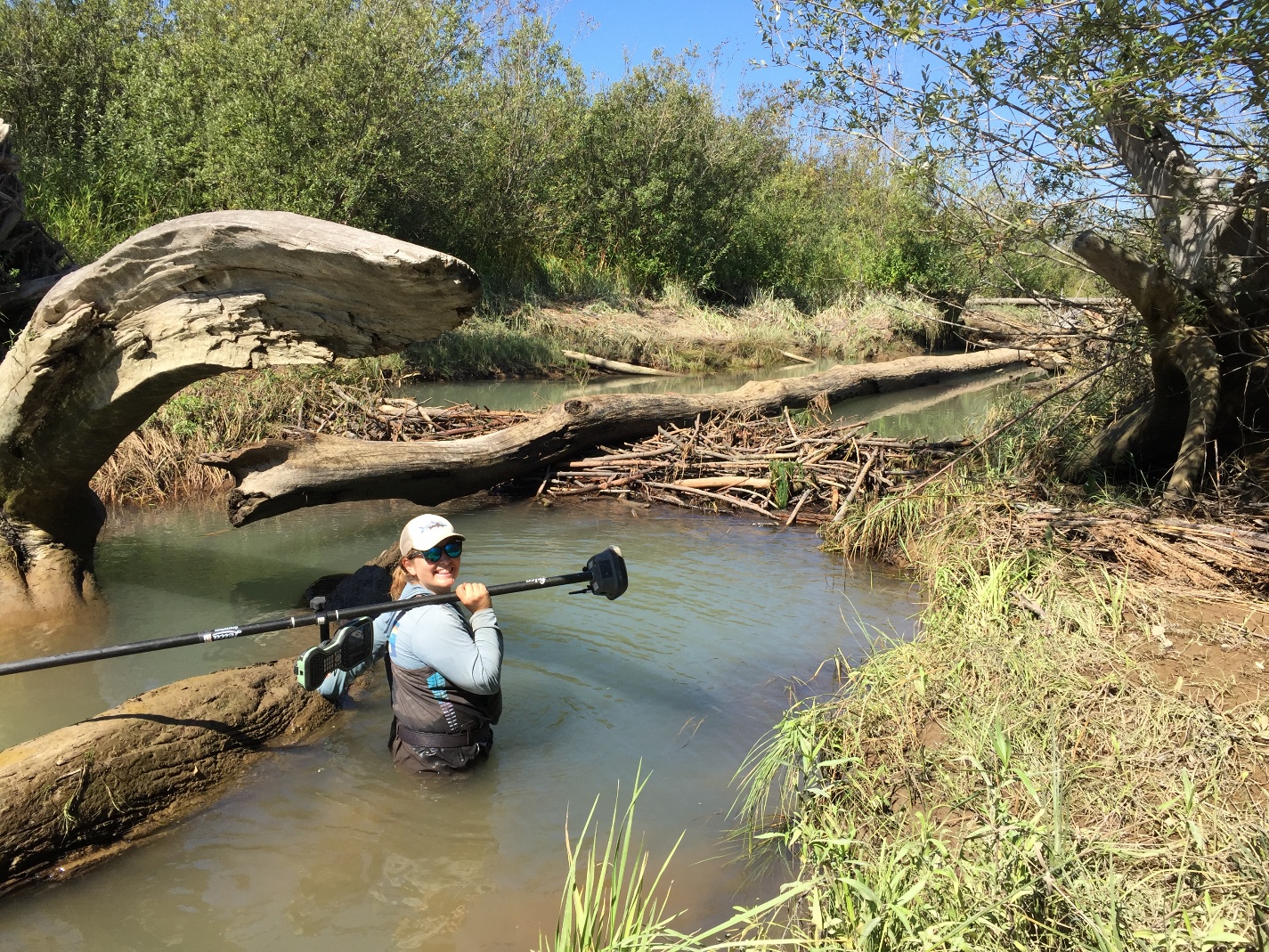


**Figure S6**. Beaver dam in the North Fork Skagit Delta (48.373° N, 122.494° W). Tidal sedge lines the bank, behind which are extensive willow (*Salix* spp.) thickets. The log lying on top of the dam floated in on the high tide; higher high tides flood the sedge banks by at least 0.5 m. Kathleen McKeegan, SRSC biologist, is wading through a beaver pond as she surveys the channel profile with an RTK-GPS. Photo by the author.


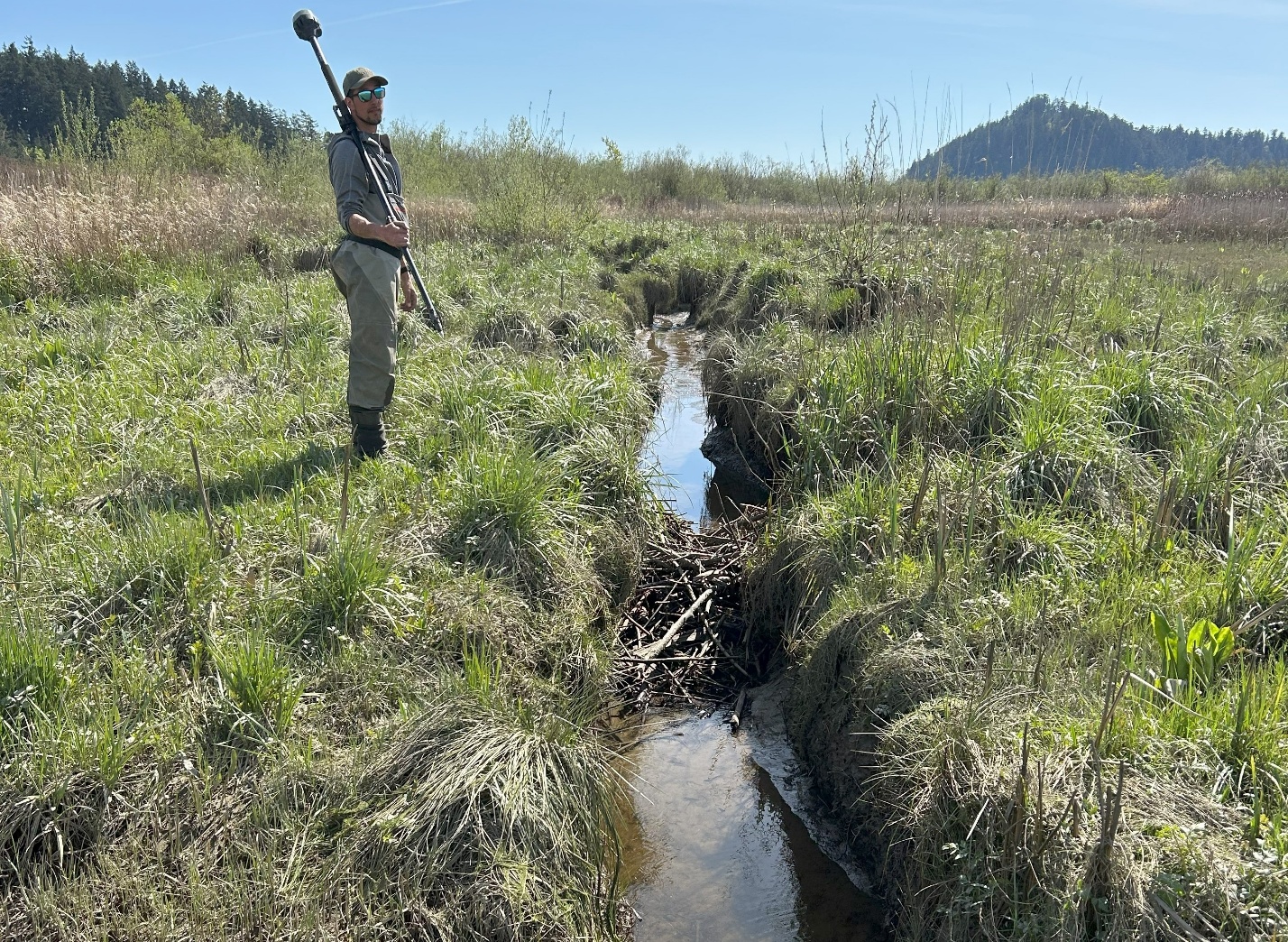


**Figure S7**. Beaver dam in a small tidal channel of the North Fork Skagit Delta (48.379° N, 122.490° W). Dennis Newman (180 cm), SRSC biologist with RTK-GPS, for scale. During higher high tides, the marsh surface is flooded by at least 0.5 m. Photo by the author.


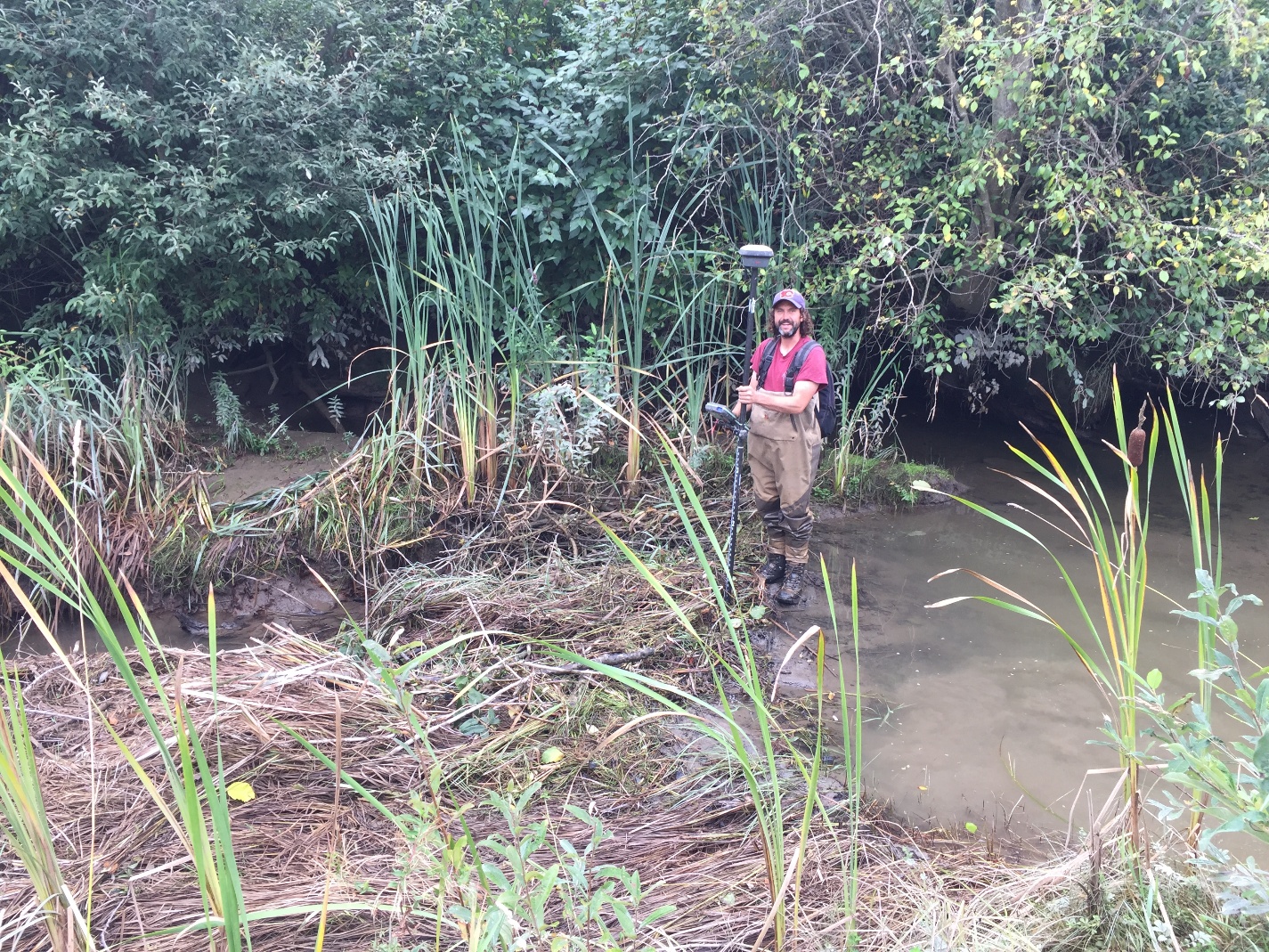


**Figure S8**. Beaver dam in a senescing North Fork Skagit distributary (48.364° N, 122.482° W). Neal Robertson (170 cm), SRSC biologist surveying the channel profile with an RTK-GPS, is standing on top of the beaver dam; note water head difference from right to left. Prior to avulsion of the mainstem North Fork channel, this distributary was a sandy-bottomed channel without any vegetation. The channel bank behind Neal is almost 2 m higher than the shoaling channel bottom and is flooded during higher high tides. Beaver have been building dams in the senescing distributaries over the last five years, expanding their low-tide range of movement. Photo by the author.


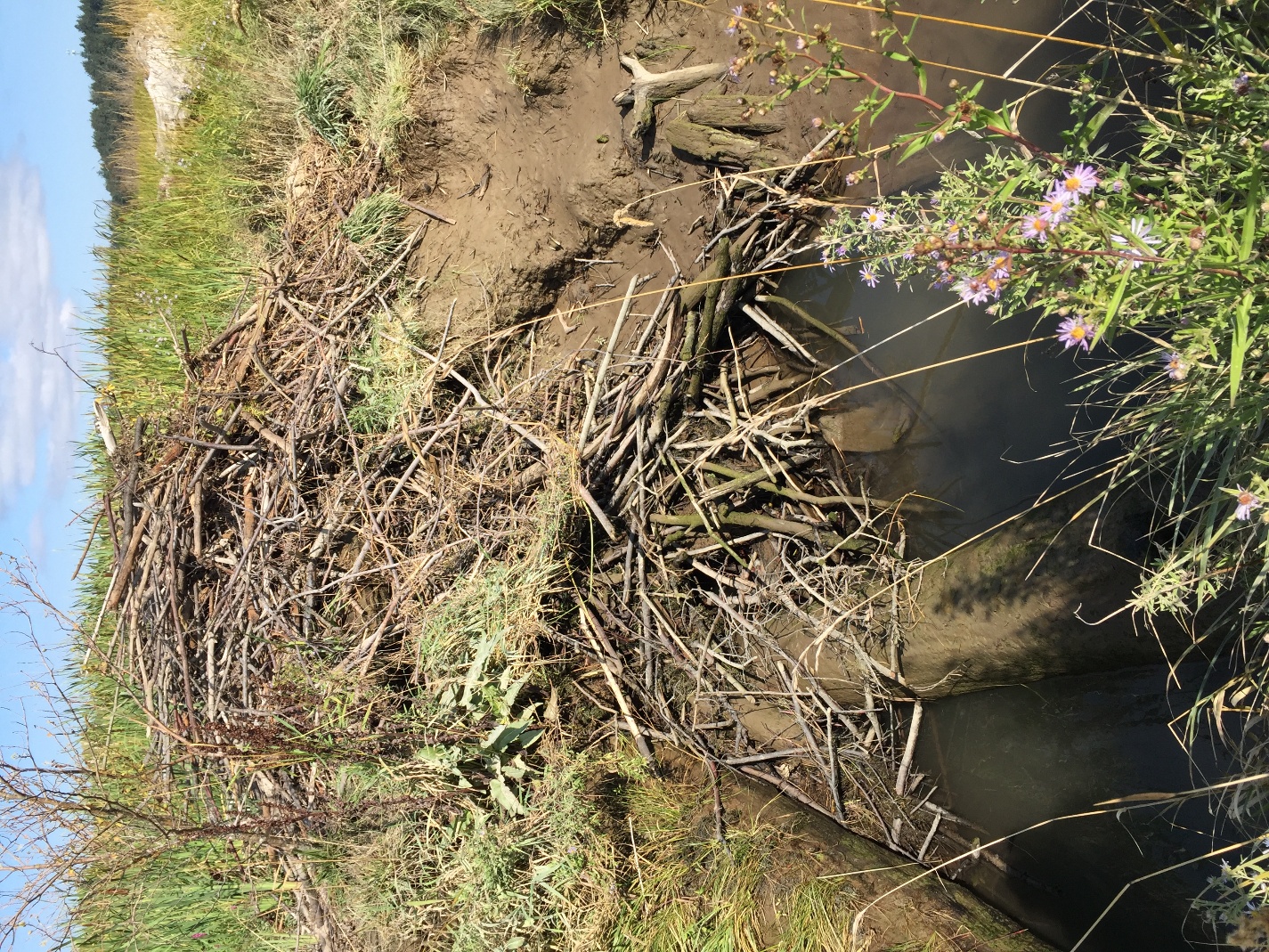


**Figure S9**. Beaver lodge at lower low tide in the North Fork Skagit Delta (48.373° N, 122.499° W). During higher high tides the marsh surface is flooded by at least 0.5 m of water. Only one dam was downstream of the lodge, so low tide water is relatively low compared to the lodge entrance. Photo by the author.

*Beaver channel profiles*

This section intends to show additional representative profiles of tidal channels with beaver dams beyond those shown in the main paper.


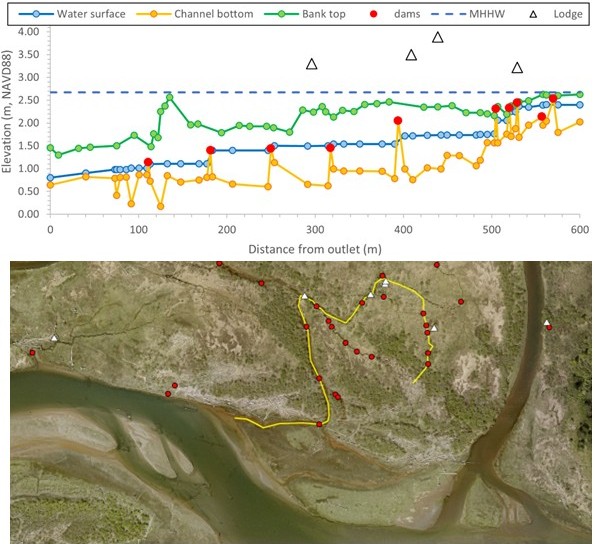


**Figure S10**. [Top frame] Beaver channel profile at low tide in the North Fork Skagit tidal marshes. MHHW = mean higher high water. Note the presence of five lodges along this channel. Also, the first 105 m of the channel cut through newly prograding marsh developing in the reach of the North Fork Skagit River that is downstream from the recent (beginning in 2004) avulsion. [Bottom frame] Location of channel profile (yellow line). Sandbars are developing in this senescing reach, and they are being colonized by marsh vegetation. Beaver are often damming channels in these prograding marshes as their elevations increase. Vegetation is primarily tidal sedge (*Carex lyngbyei*), some non-native cattail (*Typha angustifolia*) and willows (*Salix* spp.). Imagery © 2023 Eagleview.


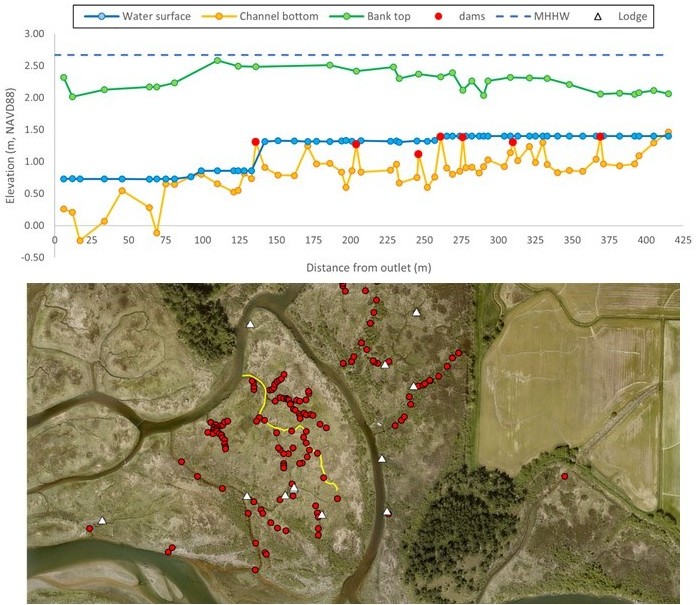


**Figure S11**. [Top frame] Beaver channel profile at low tide in North Fork Skagit tidal marshes. MHHW = mean higher high water. [Bottom frame] Location of channel profile (yellow line). Vegetation is primarily tidal sedge (*Carex lyngbyei*), invasive reed canarygrass (*Phalaris arundinacea*), some non-native cattail (*Typha angustifolia*) and willows (*Salix* spp.). Imagery © 2023 Eagleview.


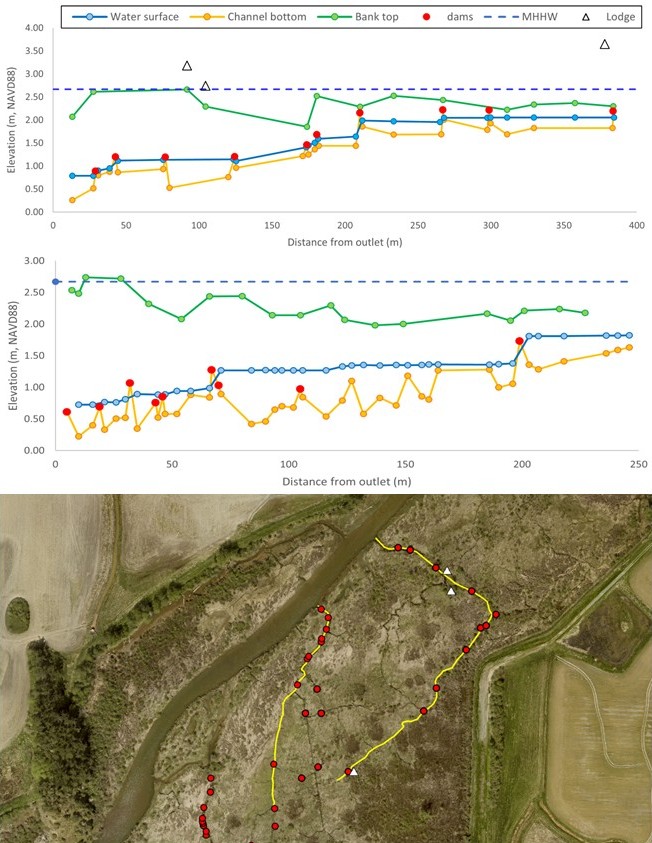


**Figure S12**. [Top frame] Beaver channel profiles at low tide in the Sullivan Slough area of the North Fork Skagit marshes. MHHW = mean higher high water. [Bottom frame] Location of channel profiles (yellow lines). The channel on the left corresponds to the bottom graph in the top frame; the channel on the right corresponds to top graph in the top frame. Note that the lowest lodge was only partially constructed. Vegetation is primarily tidal sedge (*Carex lyngbyei*), invasive reed canarygrass (*Phalaris arundinacea*), some non-native cattail (*Typha angustifolia*) and willows (*Salix* spp.). Imagery © 2023 Eagleview.


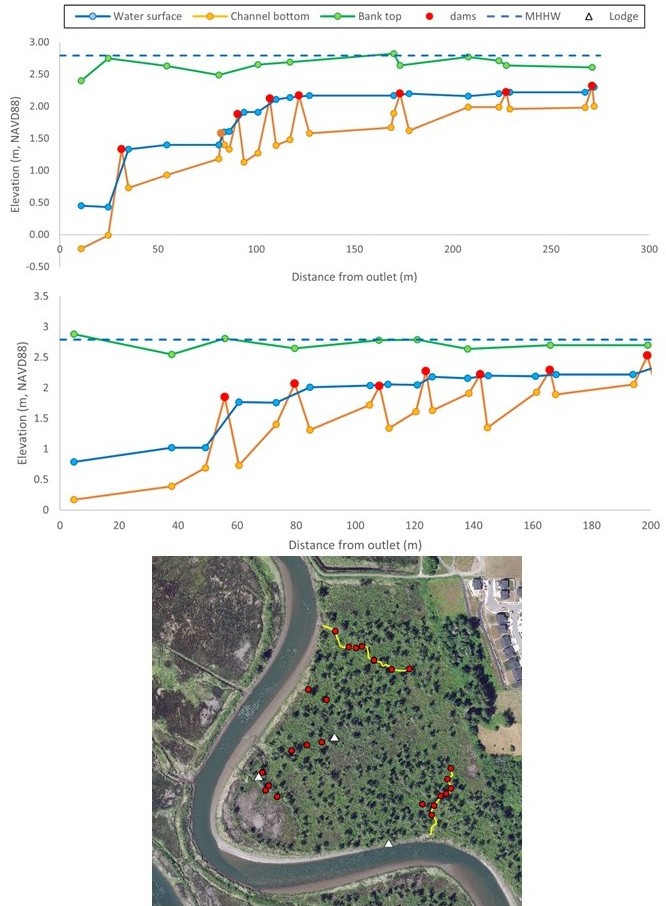


**Figure S13**. [Top frame] Beaver channel profiles at low tide at Heron Point in the Snohomish Estuary. MHHW = mean higher high water. [Bottom frame] Location of channel profiles (yellow lines). The channel on the top corresponds to the top graph in the top frame; the channel on the bottom corresponds to bottom graph in the top frame. Heron Point consists of a Sitka spruce tidal swamp. Map Data © 2020 Google.

*Regional distribution of tidal beaver*

This section of the Supporting Information intends to show the wide distribution of tidal beaver dams in river deltas, estuaries, and other coastal habitats of the Pacific Northwest, from southern British Columbia to midway along the Oregon Coast. Photo signatures of tidal beaver dams were identified in Google Earth aerial photographs. Those that were accessible without a boat, and those that did not require land-owner permission, were ground-truthed.


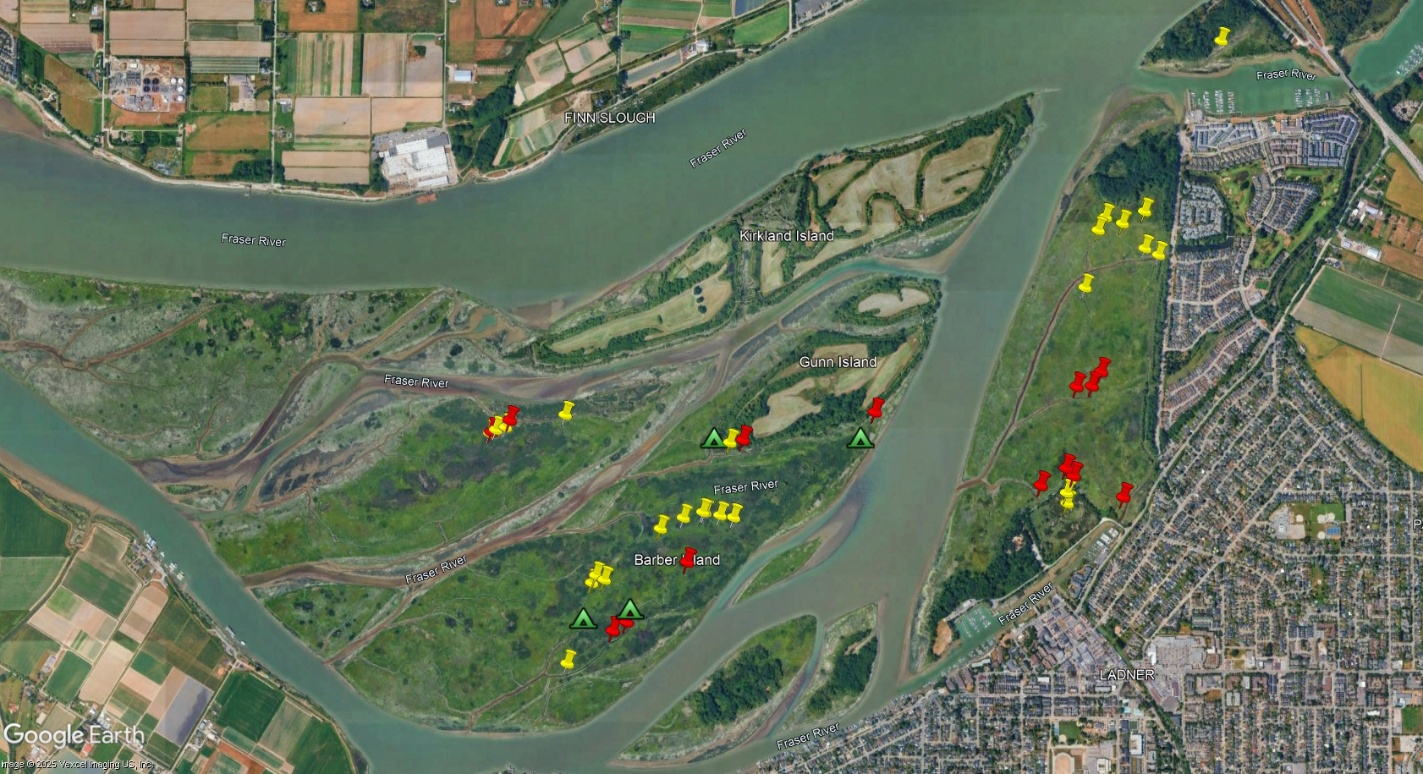


**Figure S14**. Fraser Delta (near Ladner, British Columbia) beaver dams interpreted from Google Earth aerial photographs: yellow = not ground truthed; red = ground truthed and confirmed; green “triangles” = confirmed beaver lodges. Dams and lodges in areas requiring boat access were confirmed by Eric Balke, Ducks Unlimited Canada. Map Data: Google, © 2026 Vexcel Imaging US, Inc.


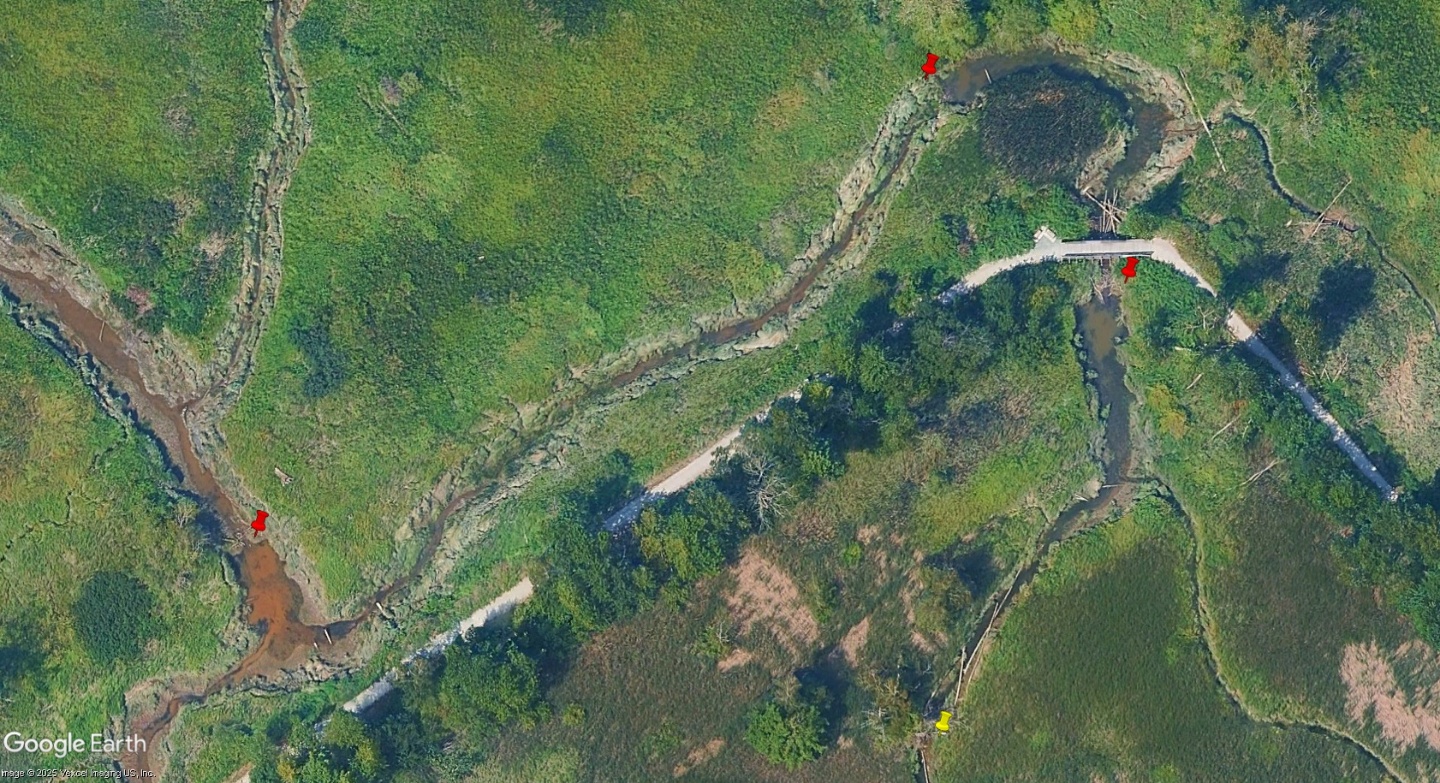


**Figure S15**. Beaver (red = ground-truthed and confirmed) dams near Ladner Harbor Park. The left-most dam is breached and holds little water. The right-most dam is located adjacent to a pedestrian bridge over a tidal channel. Map Data: Google, © 2026 Vexcel Imaging US, Inc.


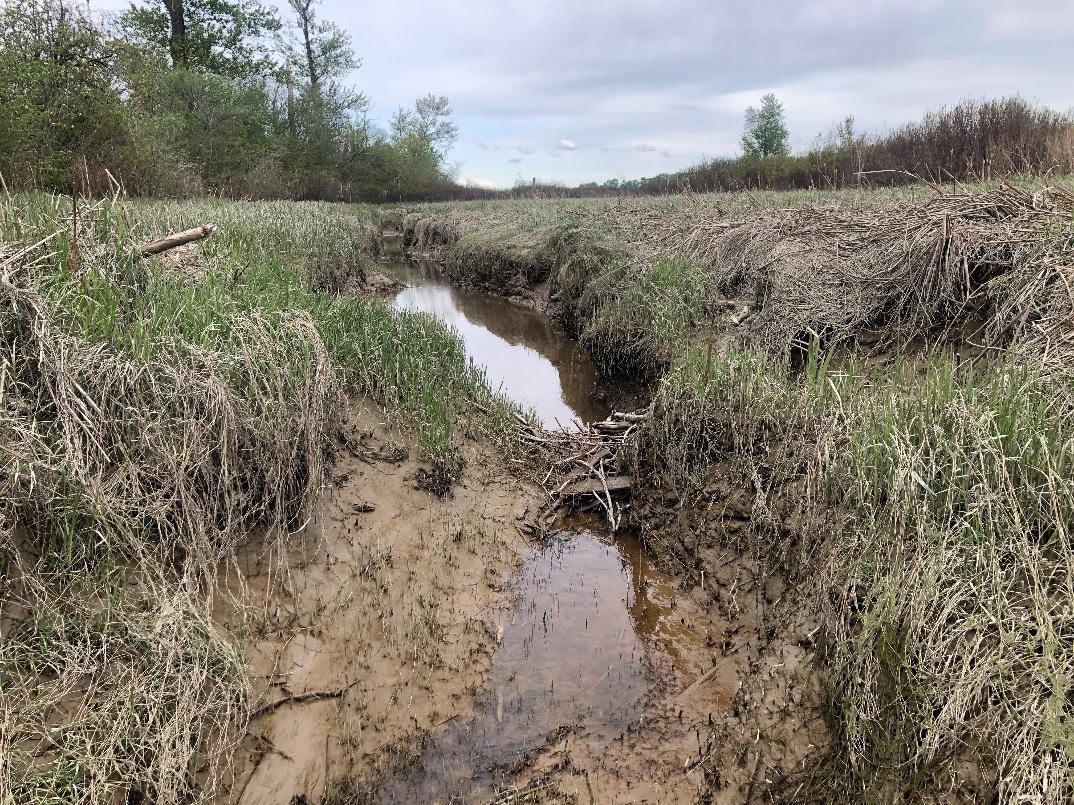


**Figure S16**. Beaver dam at low tide on Barber Island in the Fraser Delta (British Columbia). Vegetation consists of intertidal sedge, sweetgale (*Myrica gale*) thickets on the right, and mixed shrubs and spruce on the left. During higher high tides the marsh surface is flooded. Photo courtesy of Eric Balke, Ducks Unlimited Canada.


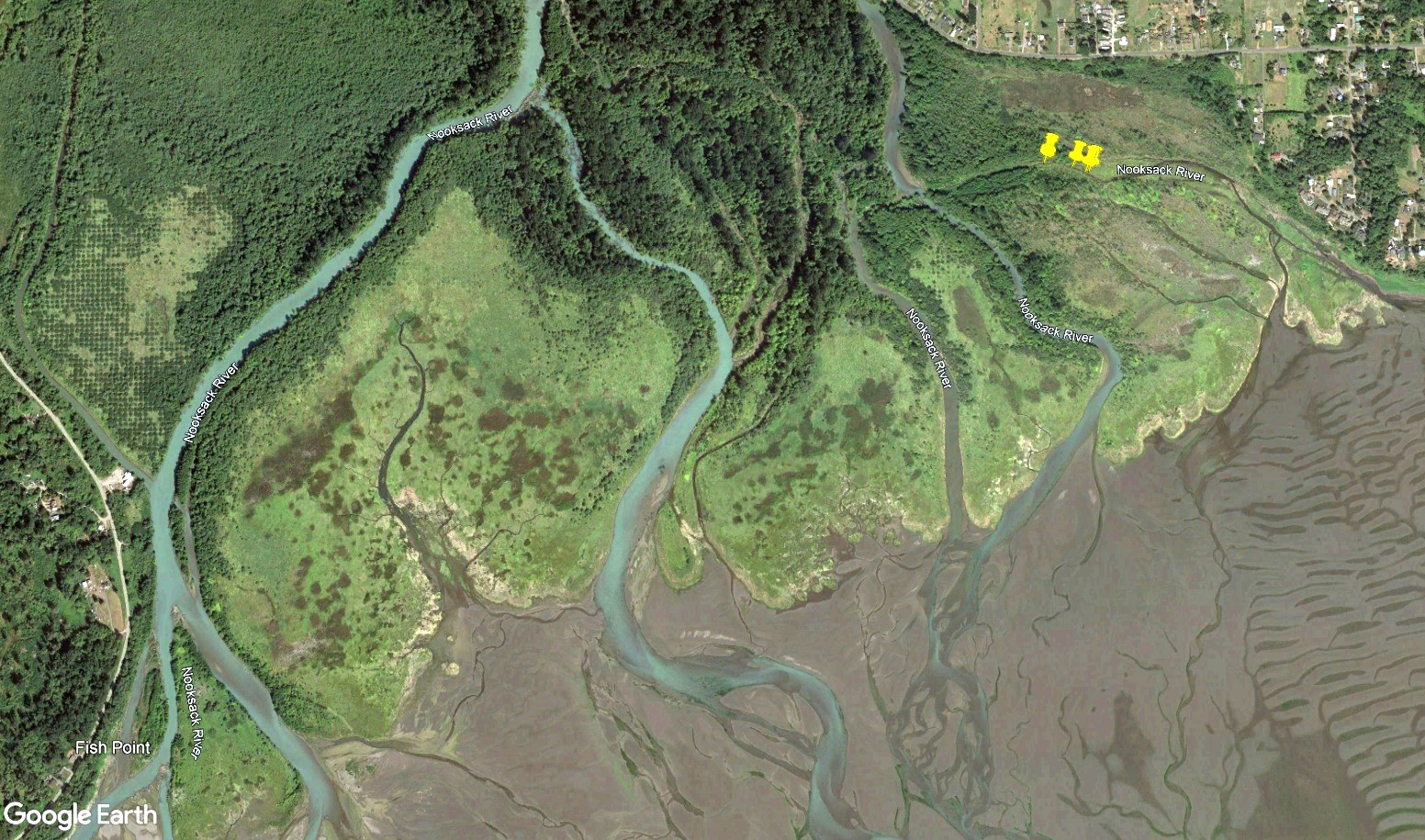


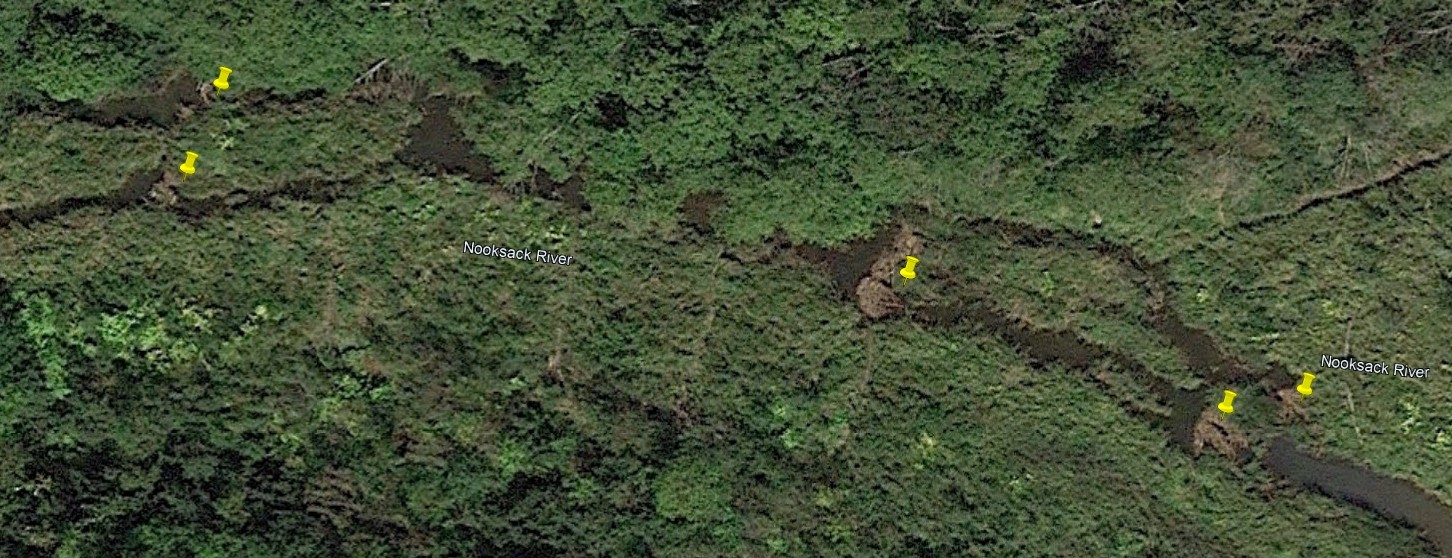


**Figure S17**. Beaver dam locations (yellow pins) in the Nooksack Delta, interpreted from Google Earth aerial photographs. These locations have not been ground-truthed. The beaver dams are located in a senescing delta distributary. The bottom frame shows detail (center of image is at 48.780° N, 122.571° W); Map Data © 2020 Google. Other beaver dams are likely present in the delta but are obscured by shrub and tree canopy.


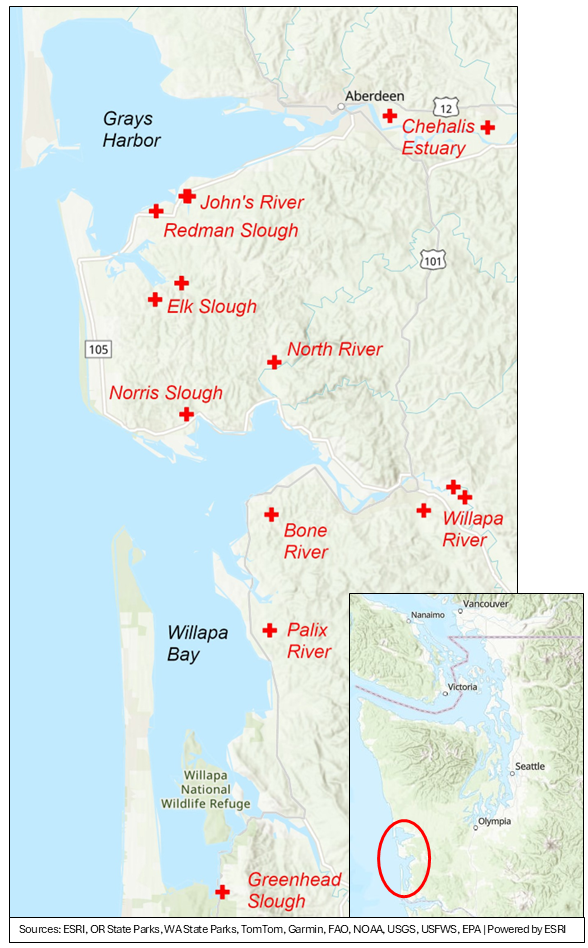


**Figure S18**. Vicinity map of coastal Washington ground-truth sites.


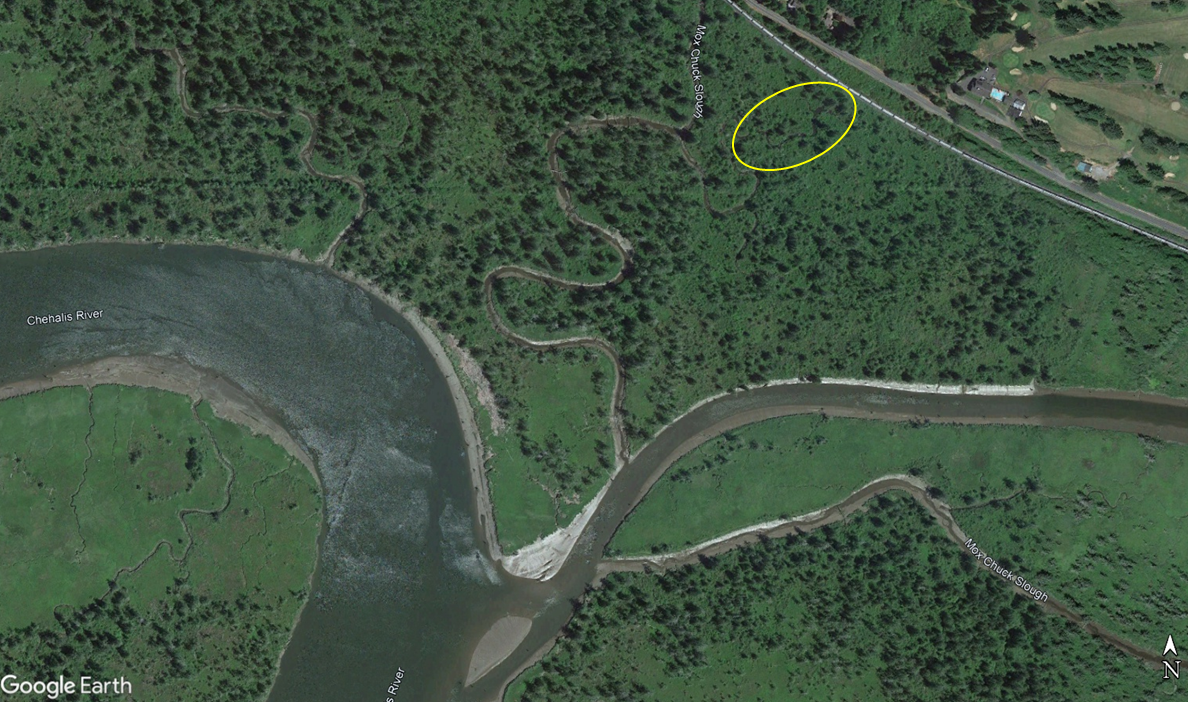


**Figure S19**. Mox Chuck Slough vicinity, showing the tidal floodplain of the Chehalis River, dominated largely by Sitka spruce trees, and shrubs such as *Lonicera involucrata* (black twinberry), *Spiraea douglasii* (hardhack), *Rosa nutkana* (wild rose), *Ribes sanguineum* (red flowering currant), and *Rubus spectabilis* (salmonberry). Ground-truthing of tidal beaver dams occurred in the area outlined by the yellow oval. Map Data © 2021 Google.


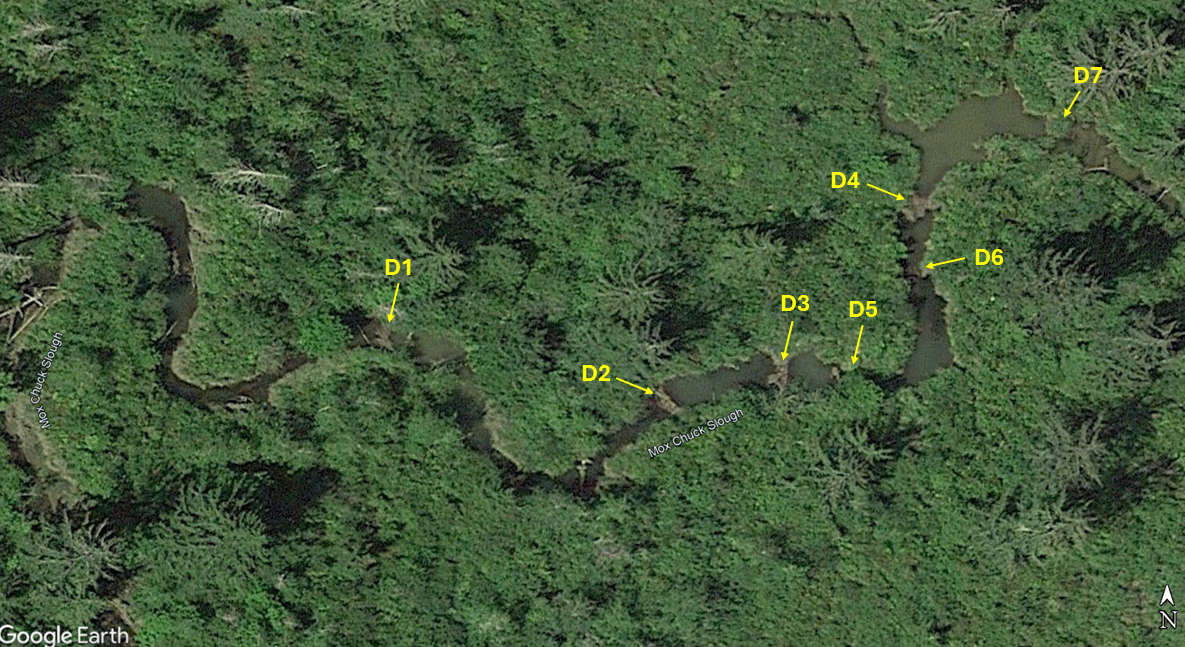


**Figure S20**. Ground-truthed Mox Chuck Slough dams; 1-4 were predicted and confirmed, dams 5-7 were missed in aerial photo examination. Dam 5 was obscured by canopy cover; dam 6 was thought to be overhanging vegetation cover rather than a dam. Map Data © 2018 Google.


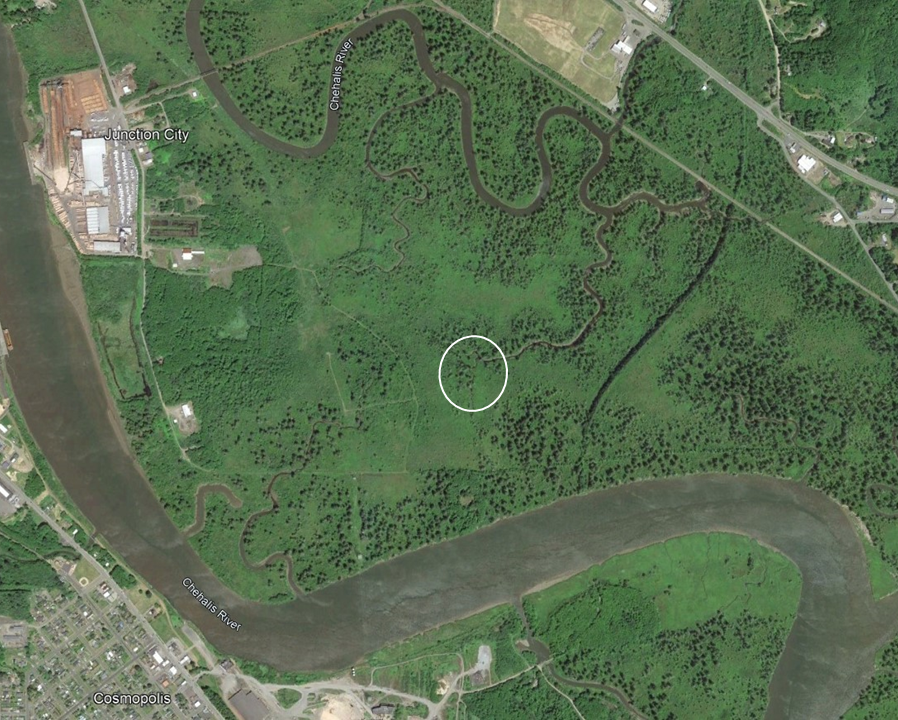


**Figure S21**. Elliott Slough vicinity in the Chehalis River tidal floodplain with area of almost certain beaver dam presence circled in white. The town of Cosmopolis, Washington is in the lower left corner. Map Data © 2017 Google.


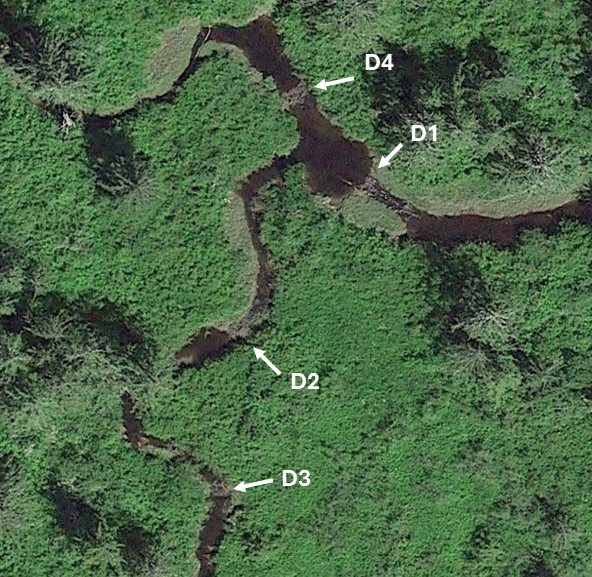


**Figure S22**. Elliott Slough detail with almost certain tidal beaver dams indicated. Signs of likely dams include visible dam (distinct from a fallen log), turbulent water (white specks) trailing from the dam, and a sudden widening of the channel above the dam. All three indicators are present for dams 1 & 2. Dam 3 shows a visible dam and widening of the channel. Dam 4 only shows a clearly visible and organized structure of small wood. These dams were not ground-truthed. Map Data © 2017 Google.


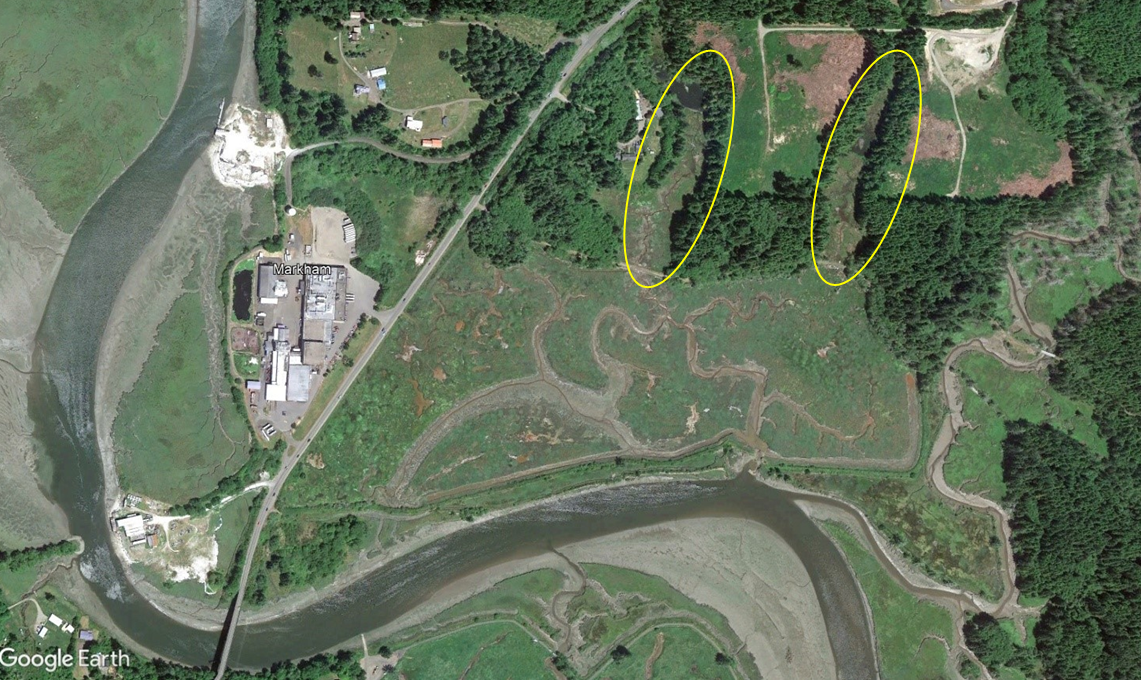


**Figure S23**. Johns River vicinity, with ground truth sites circled. Map Data © 2021 Google.
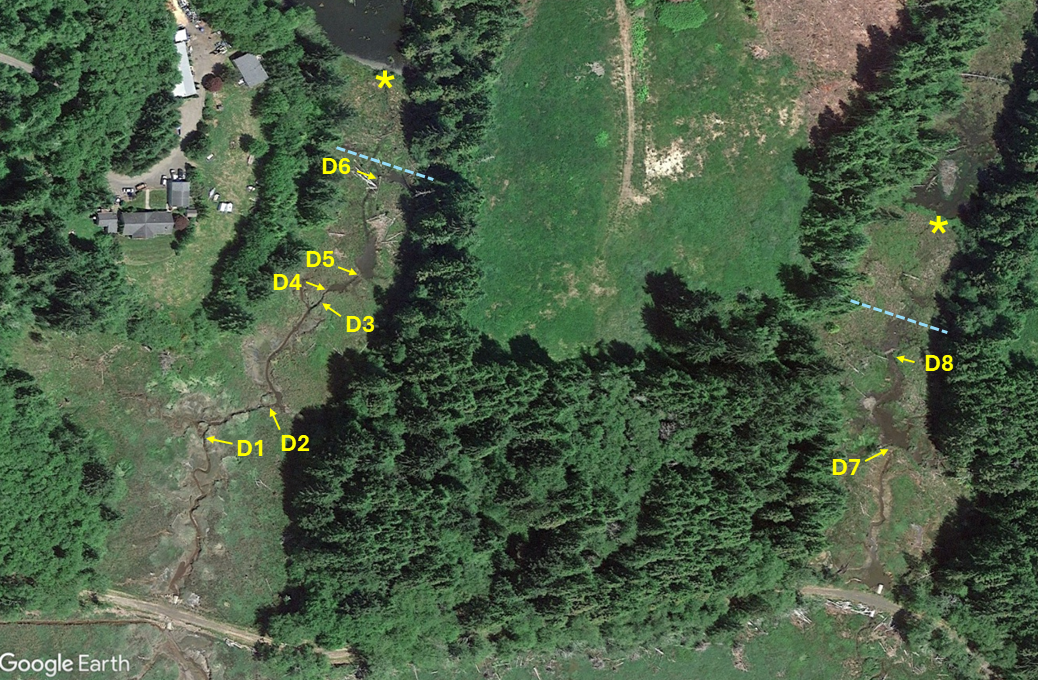


**Figure S24**. Ground-truthed Johns River sites. Dams 1-3 & 7 were missed in aerial photo scouting; other dams were correctly predicted. Blue dashed lines are estimated limits of high tides based on vegetation change. Tidal areas were dominated by *Carex lyngbyei* (sedge) with scattered *Argentina egedii* (Pacific silverweed); near the road were large patches of *Distichlis spicata* (saltgrass) with scattered *Triglochin maritima* (seaside arrowgrass) and *Grindelia integrifolia* (gumweed) indicating a gradient from mesohaline marsh near the road to oligohaline further away. Beaver are likely resident in ponds above the principal dams marked by an asterisk. Map Data © 2021 Google.


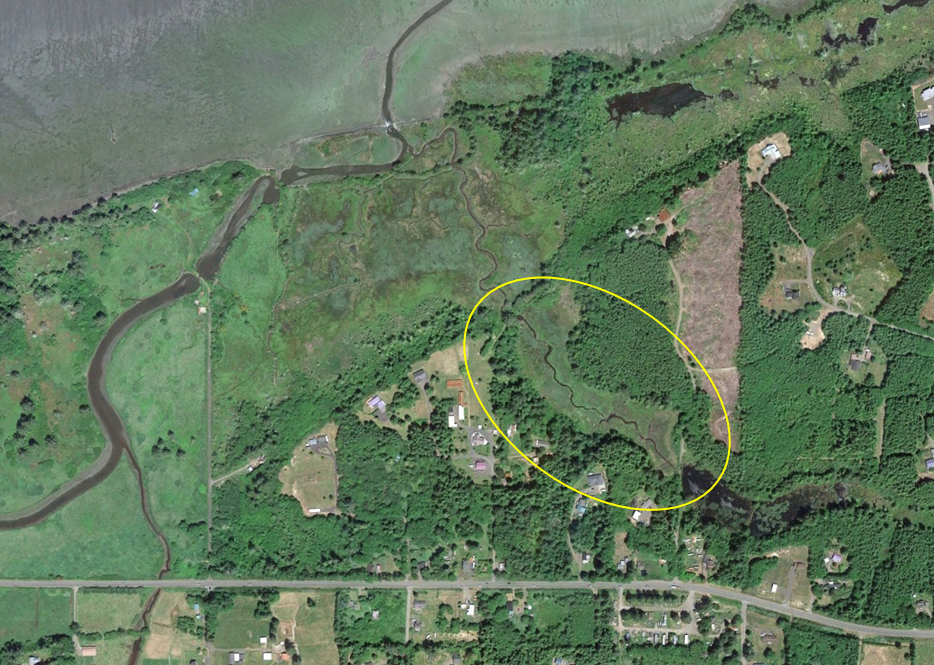


**Figure S25**. Vicinity of ground-truth site near Bottle Beach State Park, East Fork of Redman Slough. Map Data © 2021 Google.


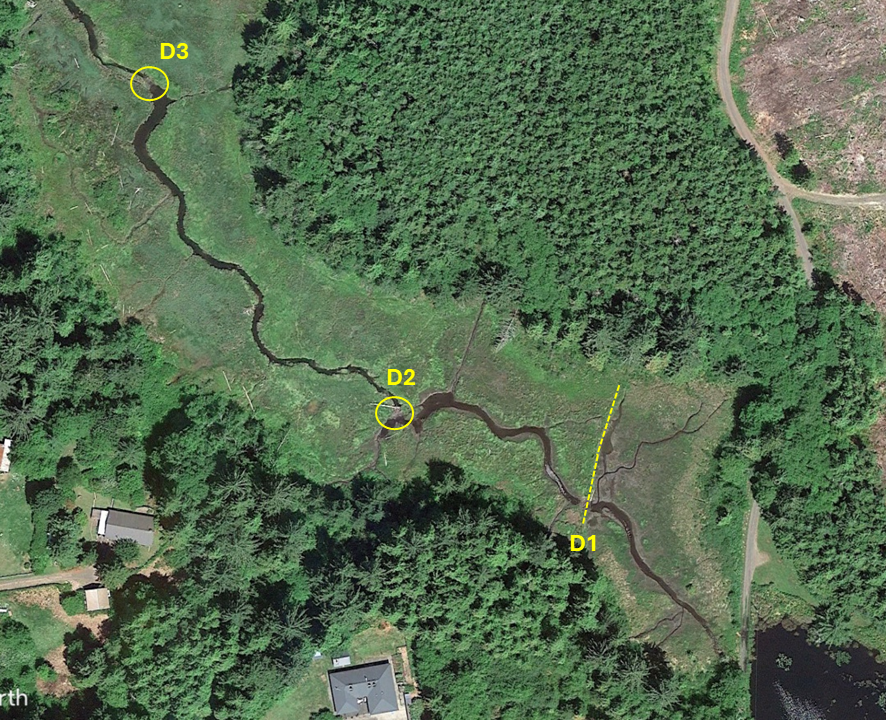


**Figure S26**. Detail of ground-truth of East Fork of Redman Slough. Dam 1 extended beyond the channel, well into the tidal marsh (dashed line). All dams were correctly predicted from prior aerial photo examination. Marsh vegetation was dominated by *Juncus balticus* (Baltic rush), *Agrostis stolonifera* (creeping bentgrass) and *Argentina egedii* (Pacific silverweed), which suggest high marsh elevations and low mesohaline to oligohaline conditions. The tidal pond in the low-right corner is formed by impoundment from an undersized road culvert. Map Data © 2021 Google.


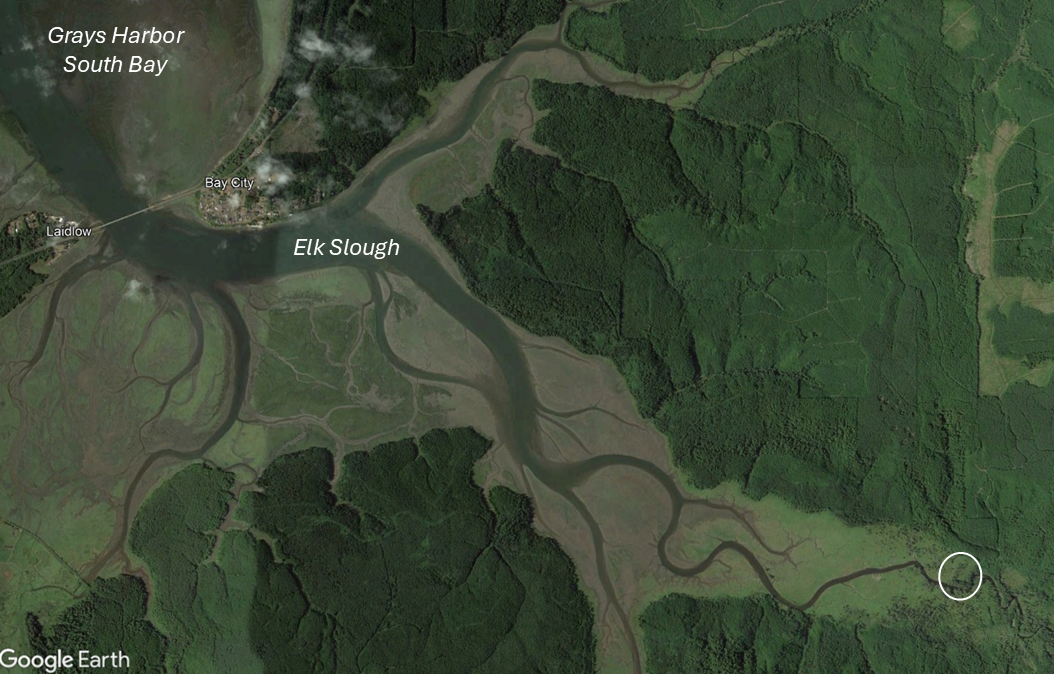


**Figure S27**. Elk Slough vicinity with area of almost certain beaver dams circled in white. Map Data © 2021 Google.


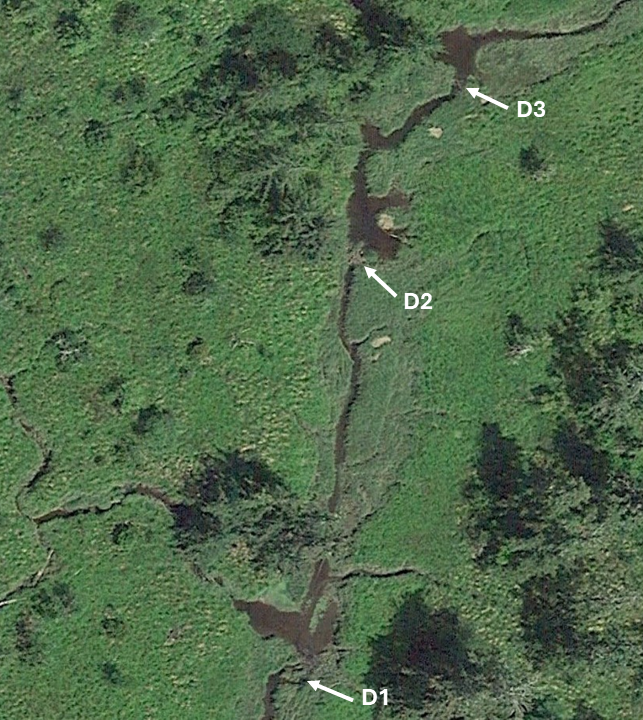


**Figure S28**. Elk Slough detail with almost certain tidal beaver dams indicated. Dam 1 is likely a composite of sticks packed with mud that has been colonized by herbaceous vegetation. Dams 2 and 3 appear to clearly have constituent sticks visible. All three dams show a sudden widening in the channel above the dam. These dams were not ground-truthed. Map Data © 2017 Google.


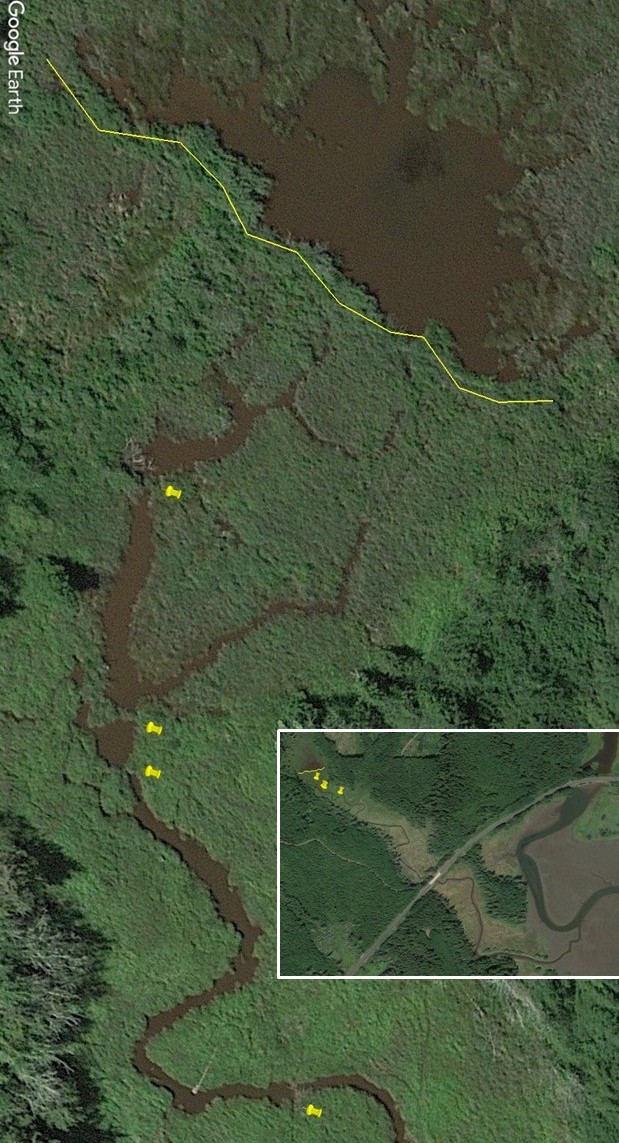


**Figure S29**. Norris Slough beaver dams (yellow pins) evident crossing the tidal channel; yellow line is probable location of a marsh surface dam (110 m) creating the upstream impoundment. This site was not ground-truthed. Inset shows vicinity, with Norris Slough draining to Willapa Bay at low tide. Map Data © 2022 Google.


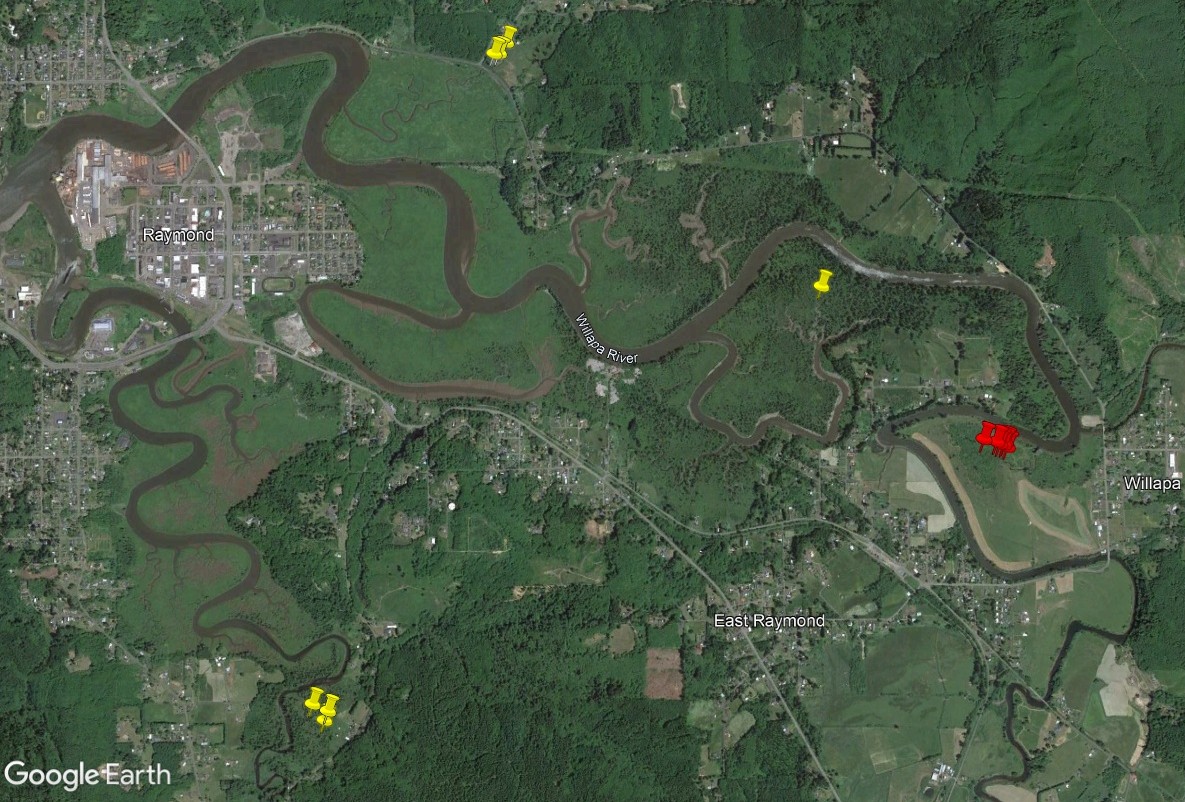


**Figure S30**. Vicinity map of beaver dams in the tidal reaches of the Willapa River. Red pins are dams confirmed by property owner. Yellow pins are not ground-truthed, but probable according to photo interpretation. Map Data © 2017 Google.


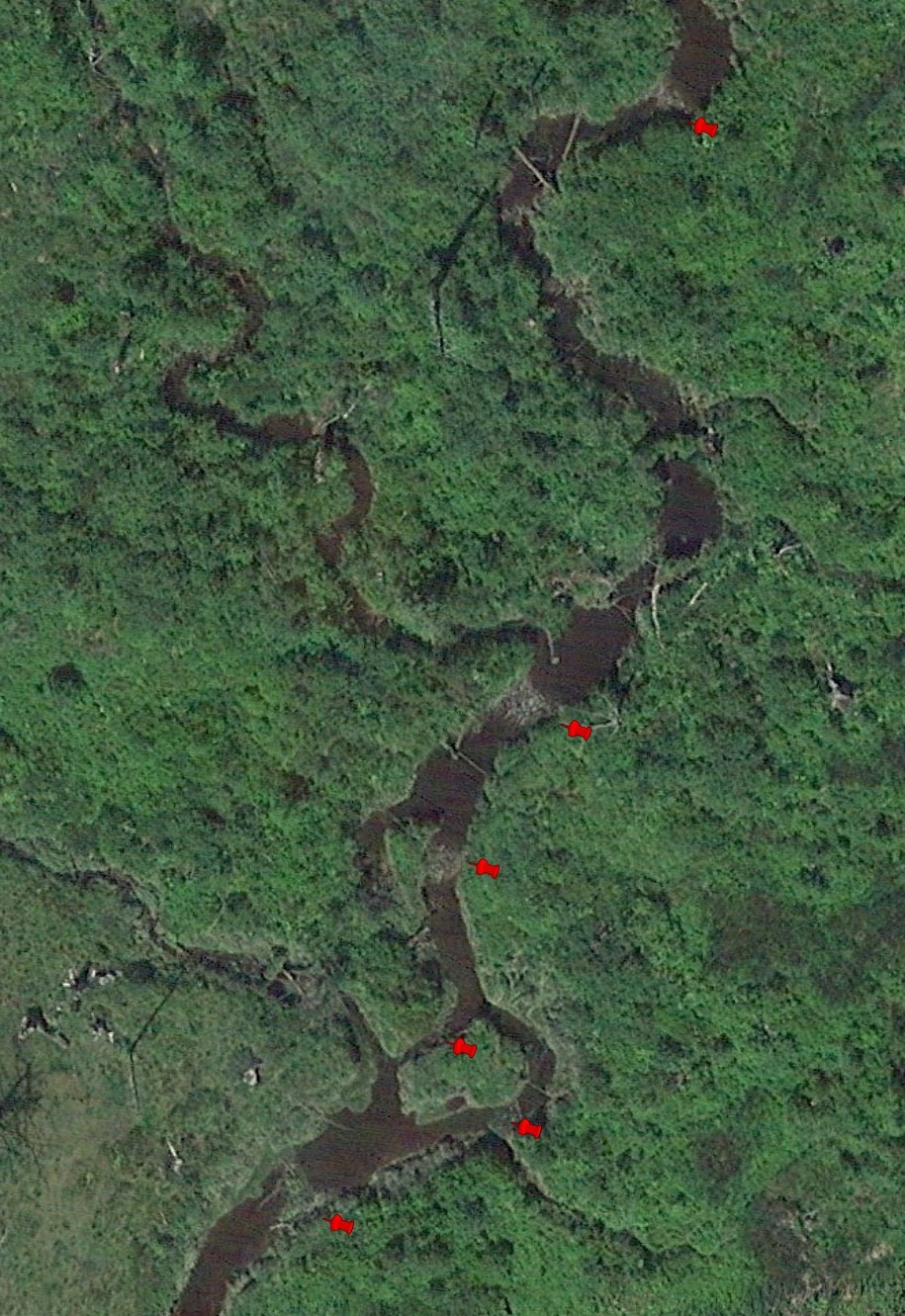


**Figure S31**. Detail of farmer confirmed dams. Property access was not granted due to concern about disturbing calving livestock, but farmer confirmed presence of beaver and flooding of parts of his adjacent property even at low tide. Map Data © 2017 Google.


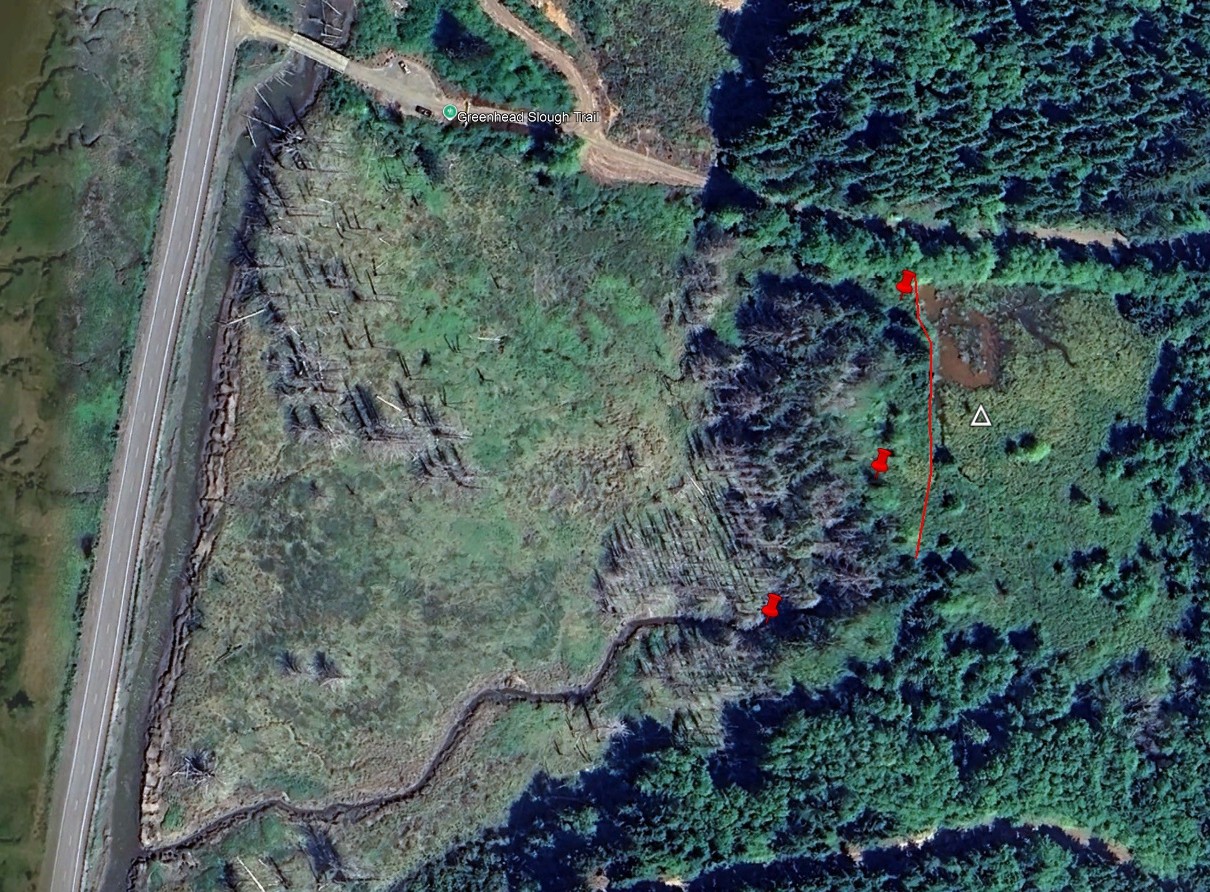


**Figure S32**. Ground-truthed beaver dams (red pins) and marsh surface dam (red line) and field-observed beaver lodge (white triangle) on a tributary to Greenhead Slough in southern Willapa Bay. The marsh surface dam was about 0.5 m high. Map Data © 2021 Google.


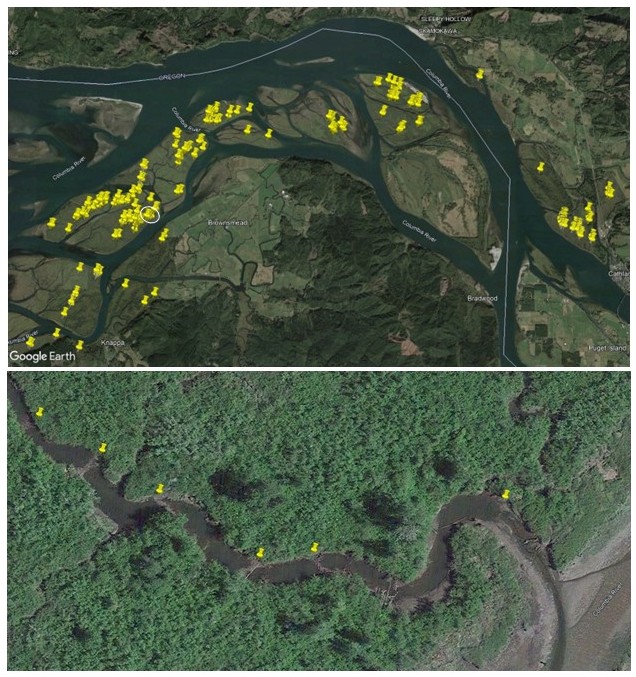


**Figure S33**. [Top] Tidal beaver dams (yellow pins) in the lower Columbia River estuary, just upstream of Astoria. All dams are inferred from photo-interpretation; no ground-truthing. Tidal range is approximately 3 m in this area. [Bottom] Detail showing clear indications of beaver dams spanning a tidal channel. Location coincides with white oval in the top frame aerial photograph. Note low-tide exposure of river margin in lower right corner of the photo. Map Data © 2017 Google.


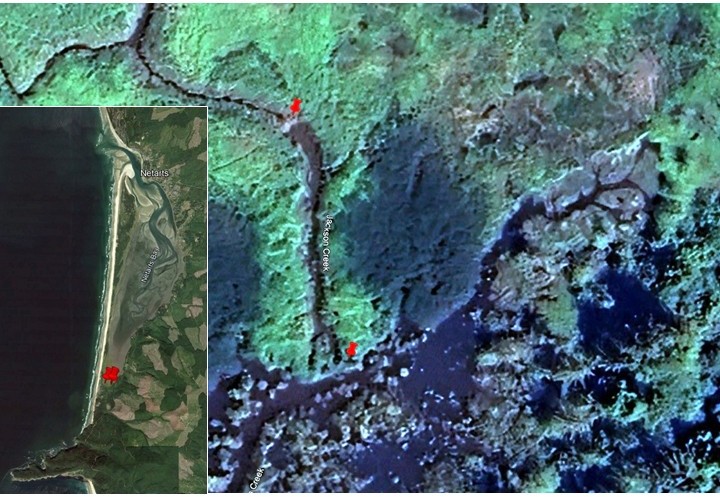


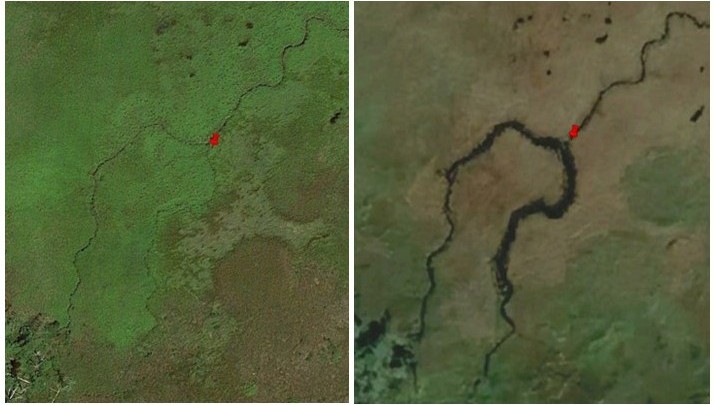


**Figure S34**. [Top-inset] Netarts Bay (Oregon) vicinity at low tide and tide flats exposed with ground-truthed beaver dams (red pins). [Top-right] Detail of two beaver dam locations; top dam had a head of about 0.75 m, bottom dam a head of about 1.5 m; extensive ponding below the bottom dam. Freshwater inflow from south via Jackson Creek. [Bottom] Comparison of tidal channel without (left: 2017) and with (right: 2020) a beaver dam in the Netarts Bay marsh. Map Data © 2017 and 2020 Google


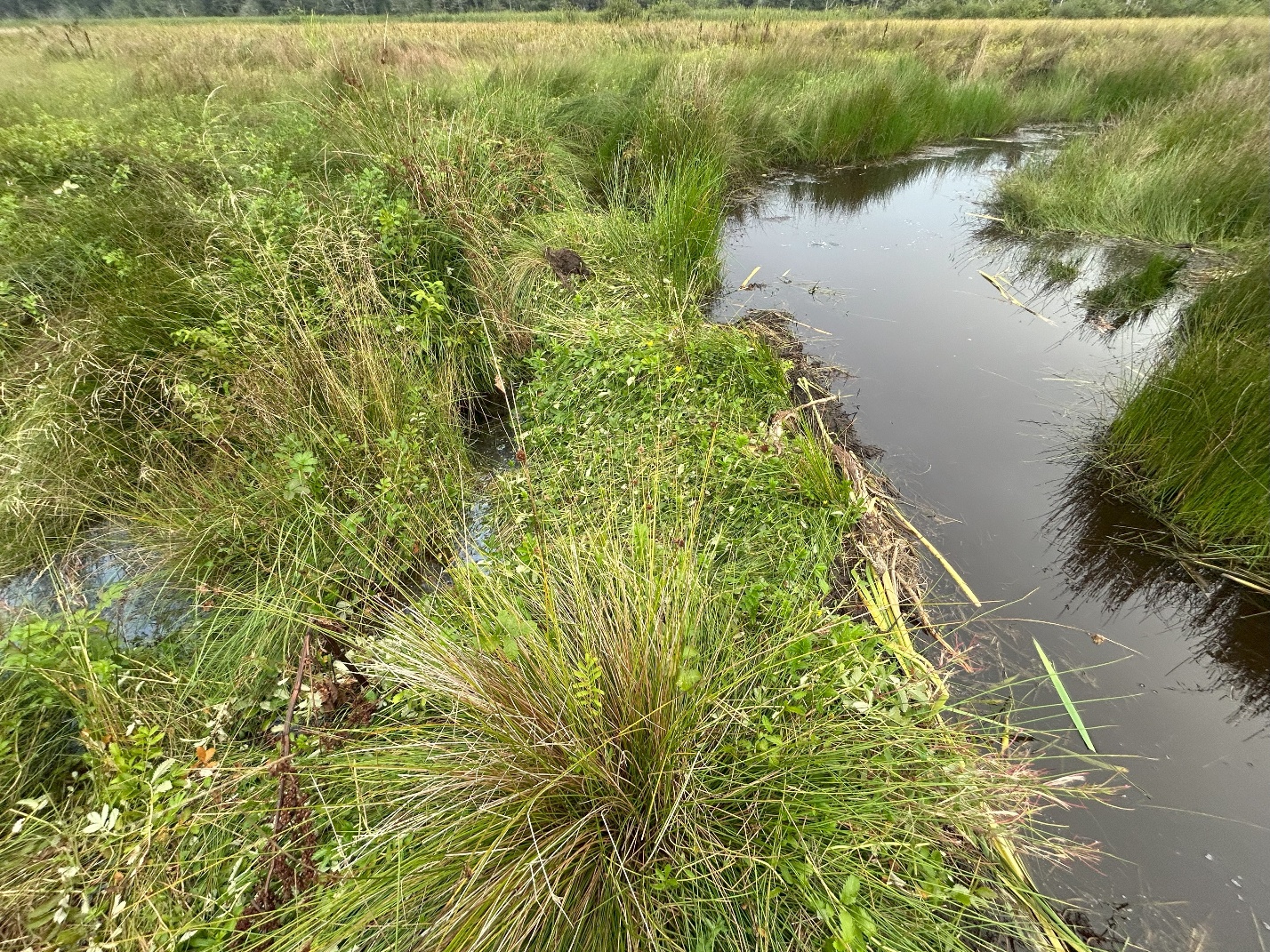


**Figure S35**. Beaver dam in Netarts Bay (45.367° N, 123.967° W). This is the only dam I have seen that appears to have little if any woody material; it seems to be composed entirely of herbaceous vegetation and mud. The dam is difficult to distinguish, but the pond is to the right, and a draining channel is to the left; dam head is about 1 m. Common vegetation in this area was spikerush (*Eleocharis palustris*), Pacific silverleaf (*Argentina egedii*), and tufted hairgrass (*Deschampsia caespitosa*). Sitka spruce swamp borders the tidal marsh at higher elevations. Photo by the author.


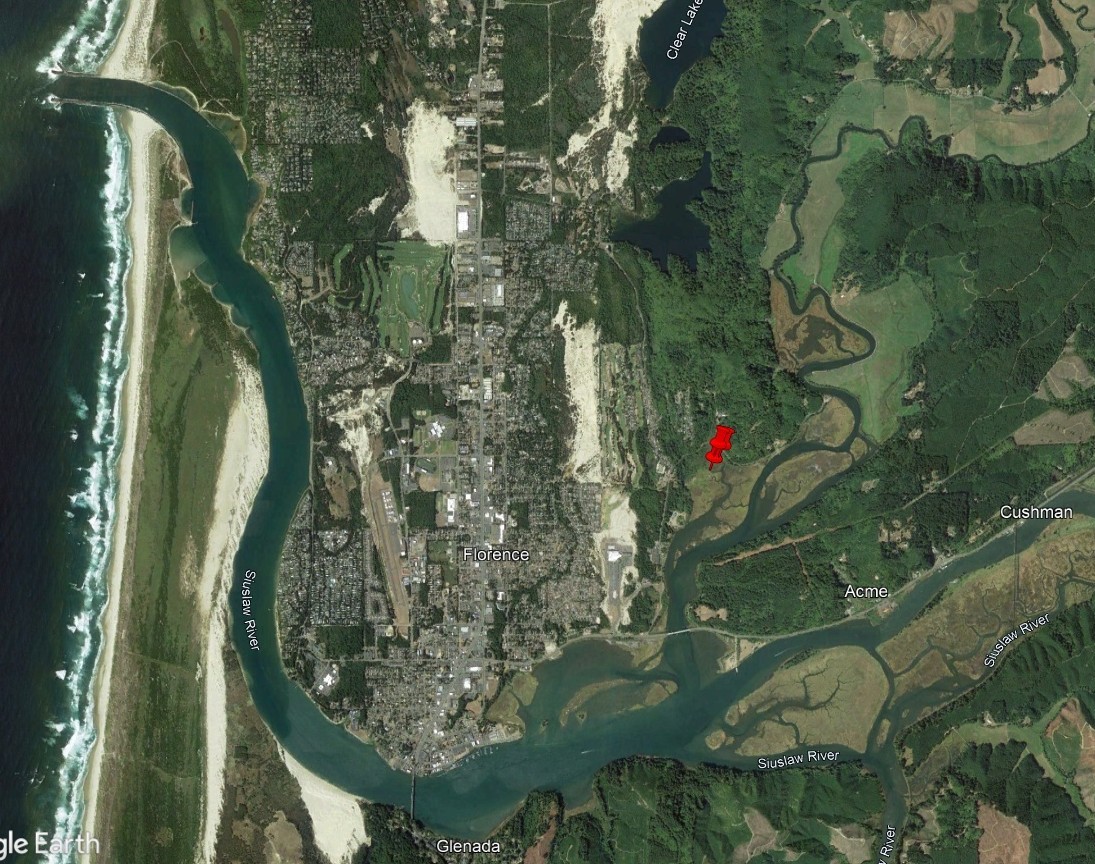


**Figure S36**. Vicinity map of ground-truthed beaver dams (red pins) in the North Fork Siuslaw River, near Florence, Oregon. Map Data © 2016 Google.


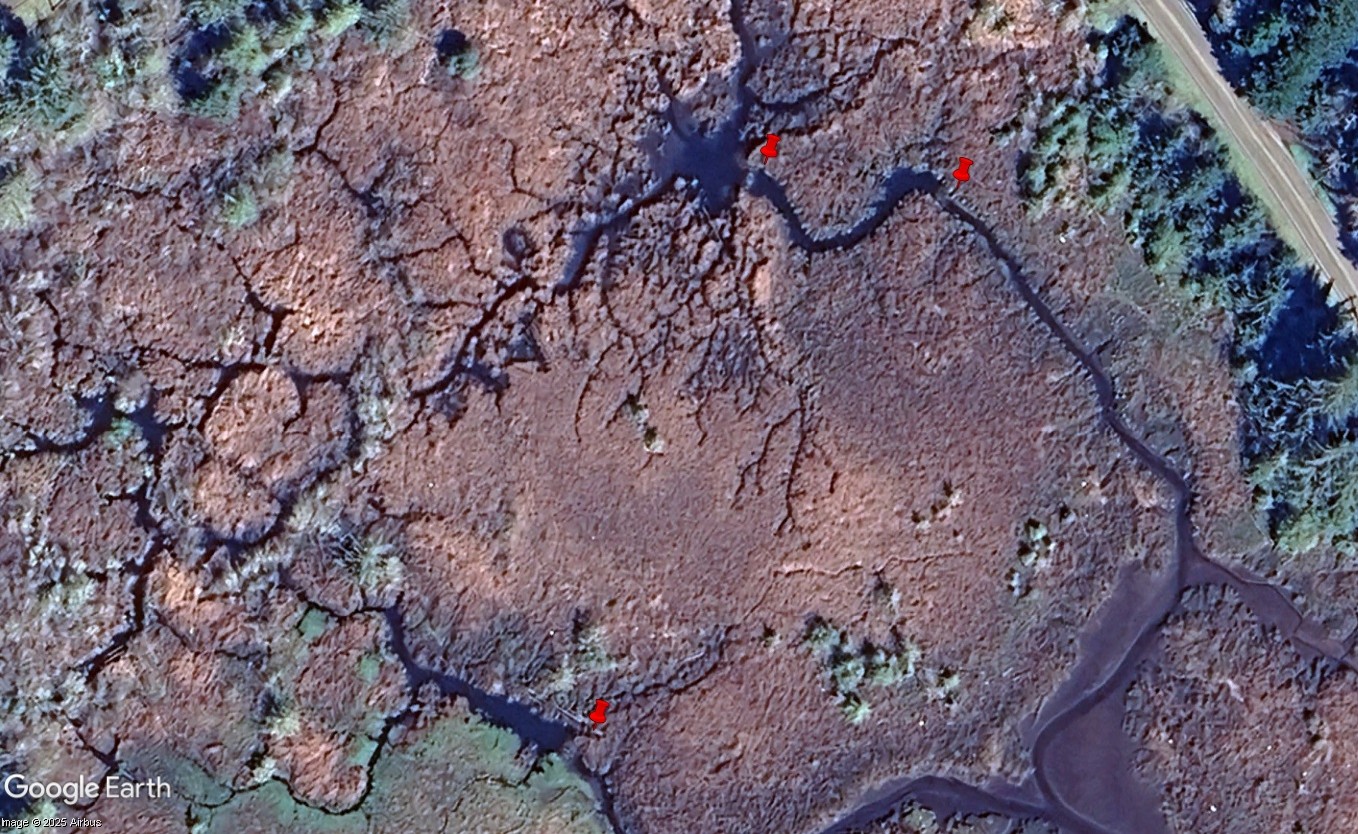


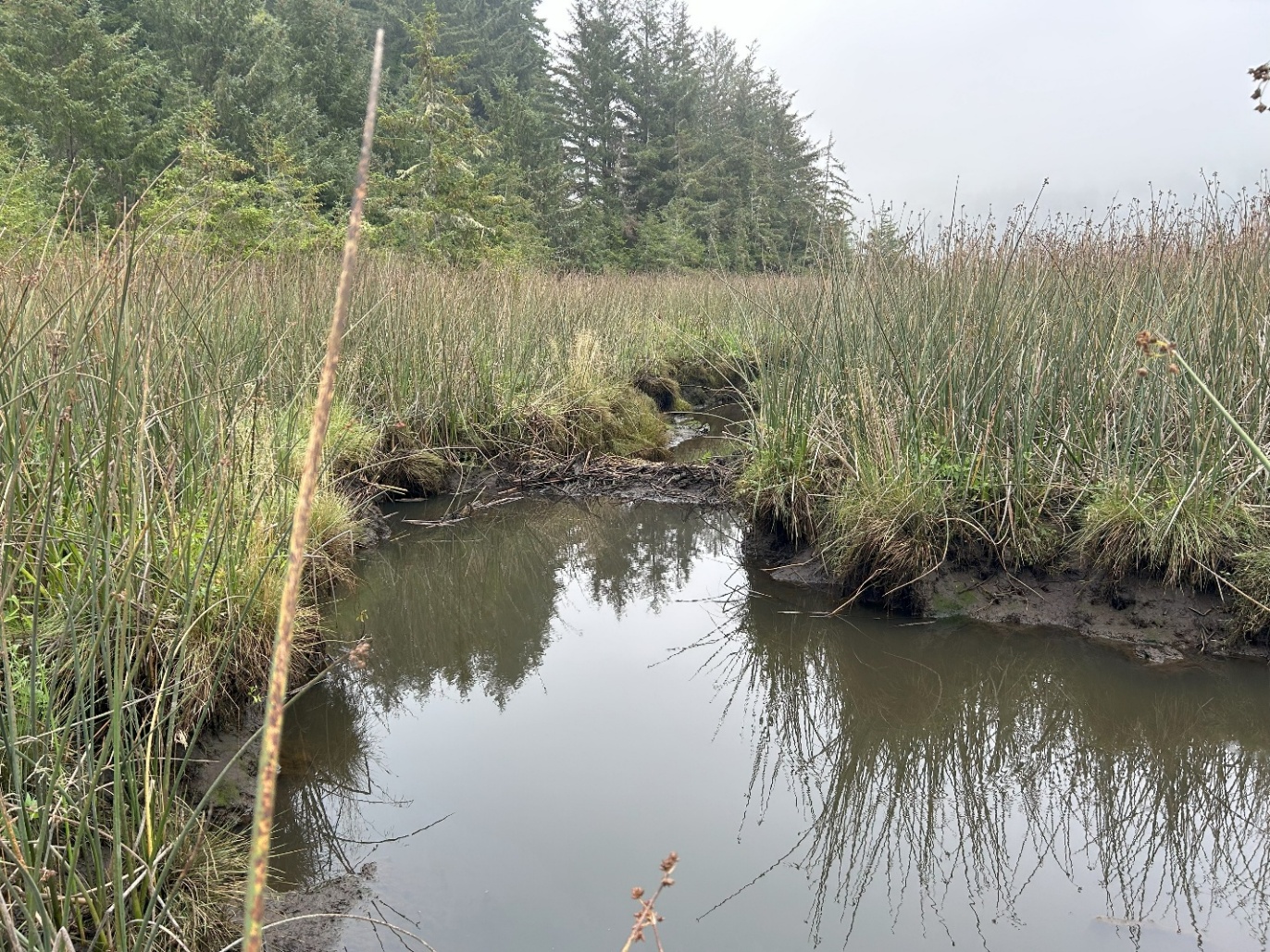


**Figure S37**. [Top] Ground-truthed dam locations (red pins) in North Fork Siuslaw River. Map Data: Google © 2025 Airbus. [Bottom] Beaver dam in the North Fork Siuslaw Estuary, Oregon (43.990° N, 122.077° W). Photo by the author. The dam head was 1 m; pond is in the foreground, draining channel in the background. The dominant vegetation is soft-stem bulrush (*Schoenoplectus tabernaemontani*). Higher high tides flood the marsh surface.


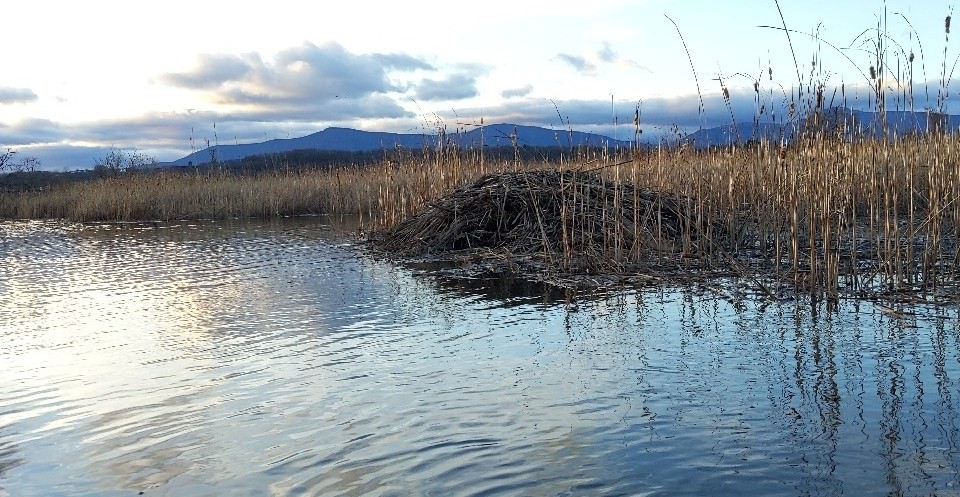

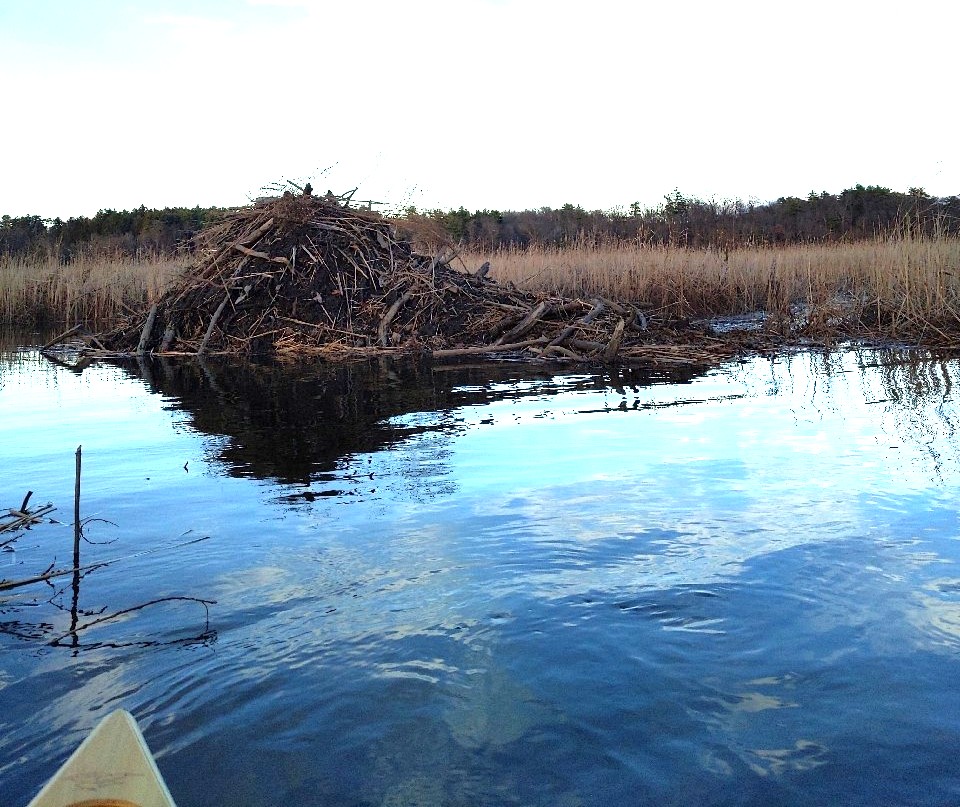


**Figure S38**. Two beaver lodges photographed by Jack Loud (Bard College student) at the Tivoli Bays in the Hudson River National Estuarine Research Reserve (New York) where there is a 1.7-m tidal range.
