# Supplementary material for: Beaver in tidal habitat: Examples from the Pacific Northwest
Source: PLoS One. 2026 Jul 8;21(7):e0349313. doi: 10.1371/journal.pone.0349313 (PMC13345279; doi:10.1371/journal.pone.0349313)
Supplement: Attachment — Submitted filename: Response to Reviewers.docx [file pone.0349313.s003.docx]

Please include the following items when submitting your revised manuscript:

- A letter that responds to each point raised by the academic editor and reviewer(s). You should upload this letter as a separate file labeled 'Response to Reviewers'.
- A marked-up copy of your manuscript that highlights changes made to the original version. You should upload this as a separate file labeled 'Revised Manuscript with Track Changes'.
- An unmarked version of your revised paper without tracked changes. You should upload this as a separate file labeled 'Manuscript'.

Journal requirements:

When submitting your revision, we need you to address these additional requirements.

1. Please ensure that your manuscript meets PLOS ONE's style requirements, including those for file naming. The PLOS ONE style templates can be found at

<https://journals.plos.org/plosone/s/file?id=wjVg/PLOSOne_formatting_sample_main_body.pdf> and

<https://journals.plos.org/plosone/s/file?id=ba62/PLOSOne_formatting_sample_title_authors_affiliations.pdf>

3. When completing the data availability statement of the submission form, you indicated that you will make your data available on acceptance. We strongly recommend all authors decide on a data sharing plan before acceptance, as the process can be lengthy and hold up publication timelines. Please note that, though access restrictions are acceptable now, your entire data will need to be made freely accessible if your manuscript is accepted for publication. This policy applies to all data except where public deposition would breach compliance with the protocol approved by your research ethics board. If you are unable to adhere to our open data policy, please kindly revise your statement to explain your reasoning and we will seek the editor's input on an exemption. Please be assured that, once you have provided your new statement, the assessment of your exemption will not hold up the peer review process.

4. We note that Figures 1, 2, 3, 5, 6, 7, 9, 10, 11,               S1, S10, S11, S12, S13, S14, S15, S16, S17, S18, S19, S20, S21, S22, S23, S24, S25, S26, S2, S28, S29, S30, S31, S32, S33, S34, S36, S37, and S39 in your submission contain [map/satellite] images which may be copyrighted. All PLOS content is published under the Creative Commons Attribution License (CC BY 4.0), which means that the manuscript, images, and Supporting Information files will be freely available online, and any third party is permitted to access, download, copy, distribute, and use these materials in any way, even commercially, with proper attribution. For these reasons, we cannot publish previously copyrighted maps or satellite images created using proprietary data, such as Google software (Google Maps, Street View, and Earth). For more information, see our copyright guidelines: <http://journals.plos.org/plosone/s/licenses-and-copyright>.

We require you to either (1) present written permission from the copyright holder to publish these figures specifically under the CC BY 4.0 license, or (2) remove the figures from your submission:

1. You may seek permission from the original copyright holder of Figures 1, 2, 3, 5, 6, 7, 9, 10, 11, S1, S10, S11, S12, S13, S14, S15, S16, S17, S18, S19, S20, S21, S22, S23, S24, S25, S26, S2, S28, S29, S30, S31, S32, S33, S34, S36, S37, and S39 to publish the content specifically under the CC BY 4.0 license.

We recommend that you contact the original copyright holder with the Content Permission Form (<http://journals.plos.org/plosone/s/file?id=7c09/content-permission-form.pdf>) and the following text:

“I request permission for the open-access journal PLOS ONE to publish XXX under the Creative Commons Attribution License (CCAL) CC BY 4.0 (<http://creativecommons.org/licenses/by/4.0/>). Please be aware that this license allows unrestricted use and distribution, even commercially, by third parties. Please reply and provide explicit written permission to publish XXX under a CC BY license and complete the attached form.”

Please upload the completed Content Permission Form or other proof of granted permissions as an "Other" file with your submission.

**Author’s Response:**

**I have consulted the copyright acknowledgement requirements for Google, ESRI, and Pictometry (Eagleview Inc.), the sources the aerial imagery in my figures. None of them require requests for permission and Google specifically says that they do not have time to respond to such requests. Instead, they provide clear guidance for when their imagery can be use and how to acknowledge their copyright. I have detailed my response regarding content permissions in an attached “Other” file for each source of imagery.**

In the figure caption of the copyrighted figure, please include the following text: “Reprinted from [ref] under a CC BY license, with permission from [name of publisher], original copyright [original copyright year].”

[Note: HTML markup is below. Please do not edit.]

Reviewers' comments:

Reviewer's Responses to Questions

**Comments to the Author**

1. Is the manuscript technically sound, and do the data support the conclusions?

The manuscript must describe a technically sound piece of scientific research with data that supports the conclusions. Experiments must have been conducted rigorously, with appropriate controls, replication, and sample sizes. The conclusions must be drawn appropriately based on the data presented.

Reviewer #1: Partly

Reviewer #2: Yes

Reviewer #3: Partly

2. Has the statistical analysis been performed appropriately and rigorously?

Reviewer #1: Yes

Reviewer #2: Yes

Reviewer #3: Yes

3. Have the authors made all data underlying the findings in their manuscript fully available?

The [PLOS Data policy](https://urldefense.proofpoint.com/v2/url?u=http-3A__track.editorialmanager.com_CL0_http-3A-252F-252Fwww.plosone.org-252Fstatic-252Fpolicies.action-2523sharing_1_010f019b96ef7641-2D09209c40-2Db351-2D4770-2Da68b-2Dd3dcdd12165c-2D000000_jtbeyprUoTpiwvUk31hPioh0Bv7kURZjCPN-5FjY8zn3Q-3D244&d=DwMFaQ&c=euGZstcaTDllvimEN8b7jXrwqOf-v5A_CdpgnVfiiMM&r=QlkINh0EQmIiueqOcPX6kWodPZEARlpGwF2jgcLvEMU&m=SYHhG6yASw0Cf_8N0-TBabmZp5KsySilpEevcKukDelmyp-U35MpkeetxQ_4c9_T&s=545gXt4AefrW4cwkII6mCOyzohxU8XnqVFcdbwqJkw8&e=) requires authors to make all data underlying the findings described in their manuscript fully available without restriction, with rare exception (please refer to the Data Availability Statement in the manuscript PDF file). The data should be provided as part of the manuscript or its supporting information, or deposited to a public repository. For example, in addition to summary statistics, the data points behind means, medians and variance measures should be available. If there are restrictions on publicly sharing data—e.g. participant privacy or use of data from a third party—those must be specified.

Reviewer #1: No

Reviewer #2: Yes

Reviewer #3: No

4. Is the manuscript presented in an intelligible fashion and written in standard English?

Reviewer #1: No

Reviewer #2: Yes

Reviewer #3: Yes

5. Review Comments to the Author

Please use the space provided to explain your answers to the questions above. You may also include additional comments for the author, including concerns about dual publication, research ethics, or publication ethics. (Please upload your review as an attachment if it exceeds 20,000 characters)

Reviewer #1:

This is a very interesting journal article where the author demonstrates significant knowledge about the system and includes an impressive amount of fieldwork, documenting beavers pushing into marine habitats, something that can significantly extend the knowledge and understanding of beavers. I want to congratulate the author on recognizing this pattern and aiming to publish this important contribution!
Unfortunately, in its current form, the manuscript **suffers from a lack of clear research questions, clarity in the result and method section and fails to highlight the potential implications of this paper (which are definitely there)**. I think with some work, especially on introduction, presentation of the results and discussion, these limitations can be addressed, and this paper can fulfil its full potential to an international readership.

**RESPONSE:** I have clarified to research objectives of the paper in the Introduction and I have added a Summary after the Discussion to clarify the “take-home” messages of the paper and highlight the implications of the paper.

Especially crucial is to rework the result section. Full of jargon and site names, I got lost a lot trying to understand where we are and what is being talked about. For a primarily freshwater ecologist (which most beaver people are), this was challenging, and marine terms and context need a much better introduction.

**RESPONSE:** I added a paragraph in the Introduction and in the Study Areas section of the Methods to provide a brief primer on tidal systems, including necessary jargon. Unfortunately, this jargon is essential vocabulary for estuarine ecology.

In my opinion, the author has to decide where this story should be published and in what format. If it is to be published in an internation journal with the aim to put the idea out that beavers also live in tidal habitats, then the manuscript needs to be trimmed and generalized for the internation reader. If the author would like to tell a more detailed story including discussing site specifics, then a local naturalist journal might be the better choice.

**Abstract**

Line 22 Please spell out or remove the abbreviation RTK

**RESPONSE:** I spelled out the abbreviation.

Line 24 Please mention density per km

**RESPONSE:** Changed as suggested.

**Introduction**
General comments
Nice introduction with very good sections. Some parts can be shortened/condensed (BDA’s, part on fish from Line 51). Given this has the ambition to be a naturalist paper informing the international readership about the extraordinary capabilities of beavers extending into tidal marches, **I would like some more description of the habitat and beaver biology compared to the more standard beaver freshwater stream habitat**. **Some of that information may be in the methods already, I would consider moving it**. This will help the reader understand why beaver colonize these habitats and ultimately also how that could be applied to other areas.

**RESPONSE:** I added a paragraph to the Introduction that summarizes the principal differences between fluvial and tidal systems that beaver, and beaver biologists, must confront. I think this will be useful for beaver biologists who are typically unfamiliar with tidal ecosystems.

Additionally, from the current introduction, the research questions are not so clear to me and that could be achieved with a better structuring of the introduction clearly highlighting knowledge gaps.

**RESPONSE:** The issue of greater clarity in the goals of the paper was also mentioned by reviewer #3. I have edited the Introduction to clarify the structure of the paper and the goals of each structural component. I have also edited the Discussion to circle back on the goals described in the Introduction. With regard to highlighting knowledge gaps, there is almost nothing known about tidal beaver—almost everything is a knowledge gap (which I point out in the new edits), but I hope I have made it clear that I am addressing two particular gaps and pointing out the need for greater attention to tidal beaver in general.

Finally, I miss an overview or at least the question of other places where beavers push into saltwater/marine habitats. Is this know from other places than the Pacific Northwest? I think some literature is available. I think also beavers in Argentina were known to colonize islands separated by saltwater and feed on marine food like algae. Check Anderson et al. 2008 Mammal review doi: 10.1111/j.1365-2907.2008.00136.x as a starter.

**RESPONSE:** Yes, this story is well known. Beaver can swim through salty seas to disperse to islands or along shorelines. This has also been observed in Puget Sound. But dispersal is temporary transit. Occupation of estuarine (brackish) habitat is long-term and a much different phenomenon.

Line 35 please specify where each beaver is native (broadly) and which beaver species is dealt with in this manuscript.

**RESPONSE:** Parenthetical notes were made for each species at this point in the text. Given that the fieldwork in this manuscript takes place in North America, it did not seem necessary to explicitly say that this paper focused solely on C. canadensis—also there are literature-based comparisons of lodges and dams in a couple of tables that include both species, so to say that this paper is exclusively about C. canadensis is not precisely accurate. Both species have a lot in common, which is why I included C. fiber in the literature-based comparisons.

**Line 60 I am not sure why suddenly the text is about BDA’s so much. I understand that there seems to be a management/knowledge gap, but I am not sure the specific examples (maybe limit to one) add much for the international reader. Maybe that is also something for the discussion?**

**RESPONSE:** I have moved this text to the Discussion section dealing with habitat restoration. I have also edited it slightly to be more concise.

Line 65 As mentioned in the comment below, I would expand a little bit the physical differences of tidal ecosystems to more linear stream ecosystems. Maybe also worth to expand more generally about differences in biology of the beaver (why is it in the tidal marshes, what is it building dams out of, what is it feeding on)

**RESPONSE:** Line 65 (and this whole paragraph in general) has been moved to the Discussion, following the recommendation of another reviewer. I have also added a paragraph to the Introduction that describes the physical differences between fluvial and tidal systems, including differences in channel network geometry. However, with regard to differences in the biology of beaver between the two systems, nothing is actually known about these differences—there is no literature on this subject. The purpose of this paper is to explore some of these questions of tidal beaver biology. Thus, rather than discussing this in the Introduction, I think it more appropriate to do so in the Discussion.

Line 70 From here on, I got confused. The first sentence is clear, but then I feel the last sentence might be more appropriate and I also do not understand why the small stream are inhospitable.

**RESPONSE:** I am glad to see that the reviewer liked both sentences. I think they bracket the paragraph well. However, I revised the last part of the last sentence to clarify why small streams are inhospitable (low inflow = high salinity).

Maybe a broader introduction into the dynamics of tidal habitats could help follow the reader here. The sentence from line 71 I would move to the end and start with something like “to do this, we choose the following study regions representing ….”

**RESPONSE:** I added this recommended detail to the second paragraph of the “Study areas” section of the methods, where I discuss tides and salinity.

**Methods**
Whatever you decide to do with the results, follow the same logic in the methods.
I think a simple map, or at least a better description where these sites are and how much they are separated would help the reader.

**RESPONSE:** Maps of this kind was presented in Figs 1-3 (Fig 1 was revised to include the local-scale map in response to another comment) and Fig 9 in the results section (the reviewer liked Fig 9 a lot). It is challenging to keep figures to a minimum and avoid redundancy.

I am not even sure the country has been mentioned.

**RESPONSE:** A parenthetical statement was added to address this in the 1^st^ line of the “Study Areas” section of the Methods.

I am generally confused by the many localities mentioned and specific histories that I am not sure are important for the paper’s questions.

**RESPONSE:** I have deleted mention of many of these localities in response to this reviewer’s comment and to reviewer #2’s recommendation that I be more “concise”.

Line 139 Spell out abbreviation for RTK

**RESPONSE:** Reviewer #3 made the same comment. Changed as suggested.

**Results**
The result need a lot of restructuring. Currently, this is not publishable, as it is hard to follow and often lost in details. I tried to suggest improvements, because I think the manuscript idea is great!

**RESPONSE:** In response to all three reviewers, I have edited the text to be more concise, remove unessential details (like the abundance of place names), and to focus on principal points.

I suggest 3-4 Figures, in total one per section.

**RESPONSE:** I have reduced the number of figures, but not to the extreme suggested by this reviewer.

Whitin the sections:
- Separate your results better. Answer the main questions.
- A lot of the site-specifics has to go to help the reader better follow. Consider to elaborate and describe site specifics in the appendix. The results need to be generalized (at least the text).

**RESPONSE:** In response to all three reviewers, I have edited the text to be more concise, and I have removed mention of place names as much as possible.

Maybe have two section types. First a section of the dams and transects (things you measured), then a section about naturalist history, eg how the dams are build (things you observed).

**RESPONSE:** I don’t think this distinction between measurement and observation is very clear or helpful. Measurements are a type of observation.

I think some of these Figure S1-S9 can go into a composite figure in the main manuscript to illustrate the habitat.

**RESPONSE:** I weighed this suggestion very carefully, because I love figures, but I also understand the need to be concise and focused. The Supporting Information document is a wonderful compromise although not every reader takes advantage of it. I don’t think even figures S1-9 do justice to the variety of tidal environments in which beaver dams can be found; all of the other figures in the Supplemental Information document are required for this. Thus, I have chosen to stay with the status quo rather than add another figure.

In turn, I would reduce the figures in the main manuscript (or do a composite figure) for Figures 1-6.

**RESPONSE:** I removed Figure 6 to follow this recommendation. Figure 6 did not add any significant information that was not already found in Figure 5, and similar figures are found in the Supporting Information.

Line 160 I do not understand how this 2000 m were selected, at random, is this everything or accessibility? This should be mentioned in the methods.

**RESPONSE:** I added text to the methods in the “RTK-GPS field surveys” section that clarified tidal channel selection.

Again I am a bit at loss which name refers to which area and a map and a clear structure/separation in methods results per area could definitely help. Also for the other areas, data about the size belongs to methods in my opinion.

**RESPONSE:** I have added another map frame to Fig. 1 that shows a larger scale view of the estuary, with the location of each study site clearly noted.

Line 162 to line 170 I would just mention in the results that due to challenging field conditions only half of the terrain could be sampled.

**RESPONSE:** These edits were made as recommended.

The speculation on line 170 belongs into the discussion, same with line 174 and line 179.

**RESPONSE:** It’s not actually speculation; it’s a calculation and a result, so I think it is reasonable to keep where it is.

Line 213 I am sorry, but I cannot follow this sentence. What is lodges per colony? Not even “colony” has been introduced as a term. While this might be clear for beaver people, it could create confusion for other readers. Also what does it has to do with Table 2?

**RESPONSE:** I agree that “beaver family” is a less confusing term than “colony”. I assume that beaver biologists use the less anthropomorphic term to sound more clinical, nevertheless that is the term of art in this field. When I first encountered the term “beaver colony” I quickly intuited that it was a formal synonym for the more casual “beaver family”. For anyone who is truly confused by the terminology, Google is a good resource. Otherwise, I think my statement that beaver colonies can occupy more than one lodge is completely straightforward.

Line 220 I suggest to move this table to supplementary materials. I like that you back up your claims with data, but would generally advice to reduce numbers of figures and tables in the main story to focus on the most important contributions.

**RESPONSE:** Reviewer #3 actually liked this table (and my use of tables generally) and thought the comparison in dam frequency between tidal and fluvial systems was noteworthy. Thus, I am keeping the table where it is. Instead, I will try to be more concise (as recommended by the other reviewers) in the text to better focus the story.

Line 223 First it was not clear to me that now lodges are reported. Maybe here (and in other places) separate sub-headings could help to guide the reader?

**RESPONSE:** Up to this point in the Results, I have been writing about “dams and lodges”. This and the next short paragraph are the only ones that focus exclusively on lodges, so I do not think these two paragraphs warrant distinction as a separate section. They are a reasonable extension of the preceding paragraphs.

Line 235 It is not entirely clear to me what a blind or a distributary channel is, this has not been introduced. Best place probably in the methods. Same with movement routes, this not be properly defined. What movement is it? Water, beaver? What is a route count?

**RESPONSE:** A parenthetical phrase “(branches)” was added after the first mention of the word, “distributary” in the “Study areas” section of the Methods. “Blind-end tidal channels” are mentioned in the “Study areas” section and a parenthetical phrase notes that the term will henceforth be shortened to “tidal channel”. I removed all mention of “blind” in the subsequent text. At the first mention of movement routes, I added the word “beaver” to modify the word “movement” to clarify that it is beaver that are moving.

Line 246 Maybe it helps to add to larger sampling region (here Snohomish) so the reader keep some simpler overview of the sites.

**RESPONSE:** I have added an overview map of the Snohomish sites in Fig. 1 and list the Snohomish sites in Table 1, so this should make the location of Quilceda clear enough. Also, the other two reviewers did not mention this. Finally, the reviewer did not appear to have a decisive opinion about this because they said, “Maybe”.

Line 255 What is the mean higher high water? Keep in mind, beaver people are often not very knowledgeable in marine jargon, you need to explain these things much better. Probably I miss some terms as well in this review, check again that you explained all terms very well. At this point I think a glossary could be helpful!

**RESPONSE:** I added text to define MHHW in the “Study areas” section of the Methods.

Additionally: What is navd88? Is it needed in the figure legend?

**RESPONSE:** I added an explanatory sentence in the Methods section dealing with RTK-GPS surveying.

Also clearly label which graph belongs to which line on the figure
Apply these changes to all figures, of course.

**RESPONSE:** I don’t think this is necessary because [1] one can intuit that the top graph is for the top channel trace in the aerial photographs, [2] one can confirm that intuition by comparing the location of beaver lodges in the graphs vs. the aerial photos, and [3] it’s not that critical if the reader somehow confuses one channel for another—these are illustrative and similar examples. Doing as the reviewer suggests would require adding unnecessary and cumbersome text to the legends.

Line 269 Why is there two red dots in the figure shown when it mentions: Other channel trace profile points are not shown to avoid graphic clutter

**RESPONSE:** This figure was removed in response to an earlier comment, so the issue is now moot.

Line 280, from “probably”: This is discussion up until the “provides an example” Also again from 297. I currently do not understand what the added value of the extensive description of this (seemingly) separate case study is. What does the reader learn that is important for the main story? Maybe some of this natural history and curiosity (which has its value!) can go in the description of the Fig S9?

**RESPONSE:** I have restructured and edited this paragraph to clarify that the reason I included this example of beaver colonization of senescing distributaries is that it clearly shows that the point of beaver dams in tidal channels includes allowing beaver to swim around at low tide, not just flooding the entrances of beaver lodges, i.e., low-tide swimming is not just an ancillary benefit, it is a principal purpose. I moved the discussion-like text to later in the paragraph, but had to retain it to put the current presence of beaver dams in context. I did remove discussion-like text from the end of the paragraph.

Line 327 I would separate the table and pit the literature results for freshwater into the appendix, and summarize them in text. Why has N different numbers, is this the sample size per “stream”?

**RESPONSE:** I did as the reviewer recommended. To be consistent I also moved Table 2, which similarly summarized literature results for freshwater dam and lodge density, to the Supplementary Information document. I renumbered all of the subsequent tables appropriately and did likewise for the literature citations in the text and tables.

Section starting from Line 340: Always use p < 0.001 if p-value is very small

**RESPONSE:** I have previously been advised to specify exact p-values because today’s computer software allow this specificity. Previous convention was to write “p << 0.001” if p-values were very small, but this is because p-value thresholds had to be looked up in statistical tables where they were typically limited to p = 0.10, 0.05, 0.01, 0.005, and 0.001.

Line 358 This could be the best figure of your manuscript, but currently I do not understand what is going on. Why is Snohomisch different to NF blind (what is NF) and NF distrib? What are the different regression lines for and why do the y differ from the ones in the text? I would suggest: Simplify the figure, show one regression per figure (overall). Show site-specific regressions in the appendix and condition specific regression only on the main text if they are significant.

**RESPONSE:** Reviewer 3 also commented on this figure, but his comment suggested the addition of two new graphs, which I followed. Reviewer 1 has elsewhere noted that the manuscript has a lot of figures and encouraged moving some of them to the Supplementary Materials sections or making compound figures, yet here they suggest the reverse—taking a compound figure and breaking it up into several independent ones. I have chosen to try to economize as much as possible on the number of figures, while still providing adequate information. Thus, I have kept the compound figure, while adding two graphs to it in response to reviewer 3.

Line 370 I find it hard to understand to follow this error matrix. Can you add a sentence that helps how to read it? For example: “reading example” In the North Fork area we predicted 121 dams, whereas we observed 69…” I would also move this table to the appendix and add an “overall” category

**RESPONSE:** Reviewer #3 also commented similarly. I have followed the recommendations of #3 which I think efficiently address the concerns of both reviewers.

Line 387 That is partly not results, but discussion

**RESPONSE:** Because the reviewer admits that it is “partly not results”, thus also partly results, I think it is reasonable to keep this in the results section. My further rationale is that this paragraph explains the lessons learned from the error analysis and these lessons were then applied (as mentioned in the next paragraph) to more confidently identify beaver dams in coastal wetlands throughout the region. Thus, this paragraph serves as an explanatory bridge to the next.

Line 393: I would suggest t move that into the appendix

**RESPONSE:** I think it is important to have one Figure in the main text that shows what beaver dams look like in an aerial photograph. Not everyone takes the extra effort to look at the Supplementary Materials and under some circumstances the SM may not be easily accessible.

Line 399 That is partly methods. I think you do not mention this part in the methods at all?

**RESPONSE:** This line refers to all of the previous results of the “Region-scale aerial photo surveys” section presented up to this point, which are methods only in that they are lessons learned from these results that are then applied to further, larger-scale regional photo evaluation. I respectfully disagree with the reviewer and note that neither of the other two reviewers took exception with this point.

Line 422 That is a very cool result and overview! **RESPONSE:** Thank you for the positive feedback!

Line 425 That is discussion from here

**RESPONSE:** I disagree, though I imagine that the phrase “from the perspective of beaver occupancy” might convey the impression that this is my interpretation. I have deleted this phrase and added the modifier “geomorphological” to “categories”. It is quite factual to say that large river systems are a distinct geomorphological category from small coast streams. The rest of the paragraph is thus an observational result.

Line 442 Another candidate for the supplementary materials

**RESPONSE:** I believe it is important to keep this figure in the main text because these types of beaver dams were so exceptional and appear to be particular to certain types of coastal wetlands, as described in the text.

Line 458 That is the sort of detail that can be probably safely removed from the manuscript without loosing too much knowledge. Same with the paragraph 460 onwards (maybe one sentence in the discussion)

**RESPONSE:** I deleted line 458, as recommended, but I disagree with the recommendation for the paragraph. First, this is a result, and second, it is important to note why I focus more on dams in photo interpretation and less on lodges.

Discussion
In general, the discussion is too long, often too far away from what the actual research topic was (a lot of speculation and open questions not backed up by the data) and lacking integration into other research. A lot of interesting points are there, but buried in the text. **Again I would emphasize not to use site names (only exceptionally), but instead generalize**. If people are interested in specific sites, they can still contact the author, but for most international readers the local site names do not matter.

**RESPONSE:** I have gone through the manuscript and reduced the use of site names where possible. However, I think elimination of sites names entirely is a mistake. Site distinctions are important because there are geological, hydrological, ecological, and sometimes historical differences between sites, which cause variation in whatever phenomenon is being observed. This is normal in ecology, and often interesting. Also, there are some readers who definitely like site names and find them important (I am one, and not just for my own papers), so the reviewer’s taste is not necessarily universally shared. Those who are not interested in the site names can gloss over them, as the reviewer appears to be implicitly doing, while those who find the distinctions interesting will appreciate the site names.

Line 493 Interesting, can you elaborate a bit more what you mean with freshwater habitat?

**RESPONSE:** I added two sentence phrases contrasting large estuarine systems “with high freshwater inflows and large coastal freshwater plumes” and small coastal systems “with small freshwater inflows” that I hope clarify the contrast for those unfamiliar with coastal wetlands.

Line 526 Lot’s of individual site names again in this paragraph, maybe it is possible to reduce these? Here you are trying to generalize, not to discuss individual cases. Maybe saying “some sites have high molbility, some low” is therefore sufficient.

**RESPONSE:** I have gone through the manuscript and reduced the use of site names where possible.

Line 535 I would avoid questions and frame it as research gaps: “It is unclear why”
Line 573 Again, I would avoid questions and frame it as research gaps: “It is unclear why”

**RESPONSE:** I respectfully disagree with the reviewer (and no other reviewers made this comment). I think this is a matter of style and it is related to the debate over whether to use the active (direct, first person) or passive (indirect, third person) voice in scientific writing. The argument for the active voice is that it is a more natural and more efficient way of writing, and it doesn’t pretend unrealistic standards of perfect, abstract objectivity. Science is done by imperfect humans. Likewise, posing a question directly, rather than posing it in the passive voice, is a more natural and efficient way of writing (it eliminate the first three words of the suggested text immediately) and it is a clear expression of human curiosity and thought processes, fundamental to science.

Line 598 For me that whole paragraph is speculation and has nothing to do with the presented data (no sound measurments done, not introduced in the introduction). It is interesting, but for me needs another study. Maybe keep 1-2 sentences.

**RESPONSE:** Reviewer #2 also suggested deleting this section. Since two reviewers had the same opinion, I followed their recommendation.

Line 625 For me, also these parts are discussed to much being to far from the research topic. If I am not mistake the increased fish densities were mentioned earlier already, so maybe there 1-2 points can be taken up

**RESPONSE:** From my perspective as a tribal biologist working in the Pacific Northwest, where recovery of threatened salmon species is a regional obsession, the implications of recognizing the possible importance of beaver biology to estuarine ecosystems and to the threatened salmon species that rear in them is very important. Beaver, of course, do not live in abstract isolation from the world. They are ecosystem engineers, likely even in tidal marshes, and they exist in relationship to humans as we do with them, so remarking on the likely significance of beaver to tidal marsh restoration in the Pacific Northwest, and likely elsewhere in the world, seems wholly appropriate. The work presented here clearly has implications for habitat restoration.

However, to address the concern of the reviewer that the Discussion is unwieldy, I have edited this section of the Discussion (as I have for other sections, for similar reasons) to reduce the word count by 49 and lines by 4, despite adding text to clarify why consideration of beaver in tidal marsh restoration is important.

**Reviewer #2**:

This is a well written piece and very interesting topic, though potentially to a limited audience, it still supports further knowledge expansion regarding a highly popular species of conservation and active restoration interest. I suggest minor revisions to improve readability.

Personally, suggest any rewriting of any first-person statements eg lines 53-55

**RESPONSE:** I am sensitive to this issue, because I am an older scientist (63) who was taught to avoid the active voice so as to avoid the suggestion of subjectivity in science. Most scientific journals now prefer, if not require, writing in the active voice, except possibly for the “Methods” section. For example, see the Oxford University Press’s comment on active vs. passive voice: <https://blog.oup.com/2018/01/first-person-pronouns-passive-voice-scientific-writing/#:~:text=The%20journal%20Nature%20is%20explicit%2C%20insisting%20that,found%E2%80%9D%20does%20make%20writing%20easier%2C%20even%20automatic>.

There are several arguments for this change: more efficient communication is prominent, but also acknowledgement that science is done by humans and has an element of subjectivity for better or worse. I think the for better is often underappreciated. I am still getting used to using the active voice (a part of me still feels a little uncomfortably narcissistic when I use it), so I used the first person only four times in the text: once in the Introduction, twice in the Results, and once in the Discussion. Incidentally, I did not use the first person in lines 53-55.

Any further exploration of Eurasian beavers in tidal zones, we certainly witness this but agree little documentation

**RESPONSE:** I am not sure what this comment means. Is the reviewer asking me to comment on European beaver in tidal zones? I am not personally familiar with European beaver, nor have I seen literature on tidal beaver in Europe (except for a couple of papers dealing with heavy metal contamination of beaver in a micro-tidal portion of the Rhine Delta, where tides were only a few cm, and where pollution was the focus rather than beaver ecology).

Line 57 reintroducing rather than introducing for correct conservation biology term - surely beavers here historically

**RESPONSE:** Changed, as suggested.

Detailed methodology and a range of variables discussed though I think I'm missing more on the wider beaver population density and history on when how why beavers have entered this system, I guess we presume it is population density pressure pushing beavers to live in theoretically more challenging and 'sub optimal' areas - are you seeing this as a result of this - therefore would releases into such areas (as briefly proposed) without other habitat infilling just result in released animals moving inland to 'easier' habitat and therefore release site fidelity would be challenging for conservation projects and a nice link for BDA investigations as pointed out - perhaps expand this point? Personally, I'm interested in how well are the beavers doing in these areas - any insight into body conditions, breeding stats etc?

**RESPONSE:** Answering “…why beavers have entered this system…” (and the other interesting questions posed by the reviewer) is a very large-scale question that is clearly beyond the scope of this paper. However, it is gratifying that I have provoked such questions in the reader. That was the intent.

It is interesting that people assume that tidal systems are “sub-optimal” compared to “traditional” fluvial systems. I am doubtful that this is the case; I suspect this attitude reflects our unfamiliarity with beaver in tidal systems, because tidal marshes have been so severely impacted historically (along with beaver). But, I agree that measurements of body conditions, breeding statistics, etc., would be helpful to confirm or dispel preconceptions. That is obviously beyond the scope of this paper, whose aim is to point out that tidal beaver are common (more so than even experts have previously realized) and that more attention should be paid to them—that there are many research questions to pursue here.

I have added three sentences to the end of the second paragraph in the Discussion (now moved so that it is the third paragraph) stating my opinion about the sufficiency of tidal beaver habitat and that data on tidal beaver body condition and breeding statistics are required to settle the point.

I deleted the 4^th^ paragraph for brevity as it was an indulgence.

Good use of tables to summarise results as some of the text can be a bit dense to read and take in - I just wonder is there any scope to put any more of the local scale field survey results into a table - just a question - like habitat/ forage type access?

**RESPONSE:** I have reviewed and edited the text to try to make it less dense. I do not believe there is any more opportunity to add tables. In fact, other reviewers wanted me to reduce the number of figures and tables.

Suggest there is repetition, e.g., use of aerial photos in the regional scale section - could be more concise here along with discussion lines 545-569 - please consider condensing

**RESPONSE:** The only image that I can imagine removing to be more concise without losing important information, would be the Potter Slough image in Fig. 11. I did not ground truth that particular site, so it is a logical choice for removal and doesn’t add much to the other two sites that I did ground truth. Fig. 9 also has three photos, but I think it is important to show the diversity of photo signatures that indicate the presence of beaver dams. Thus, Fig. 11 was edited to remove Potter Slough, and copyright was acknowledged according to Google’s instructions (see “Other” file). As suggested by the reviewer, to be more concise I removed a net total of 242 words from the text of the “regional scale section”, a 26% reduction; I also removed a net total of 56 words of text from lines 545-569, a 16% reduction.

Discussion - sound of water - I would consider this inclusion entirely - there is plenty of evidence that this is more of a repeated fable rather than accepted scientific and field experience, I am pained to give more light to this and would question what it brings to this otherwise well considered study. A quick survey of many beaver dams proves they have the sound of running water - and if not this can change over the course of a day / week or weather condition and many other damming triggering behaviours are widely reported in the scientific literature

**RESPONSE:** Reviewer #1 also suggested deleting this section. Since two reviewers had the same opinion, I followed their recommendation. I also removed associated literature citations from the reference list.

Line 634 paragraph - feel this is the place to discuss the practicalities and animal welfare considerations of releasing beavers into such regions, wider population density, release site fidelity etc

**RESPONSE:** I am surprised by this recommendation because I feel that the last paragraph of the Discussion does deal with these issues. I argue against “rashly rushing to translocate beaver to tidal marsh systems”, for example. I mention uncertainty about carrying capacity (≈ “population density”) and juvenile dispersal (≈ “release site fidelity), and the importance of understanding landscape connectivity for beaver movement to and from estuarine habitat (≈ “release site fidelity). And I mention all of these points as research gaps.

**Reviewer #3 (Ruairidh D. Campbell)**

This ms presents interesting data on the form and distribution of beaver dams in tidal habitats. Some of the data is descriptive. However, **overall I found the paper to lack focus** and therefore it was difficult to follow at times. **Probably what is needed is for a tight set of aims** (perhaps with hypotheses, but not necessarily so), with the results and discussion structured around these. I also couldn't find the data on OSF despite the author saying it was there, so this needs to be checked. I think the data could benefit from some additional statistical modelling to better understand the pattern of dam building and lodge construction in these environments. There is a good dataset here that deserves to be used. Some of the discussion near the end could be tightened up too. I've put a few specific comments below. I hope this gets resubmitted as there is very little in the literature on the subject of beavers occupying tidal areas.

**RESPONSE:** In this response I am focusing on the call for a tighter set of aims in the Introduction, because the other comments in this paragraph are expounded on in greater detail in subsequent comments by the reviewer. The issue of greater clarity in the goals of the paper was also mentioned by reviewer #1. I have edited the Introduction to clarify the structure of the paper and the goals of each structural component. I have also edited the Discussion to circle back on the goals described in the Introduction.

Lines 47-51. My immediate question here is whether tidal beaver dams act as a fish trap during receding tides, rather than being ‘chosen’ by fish as refuges during low tide. Clearly, these juvenile salmon like these tidal channels, otherwise they wouldn’t move up there in the first place. But, though out with the scope of this study, understanding the effects of these dams on juvenile salmon habitat selection, mortality, growth rates and other life history characteristics would be very interesting. My main point in commenting on this is that unless one conducts the above research, we can’t be certain these beaver pools ‘support’ triple the density rather than just happen to hold this greater density as a result of 'entrapment' of individuals moving in and out of the creeks with the tidal fluctuations, so I suggest using the word ‘hold’ in place of ‘support’ in line 48.

**RESPONSE:** I have changed the wording from “support” to “contain”. Later, in the Results and Discussion, I note that the dams flood at high tide allowing fish to pass. Better understanding the role of low-tide dam pools on fish is an avenue of research I intend to pursue.

Line 139. Please provide the full name on first mention of RTK-GPS.

**RESPONSE:** Reviewer #1 made the same comment. Changed as suggested.

Lines 138-148. Please provide the dates (years and months) of the various parts of the field work.

**RESPONSE:** Months and years added as requested.

Lines 220-222 (Table 2). I don’t recall any description of how the literature review was conducted. Is this representative sample of literature on dam frequency?

**RESPONSE:** There was no description of the literature review because it was simply a routine search of Google Scholar using key words such as “beaver” and “density”, “dam density”, “colony density”. I included data in the table from all of the relevant papers that I could find. When I have seen other papers with similar tables collating data and citing the data sources, I have never seen a detailed methods description of the literature source. It is simply taken for granted that the author did as good a search for relevant literature as they could. On the other hand, meta-analyses typically do have descriptions of the literature search, but I don’t consider what I did a meta-analysis. I will note that the other two reviewers (and #1 was a stickler) did not mention this issue.

I also find it interesting that the damming frequency is lower in fluvial and lacustrine environments (and no individual study as high as this one), because I imagine the range of stream gradients would be higher away from intertidal areas. But then again, perhaps intertidal dams are smaller (maybe due to constraints from the bidirectional flow) and so more are needed. I hope you discuss this later.

**RESPONSE:** I am reluctant to speculate on the potential difference in damming frequency between fluvial and tidal systems for two reasons: [1] my sample size of tidal systems is not large, so sampling of a greater variety of coastal systems (see Fig 9) is likely to broaden the range of damming frequencies, and [2] there is quite a lot of variation in fluvial damming frequency, and it is not at all clear why. However, I do suggest in two different locations of the Discussion possible reasons for a series of dams rather than one or two dams: risk of dam failure from high water pressure (head), and possible water freshening from dams acting as salinity barriers.

**Lines 226-233. Ideally one would compare mound lodge locations with a random set of bank-side points and us a binomial model to analyse whether the selection of the various channel types (blind, distributary or main channel?) and distance to distributary junctions / confluence was statistically significant.**

**RESPONSE:** This is a great idea. I have done this, but I use G-tests to compare the frequency distributions of beaver movement routes from observed lodge locations versus random locations. I also added an additional level of comparison to the 15-m search criterion, by searching for all possible movement routes within 50 m of a lodge (or random point) location. This was done to assure that the results were consistent over several scales and not an artifact of a single scale. Of course, at extreme scales there should be no difference between random and observed (e.g., 1-m or 1000-m scales). Finally, I compared movement route counts for lodges on distributary banks vs. those on blind tidal channel banks. I rewrote the associated text to indicate that the results suggest that beaver bias the siting of their lodges towards locations with high channel network connectivity, or many options for movement routes. The stark differences in movement routes between distributary and blind tidal channel locations suggests possible trade-offs between these two locations. The methods were also updated to reflect these changes.

Lines 231-233 and Fig 4. Was the difference in frequency distribution statistically significant. I think a non-parametric test comparing these (e.g. KS or Mann-Whitney U) would be possible here.

**RESPONSE:** See the previous response (Lines 226-233). I used G-tests to compare frequency distributions.

Table 3. As my comment on Table 2.

**RESPONSE:** Reply same as for Table 2, except that the routine search of Google Scholar used the key words “beaver” and “dam geometry”, “dam height”, “dam head”, dam pool/pond depth”, etc. I included data in the table from all of the relevant papers that I could find.

Lines 335-340. Curious. Were shorter channels also of higher gradient? How did the water heads compare in shorter and longer channels?

**RESPONSE:** I calculated gradients and elaborated on the previous regression analysis, adding two more graphs to the figure (and two graphs to the supplementary materials). Consequently, I extensively edited the accompanying text.

Table 4. I find this initially a little confusing, so I think this needs a short explanation in the table caption or change in the column/row labels. ‘Predicted’ means identified or otherwise from aerial photographs and ‘observed’ is ‘ground-truthed’ data.

**RESPONSE:** A short explanation was added to the table caption, as suggested.

Table 5. This is also confusing, despite the footnote (which I initially missed anyway). Could you change the wording? Success is a true positive? Fail is a false positive? Missed dam is a false negative?

**RESPONSE:** I have changed the wording as suggested and moved it from the footnote to the Table caption.

Line 530. I think clearer wording needed: not all readers might understand colloquialisms such as “homebodies”. Perhaps just say they have reduced home ranges.

**RESPONSE:** Changed as suggested.

To ensure your figures meet our technical requirements, please review our figure guidelines: <https://journals.plos.org/plosone/s/figures>

You may also use PLOS’s free figure tool, NAAS, to help you prepare publication quality figures: <https://journals.plos.org/plosone/s/figures#loc-tools-for-figure-preparation>.
