# Supplementary material for: Beaver in tidal habitat: Examples from the Pacific Northwest
Source: PLoS One. 2026 Jul 8;21(7):e0349313. doi: 10.1371/journal.pone.0349313 (PMC13345279; doi:10.1371/journal.pone.0349313)
Supplement: Attachment — Submitted filename: Response to Reviewers 22Apr2026.docx [file pone.0349313.s004.docx]

Responses to Reviewers and Editor (part 2)

As you can see, both reviewers were largely satisfied with the corrections you did to the manuscript and convinced it now is close to a state in which it could be published. One of them nevertheless asks for a

**[Reviewer] clarification regarding figure 7 and what the notations NF blind, NF distrib stand for**

**I made two changes to address this recommendation: [1] I added a parenthetical “(NF)” after the text “North Fork” in the manuscript figure legend (see track changes) to show that “North Fork” was being abbreviated to “NF”, and [2] I edited the legend box in the top figure changing “blind” to “tidal” to be consistent with the rest of the text, as the term “blind channel” was changed to merely “tidal” channel in response to previous recommendations by reviewers—I simply missed making the change in this figure; I also fully spelled out “distributary” to replace to previous abbreviation “distrib”.**

and the other one, while acknowledging that the manuscript is very well written, **[Reviewer] suggestion that the editing could still be a bit improved, especially by making the research goals clearer.**

**[Editor] I also agree that some minor editing and attention to keep the flow of the text focused on the research question might help "holding the hand" of the reader along this really nice study.**

**I have addressed these concerns by making edits in two locations in the manuscript. The first section of new text occurs from line 84 to 91 and consists of the following new text (underlined and blue) with original text in black and mostly removed. I believe this first section more explicitly details the goals of the paper, as requested.**

“The goal of this focus is to explore spatial patterns in dam and lodge locations (a) to provide insight into the abundance of beaver structures and their distribution relative to estuarine habitats, e.g., characterized by vegetation communities and channel networks; (b) to allow comparison with fluvial beaver, e.g., are there significant differences in dam or lodge density that might suggest differences in habitat quality or beaver behavior; (c) to understand the function of dams for beaver in tidal environments and how that compares with fluvial dams; and (d) to contribute guidance for tidal wetland restoration design that accommodates beaver and the ecosystem functions they may provide. ~~infer the role of beaver dams in tidal systems, from the perspective of beaver, and contrast it with their role in fluvial systems. An additional contrast is how beaver choose lodge locations in a two-dimensional estuarine landscape compared to a mostly one-dimensional riverine landscape.”~~

**In a second location, from line 367 to 374, I added a paragraph (see text below) that describes a hypothesis that provides context for the results described in subsequent paragraphs and in Fig. 7. In three locations in the results, I also added phrases that indicated consistency or inconsistency with the hypothesis.**

“It is reasonable to think that channel size could affect beaver dam construction. If we have a simplistic hypothesis that assumes tidal dams are generally relatively uniform in design (with some variation around a mean), then each dam would have a similar height and water head, so that a nearly constant number of dams would be found in tidal channels regardless of channel size (length), because the difference between low tide channel bottom elevations and MHHW is similar for almost all channels (except small tributaries to tidal channels). If true, then short channels should have steeper gradient and closely spaced dams while long tidal channels should have lower gradient and more widely spaced dams.”
