# Supplementary material for: Beaver in tidal habitat: Examples from the Pacific Northwest
Source: PLoS One. 2026 Jul 8;21(7):e0349313. doi: 10.1371/journal.pone.0349313 (PMC13345279; doi:10.1371/journal.pone.0349313)
Supplement: Peer review history

# Peer review: Decision Letter 0

**DOI:** 10.1371/journal.pone.0349313.r001

**Authors:** Sarah Jose

7 Jan 2026

PONE-D-25-54787

Beaver in tidal habitat: Examples from the Pacific Northwest

PLOS One

Dear Dr. Hood,

Thank you for submitting your manuscript to PLOS ONE. After careful consideration, we feel that it has merit but does not fully meet PLOS ONE’s publication criteria as it currently stands. Therefore, we invite you to submit a revised version of the manuscript that addresses the points raised during the review process.

Please carefully review the feedback received from the three valued reviewers and revise your manuscript to address their comments, providing a point-by-point response to the reviewers upon resubmission.

Please submit your revised manuscript by Feb 20 2026 11:59PM. If you will need more time than this to complete your revisions, please reply to this message or contact the journal office at plosone@plos.org. When you’re ready to submit your revision, log on to https://www.editorialmanager.com/pone/ and select the ‘Submissions Needing Revision’ folder to locate your manuscript file.

Please include the following items when submitting your revised manuscript:

- A letter that responds to each point raised by the academic editor and reviewer(s). You should upload this letter as a separate file labeled ‘Response to Reviewers’.
- A marked-up copy of your manuscript that highlights changes made to the original version. You should upload this as a separate file labeled ‘Revised Manuscript with Track Changes’.
- An unmarked version of your revised paper without tracked changes. You should upload this as a separate file labeled ‘Manuscript’.

If you would like to make changes to your financial disclosure, please include your updated statement in your cover letter. Guidelines for resubmitting your figure files are available below the reviewer comments at the end of this letter.

If applicable, we recommend that you deposit your laboratory protocols in protocols.io to enhance the reproducibility of your results. Protocols.io assigns your protocol its own identifier (DOI) so that it can be cited independently in the future. For instructions see: https://journals.plos.org/plosone/s/submission-guidelines#loc-laboratory-protocols. Additionally, PLOS ONE offers an option for publishing peer-reviewed Lab Protocol articles, which describe protocols hosted on protocols.io. Read more information on sharing protocols at https://plos.org/protocols?utm_medium=editorial-email&utm_source=authorletters&utm_campaign=protocols.

We look forward to receiving your revised manuscript.

Kind regards,

Sarah Jose, Ph.D.

Staff Editor

PLOS One

Journal requirements:

When submitting your revision, we need you to address these additional requirements.

1. Please ensure that your manuscript meets PLOS ONE’s style requirements, including those for file naming. The PLOS ONE style templates can be found at

https://journals.plos.org/plosone/s/file?id=wjVg/PLOSOne_formatting_sample_main_body.pdf and

https://journals.plos.org/plosone/s/file?id=ba62/PLOSOne_formatting_sample_title_authors_affiliations.pdf

2. Please note that PLOS One has specific guidelines on code sharing for submissions in which author-generated code underpins the findings in the manuscript. In these cases, we expect all author-generated code to be made available without restrictions upon publication of the work. Please review our guidelines at https://journals.plos.org/plosone/s/materials-and-software-sharing#loc-sharing-code and ensure that your code is shared in a way that follows best practice and facilitates reproducibility and reuse.

3. When completing the data availability statement of the submission form, you indicated that you will make your data available on acceptance. We strongly recommend all authors decide on a data sharing plan before acceptance, as the process can be lengthy and hold up publication timelines. Please note that, though access restrictions are acceptable now, your entire data will need to be made freely accessible if your manuscript is accepted for publication. This policy applies to all data except where public deposition would breach compliance with the protocol approved by your research ethics board. If you are unable to adhere to our open data policy, please kindly revise your statement to explain your reasoning and we will seek the editor’s input on an exemption. Please be assured that, once you have provided your new statement, the assessment of your exemption will not hold up the peer review process.

4. We note that Figures 1, 2, 3, 5, 6, 7, 9, 10, 11,               S1, S10, S11, S12, S13, S14, S15, S16, S17, S18, S19, S20, S21, S22, S23, S24, S25, S26, S2, S28, S29, S30, S31, S32, S33, S34, S36, S37, and S39 in your submission contain [map/satellite] images which may be copyrighted. All PLOS content is published under the Creative Commons Attribution License (CC BY 4.0), which means that the manuscript, images, and Supporting Information files will be freely available online, and any third party is permitted to access, download, copy, distribute, and use these materials in any way, even commercially, with proper attribution. For these reasons, we cannot publish previously copyrighted maps or satellite images created using proprietary data, such as Google software (Google Maps, Street View, and Earth). For more information, see our copyright guidelines: http://journals.plos.org/plosone/s/licenses-and-copyright.

We require you to either (1) present written permission from the copyright holder to publish these figures specifically under the CC BY 4.0 license, or (2) remove the figures from your submission:

1. You may seek permission from the original copyright holder of Figures 1, 2, 3, 5, 6, 7, 9, 10, 11, S1, S10, S11, S12, S13, S14, S15, S16, S17, S18, S19, S20, S21, S22, S23, S24, S25, S26, S2, S28, S29, S30, S31, S32, S33, S34, S36, S37, and S39 to publish the content specifically under the CC BY 4.0 license.

We recommend that you contact the original copyright holder with the Content Permission Form (http://journals.plos.org/plosone/s/file?id=7c09/content-permission-form.pdf) and the following text:

“I request permission for the open-access journal PLOS ONE to publish XXX under the Creative Commons Attribution License (CCAL) CC BY 4.0 (http://creativecommons.org/licenses/by/4.0/). Please be aware that this license allows unrestricted use and distribution, even commercially, by third parties. Please reply and provide explicit written permission to publish XXX under a CC BY license and complete the attached form.”

Please upload the completed Content Permission Form or other proof of granted permissions as an "Other" file with your submission.

In the figure caption of the copyrighted figure, please include the following text: “Reprinted from [ref] under a CC BY license, with permission from [name of publisher], original copyright [original copyright year].”

2. If you are unable to obtain permission from the original copyright holder to publish these figures under the CC BY 4.0 license or if the copyright holder’s requirements are incompatible with the CC BY 4.0 license, please either i) remove the figure or ii) supply a replacement figure that complies with the CC BY 4.0 license. Please check copyright information on all replacement figures and update the figure caption with source information. If applicable, please specify in the figure caption text when a figure is similar but not identical to the original image and is therefore for illustrative purposes only.

The following resources for replacing copyrighted map figures may be helpful:

USGS National Map Viewer (public domain): http://viewer.nationalmap.gov/viewer/

The Gateway to Astronaut Photography of Earth (public domain): http://eol.jsc.nasa.gov/sseop/clickmap/

Maps at the CIA (public domain): https://www.cia.gov/library/publications/the-world-factbook/index.html and https://www.cia.gov/library/publications/cia-maps-publications/index.html

NASA Earth Observatory (public domain): http://earthobservatory.nasa.gov/

Landsat: http://landsat.visibleearth.nasa.gov/

USGS EROS (Earth Resources Observatory and Science (EROS) Center) (public domain): http://eros.usgs.gov/#

Natural Earth (public domain): http://www.naturalearthdata.com/

If the reviewer comments include a recommendation to cite specific previously published works, please review and evaluate these publications to determine whether they are relevant and should be cited. There is no requirement to cite these works unless the editor has indicated otherwise.

[Note: HTML markup is below. Please do not edit.]

Reviewers’ comments:

Reviewer’s Responses to Questions

**Comments to the Author**

1. Is the manuscript technically sound, and do the data support the conclusions?

The manuscript must describe a technically sound piece of scientific research with data that supports the conclusions. Experiments must have been conducted rigorously, with appropriate controls, replication, and sample sizes. The conclusions must be drawn appropriately based on the data presented.

Reviewer #1: Partly

Reviewer #2: Yes

Reviewer #3: Partly

**********

2. Has the statistical analysis been performed appropriately and rigorously?

Reviewer #1: Yes

Reviewer #2: Yes

Reviewer #3: Yes

**********

3. Have the authors made all data underlying the findings in their manuscript fully available?

The PLOS Data policy requires authors to make all data underlying the findings described in their manuscript fully available without restriction, with rare exception (please refer to the Data Availability Statement in the manuscript PDF file). The data should be provided as part of the manuscript or its supporting information, or deposited to a public repository. For example, in addition to summary statistics, the data points behind means, medians and variance measures should be available. If there are restrictions on publicly sharing data—e.g. participant privacy or use of data from a third party—those must be specified.

Reviewer #1: No

Reviewer #2: Yes

Reviewer #3: No

**********

4. Is the manuscript presented in an intelligible fashion and written in standard English?

PLOS ONE does not copyedit accepted manuscripts, so the language in submitted articles must be clear, correct, and unambiguous. Any typographical or grammatical errors should be corrected at revision, so please note any specific errors here.

Reviewer #1: No

Reviewer #2: Yes

Reviewer #3: Yes

**********

5. Review Comments to the Author

Please use the space provided to explain your answers to the questions above. You may also include additional comments for the author, including concerns about dual publication, research ethics, or publication ethics. (Please upload your review as an attachment if it exceeds 20,000 characters)

Reviewer #1: This is a very interesting journal article where the author demonstrates significant knowledge about the system and includes an impressive amount of fieldwork, documenting beavers pushing into marine habitats, something that can significantly extend the knowledge and understanding of beavers. I want to congratulate the author on recognizing this pattern and aiming to publish this important contribution!

Unfortunately, in its current form, the manuscript suffers from a lack of clear research questions, clarity in the result and method section and fails to highlight the potential implications of this paper (which are definitely there). I think with some work, especially on introduction, presentation of the results and discussion, these limitations can be addressed, and this paper can fulfil its full potential to an international readership.

Especially crucial is to rework the result section. Full of jargon and site names, I got lost a lot trying to understand where we are and what is being talked about. For a primarily freshwater ecologist (which most beaver people are), this was challenging, and marine terms and context need a much better introduction.

In my opinion, the author has to decide where this story should be published and in what format. If it is to be published in an internation journal with the aim to put the idea out that beavers also live in tidal habitats, then the manuscript needs to be trimmed and generalized for the internation reader. If the author would like to tell a more detailed story including discussing site specifics, then a local naturalist journal might be the better choice.

Data availability

Where is the data available? Please be more specific, ideally with a link.

Abstract

Line 22 Please spell out or remove the abbreviation RTK

Line 24 Please mention density per km

Introduction

General comments

Nice introduction with very good sections. Some parts can be shortened/condensed (BDA’s, part on fish from Line 51). Given this has the ambition to be a naturalist paper informing the international readership about the extraordinary capabilities of beavers extending into tidal marches, I would like some more description of the habitat and beaver biology compared to the more standard beaver freshwater stream habitat. Some of that information may be in the methods already, I would consider moving it. This will help the reader understand why beaver colonize these habitats and ultimately also how that could be applied to other areas.

Additionally, from the current introduction, the research questions are not so clear to me and that could be achieved with a better structuring of the introduction clearly highlighting knowledge gaps.

Finally, I miss an overview or at least the question of other places where beavers push into saltwater/marine habitats. Is this know from other places than the Pacific Northwest? I think some literature is available. I think also beavers in Argentina were known to colonize islands separated by saltwater and feed on marine food like algae. Check Anderson et al. 2008 Mammal review doi: 10.1111/j.1365-2907.2008.00136.x as a starter.

Line 35 please specify where each beaver is native (broadly) and which beaver species is dealt with in this manuscript

Line 60 I am not sure why suddenly the text is about BDA’s so much. I understand that there seems to be a management/knowledge gap, but I am not sure the specific examples (maybe limit to one) add much for the international reader. Maybe that is also something for the discussion?

Line 65 As mentioned in the comment below, I would expand a little bit the physical differences of tidal ecosystems to more linear stream ecosystems. Maybe also worth to expand more generally about differences in biology of the beaver (why is it in the tidal marshes, what is it building dams out of, what is it feeding on)

Line 70 From here on, I got confused. The first sentence is clear, but then I feel the last sentence might be more appropriate and I also do not understand why the small stream are inhospitable.

Maybe a broader introduction into the dynamics of tidal habitats could help follow the reader here. The sentence from line 71 I would move to the end and start with something like “to do this, we choose the following study regions representing ….”

Methods

Whatever you decide to do with the results, follow the same logic in the methods.

I think a simple map, or at least a better description where these sites are and how much they are separated would help the reader. I am not even sure the country has been mentioned. I am generally confused by the many localities mentioned and specific histories that I am not sure are important for the paper’s questions.

Line 139 Spell out abbreviation for RTK

Results

The result need a lot of restructuring. Currently, this is not publishable, as it is hard to follow and often lost in details. I tried to suggest improvements, because I think the manuscript idea is great!

I suggest 3-4 Figures, in total one per section.

Whitin the sections:

- Separate your results better. Answer the main questions.

- A lot of the site-specifics has to go to help the reader better follow. Consider to elaborate and describe site specifics in the appendix. The results need to be generalized (at least the text).

Maybe have two section types. First a section of the dams and transects (things you measured), then a section about naturalist history, eg how the dams are build (things you observed).

I think some of these Figure S1-S9 can go into a composite figure in the main manuscript to illustrate the habitat.

In turn, I would reduce the figures in the main manuscript (or do a composite figure) for Figures 1-6

Line 160 I do not understand how this 2000 m were selected, at random, is this everything or accessibility? This should be mentioned in the methods. Again I am a bit at loss which name refers to which area and a map and a clear structure/separation in methods results per area could definitely help. Also for the other areas, data about the size belongs to methods in my opinion.

Line 162 to line 170 I would just mention in the results that due to challenging field conditions only half of the terrain could be sampled. The speculation on line 170 belongs into the discussion, same with line 174 and line 179.

Line 213 I am sorry, but I cannot follow this sentence. What is lodges per colony? Not even “colony” has been introduced as a term. While this might be clear for beaver people, it could create confusion for other readers. Also what does it has to do with Table 2?

Line 220 I suggest to move this table to supplementary materials. I like that you back up your claims with data, but would generally advice to reduce numbers of figures and tables in the main story to focus on the most important contributions.

Line 223 First it was not clear to me that now lodges are reported. Maybe here (and in other places) separate sub-headings could help to guide the reader?

Line 235 It is not entirely clear to me what a blind or a distributary channel is, this has not been introduced. Best place probably in the methods. Same with movement routes, this not be properly defined. What movement is it? Water, beaver? What is a route count?

Line 246 Maybe it helps to add to larger sampling region (here Snohomish) so the reader keep some simpler overview of the sites

Line 255 What is the mean higher high water? Keep in mind, beaver people are often not very knowledgeable in marine jargon, you need to explain these things much better. Probably I miss some terms as well in this review, check again that you explained all terms very well. At this point I think a glossary could be helpful!

Additionally: What is navd88? Is it needed in the figure legend?

Also clearly label which graph belongs to which line on the figure

Apply these changes to all figures, of course.

Line 269 Why is there two red dots in the figure shown when it mentions: Other channel trace profile points are not shown to avoid graphic clutter

Line 280, from “probably”: This is discussion up until the “provides an example” Also again from 297. I currently do not understand what the added value of the extensive description of this (seemingly) separate case study is. What does the reader learn that is important for the main story? Maybe some of this natural history and curiosity (which has its value!) can go in the description of the Fig S9?

Line 327 I would separate the table and pit the literature results for freshwater into the appendix, and summarize them in text. Why has N different numbers, is this the sample size per “stream”?

Section starting from Line 340: Always use p < 0.001 if p-value is very small

Line 358 This could be the best figure of your manuscript, but currently I do not understand what is going on. Why is Snohomisch differet to NF blind (what is NF) and NF distrib? What are the different regression lines for and why do the y differ from the ones in the text? I would suggest: Simplify the figure, show one regression per figure (overall). Show site-specific regressions in the appendix and condition specific regression only on the main text if they are significant.

Line 370 I find it hard to understand to follow this error matrix. Can you add a sentence that helps how to read it? For example: “reading example” In the North Fork area we predicted 121 dams, whereas we observed 69…” I would also move this table to the appendix and add an “overall” category

Line 387 That is partly not results, but discussion

Line 393: I would suggest t move that into the appendix

Line 399 That is partly methods. I think you do not mention this part in the methods at all?

Line 422 That is a very cool result and overview!

Line 425 That is discussion from here

Line 442 Another candidate for the supplementary materials

Line 458 That is the sort of detail that can be probably safely removed from the manuscript without loosing too much knowledge. Same with the paragraph 460 onwards (maybe one sentence in the discussion)

Discussion

In general, the discussion is too long, often too far away from what the actual research topic was (a lot of speculation and open questions not backed up by the data) and lacking integration into other research. A lot of interesting points are there, but buried in the text. Again I would emphasize not to use site names (only exceptionally), but instead generalize. If people are interested in specific sites, they can still contact the author, but for most international readers the local site names do not matter.

Line 493 Interesting, can you elaborate a bit more what you mean with freshwater habitat?

Line 526 Lot’s of individual site names again in this paragraph, maybe it is possible to reduce these? Here you are trying to generalize, not to discuss individual cases. Maybe saying “some sites have high molbility, some low” is therefore sufficient.

Line 535 I would avoid questions and frame it as research gaps: “It is unclear why”

Line 573 Again, I would avoid questions and frame it as research gaps: “It is unclear why”

Line 598 For me that whole paragraph is speculation and has nothing to do with the presented data (no sound measurments done, not introduced in the introduction). It is interesting, but for me needs another study. Maybe keep 1-2 sentences.

Line 625 For me, also these parts are discussed to much being to far from the research topic. If I am not mistake the increased fish densities were mentioned earlier already, so maybe there 1-2 points can be taken up

Reviewer #2: This is a well written piece and very interesting topic, though potentially to a limited audience, it still supports further knowledge expansion regarding a highly popular species of conservation and active restoration interest. I suggest minor revisions to improve readability.

Personally, suggest any rewriting of any first-person statements eg lines 53-55

Any further exploration of Eurasian beavers in tidal zones, we certainly witness this but agree little documentation

Line 57 reintroducing rather than introducing for correct conservation biology term - surely beavers here historically

Detailed methodology and a range of variables discussed though I think I’m missing more on the wider beaver population density and history on when how why beavers have entered this system, I guess we presume it is population density pressure pushing beavers to live in theoretically more challenging and ‘sub optimal’ areas - are you seeing this as a result of this - therefore would releases into such areas (as briefly proposed) without other habitat infilling just result in released animals moving inland to ‘easier’ habitat and therefore release site fidelity would be challenging for conservation projects and a nice link for BDA investigations as pointed out - perhaps expand this point? Personally, I’m interested in how well are the beavers doing in these areas - any insight into body conditions, breeding stats etc?

Good use of tables to summarise results as some of the text can be a bit dense to read and take in - I just wonder is there any scope to put any more of the local scale field survey results into a table - just a question - like habitat/ forage type access?

Suggest there is repetition eg use of aerial photos in the regional scale section - could be more concise here along with discussion lines 545-569 - please consider condensing

Discussion - sound of water - I would consider this inclusion entirely - there is plenty of evidence that this is more of a repeated fable rather than accepted scientific and field experience, I am pained to give more light to this and would question what it brings to this otherwise well considered study. A quick survey of many beaver dams proves they have the sound of running water - and if not this can change over the course of a day / week or weather condition and many other damming triggering behaviours are widely reported in the scientific literature

Line 634 paragraph - feel this is the place to discuss the practicalities and animal welfare considerations of releasing beavers into such regions, wider population density, release site fidelity etc

Reviewer #3: This ms presents interesting data on the form and distribution of beaver dams in tidal habitats. Some of the data is descriptive. However, overall I found the paper to lack focus and therefore it was difficult to follow at times. Probably what is needed is for a tight set of aims (perhaps with hypotheses, but not necessarily so), with the results and discussion structured around these. I also couldn’t find the data on OSF despite the author saying it was there, so this needs to be checked. I think the data could benefit from some additional statistical modelling to better understand the pattern of dam building and lodge construction in these environments. There is a good dataset here that deserves to be used. Some of the discussion near the end could be tightened up too. I’ve put a few specific comments below. I hope this gets resubmitted as there is very little in the literature on the subject of beavers occupying tidal areas.

Lines 47-51. My immediate question here is whether tidal beaver dams act as a fish trap during receding tides, rather than being ‘chosen’ by fish as refuges during low tide. Clearly, these juvenile salmon like these tidal channels, otherwise they wouldn’t move up there in the first place. But, though out with the scope of this study, understanding the effects of these dams on juvenile salmon habitat selection, mortality, growth rates and other life history characteristics would be very interesting. My main point in commenting on this is that unless one conducts the above research, we can’t be certain these beaver pools ‘support’ triple the density rather than just happen to hold this greater density as a result of ‘entrapment’ of individuals moving in and out of the creeks with the tidal fluctuations, so I suggest using the word ‘hold’ in place of ‘support’ in line 48.

Line 139. Please provide the full name on first mention of RTK-GPS.

Lines 138-148. Please provide the dates (years and months) of the various parts of the field work.

Lines 220-222 (Table 2). I don’t recall any description of how the literature review was conducted. Is this representative sample of literature on dam frequency? I also find it interesting that the damming frequency is lower in fluvial and lacustrine environments (and no individual study as high as this one), because I imagine the range of stream gradients would be higher away from intertidal areas. But then again, perhaps intertidal dams are smaller (maybe due to constraints from the bidirectional flow) and so more are needed. I hope you discuss this later.

Lines 226-233. Ideally one would compare mound lodge locations with a random set of bank-side points and us a binomial model to analyse whether the selection of the various channel types (blind, distributary or main channel?) and distance to distributary junctions / confluence was statistically significant.

Lines 231-233 and Fig 4. Was the difference in frequency distribution statistically significant. I think a non-parametric test comparing these (e.g. KS or Mann-Whitney U) would be possible here.

Table 3. As my comment on Table 2.

Lines 335-340. Curious. Were shorter channels also of higher gradient? How did the water heads compare in shorter and longer channels?

Table 4. I find this initially a little confusing, so I think this needs a short explanation in the table caption or change in the column/row labels. ‘Predicted’ means identified or otherwise from aerial photographs and ‘observed’ is ‘ground-truthed’ data.

Table 5. This is also confusing, despite the footnote (which I initially missed anyway). Could you change the wording? Success is a true positive? Fail is a false positive? Missed dam is a false negative?

Line 530. I think clearer wording needed: not all readers might understand colloquialisms such as “homebodies”. Perhaps just say they have reduced home ranges.

**********

6. PLOS authors have the option to publish the peer review history of their article (what does this mean?). If published, this will include your full peer review and any attached files.

If you choose “no”, your identity will remain anonymous but your review may still be made public.

**Do you want your identity to be public for this peer review?**   For information about this choice, including consent withdrawal, please see our Privacy Policy.

Reviewer #1: No

Reviewer #2: No

Reviewer #3: **Yes:**  Ruairidh D. Campbell

**********

[NOTE: If reviewer comments were submitted as an attachment file, they will be attached to this email and accessible via the submission site. Please log into your account, locate the manuscript record, and check for the action link "View Attachments". If this link does not appear, there are no attachment files.]

To ensure your figures meet our technical requirements, please review our figure guidelines: https://journals.plos.org/plosone/s/figures

You may also use PLOS’s free figure tool, NAAS, to help you prepare publication quality figures: https://journals.plos.org/plosone/s/figures#loc-tools-for-figure-preparation.

NAAS will assess whether your figures meet our technical requirements by comparing each figure against our figure specifications.

# Author comment: Author response to Decision Letter 1

**DOI:** 10.1371/journal.pone.0349313.r002

6 Mar 2026

My responses to the editor and reviewers are collected in the attached file "Response to Reviewers". I would only add that I appreciate the constructive criticism, and the positive feedback and encouragement from the reviewers.

It would be easiest to look at the above referenced docx file, but it you want all of my responses included here, I have copied and pasted the docx text below. The copied text is not rich text so it misses out on bolding and text color that I use to distinguish reviewers’ and editors comments from my responses.

4. We note that Figures 1, 2, 3, 5, 6, 7, 9, 10, 11, S1, S10, S11, S12, S13, S14, S15, S16, S17, S18, S19, S20, S21, S22, S23, S24, S25, S26, S2, S28, S29, S30, S31, S32, S33, S34, S36, S37, and S39 in your submission contain [map/satellite] images which may be copyrighted. All PLOS content is published under the Creative Commons Attribution License (CC BY 4.0), which means that the manuscript, images, and Supporting Information files will be freely available online, and any third party is permitted to access, download, copy, distribute, and use these materials in any way, even commercially, with proper attribution. For these reasons, we cannot publish previously copyrighted maps or satellite images created using proprietary data, such as Google software (Google Maps, Street View, and Earth). For more information, see our copyright guidelines: http://journals.plos.org/plosone/s/licenses-and-copyright.

We require you to either (1) present written permission from the copyright holder to publish these figures specifically under the CC BY 4.0 license, or (2) remove the figures from your submission:

1. You may seek permission from the original copyright holder of Figures 1, 2, 3, 5, 6, 7, 9, 10, 11, S1, S10, S11, S12, S13, S14, S15, S16, S17, S18, S19, S20, S21, S22, S23, S24, S25, S26, S2, S28, S29, S30, S31, S32, S33, S34, S36, S37, and S39 to publish the content specifically under the CC BY 4.0 license.

We recommend that you contact the original copyright holder with the Content Permission Form (http://journals.plos.org/plosone/s/file?id=7c09/content-permission-form.pdf) and the following text:

“I request permission for the open-access journal PLOS ONE to publish XXX under the Creative Commons Attribution License (CCAL) CC BY 4.0 (http://creativecommons.org/licenses/by/4.0/). Please be aware that this license allows unrestricted use and distribution, even commercially, by third parties. Please reply and provide explicit written permission to publish XXX under a CC BY license and complete the attached form.”

Please upload the completed Content Permission Form or other proof of granted permissions as an "Other" file with your submission.

Author’s Response:

I have consulted the copyright acknowledgement requirements for Google, ESRI, and Pictometry (Eagleview Inc.), the sources the aerial imagery in my figures. None of them require requests for permission and Google specifically says that they do not have time to respond to such requests. Instead, they provide clear guidance for when their imagery can be use and how to acknowledge their copyright. I have detailed my response regarding content permissions in an attached “Other” file for each source of imagery.

In the figure caption of the copyrighted figure, please include the following text: “Reprinted from [ref] under a CC BY license, with permission from [name of publisher], original copyright [original copyright year].”

[Note: HTML markup is below. Please do not edit.]

Reviewers’ comments:

Reviewer’s Responses to Questions

Comments to the Author

1. Is the manuscript technically sound, and do the data support the conclusions?

The manuscript must describe a technically sound piece of scientific research with data that supports the conclusions. Experiments must have been conducted rigorously, with appropriate controls, replication, and sample sizes. The conclusions must be drawn appropriately based on the data presented.

Reviewer #1: Partly

Reviewer #2: Yes

Reviewer #3: Partly

________________________________________

2. Has the statistical analysis been performed appropriately and rigorously?

Reviewer #1: Yes

Reviewer #2: Yes

Reviewer #3: Yes

________________________________________

3. Have the authors made all data underlying the findings in their manuscript fully available?

The PLOS Data policy requires authors to make all data underlying the findings described in their manuscript fully available without restriction, with rare exception (please refer to the Data Availability Statement in the manuscript PDF file). The data should be provided as part of the manuscript or its supporting information, or deposited to a public repository. For example, in addition to summary statistics, the data points behind means, medians and variance measures should be available. If there are restrictions on publicly sharing data—e.g. participant privacy or use of data from a third party—those must be specified.

Reviewer #1: No

Reviewer #2: Yes

Reviewer #3: No

________________________________________

4. Is the manuscript presented in an intelligible fashion and written in standard English?

Reviewer #1: No

Reviewer #2: Yes

Reviewer #3: Yes

________________________________________

5. Review Comments to the Author

Please use the space provided to explain your answers to the questions above. You may also include additional comments for the author, including concerns about dual publication, research ethics, or publication ethics. (Please upload your review as an attachment if it exceeds 20,000 characters)

Reviewer #1:

This is a very interesting journal article where the author demonstrates significant knowledge about the system and includes an impressive amount of fieldwork, documenting beavers pushing into marine habitats, something that can significantly extend the knowledge and understanding of beavers. I want to congratulate the author on recognizing this pattern and aiming to publish this important contribution!

Unfortunately, in its current form, the manuscript suffers from a lack of clear research questions, clarity in the result and method section and fails to highlight the potential implications of this paper (which are definitely there). I think with some work, especially on introduction, presentation of the results and discussion, these limitations can be addressed, and this paper can fulfil its full potential to an international readership.

RESPONSE: I have clarified to research objectives of the paper in the Introduction and I have added a Summary after the Discussion to clarify the “take-home” messages of the paper and highlight the implications of the paper.

Especially crucial is to rework the result section. Full of jargon and site names, I got lost a lot trying to understand where we are and what is being talked about. For a primarily freshwater ecologist (which most beaver people are), this was challenging, and marine terms and context need a much better introduction.

RESPONSE: I added a paragraph in the Introduction and in the Study Areas section of the Methods to provide a brief primer on tidal systems, including necessary jargon. Unfortunately, this jargon is essential vocabulary for estuarine ecology.

In my opinion, the author has to decide where this story should be published and in what format. If it is to be published in an internation journal with the aim to put the idea out that beavers also live in tidal habitats, then the manuscript needs to be trimmed and generalized for the internation reader. If the author would like to tell a more detailed story including discussing site specifics, then a local naturalist journal might be the better choice.

Abstract

Line 22 Please spell out or remove the abbreviation RTK

RESPONSE: I spelled out the abbreviation.

Line 24 Please mention density per km

RESPONSE: Changed as suggested.

Introduction

General comments

Nice introduction with very good sections. Some parts can be shortened/condensed (BDA’s, part on fish from Line 51). Given this has the ambition to be a naturalist paper informing the international readership about the extraordinary capabilities of beavers extending into tidal marches, I would like some more description of the habitat and beaver biology compared to the more standard beaver freshwater stream habitat. Some of that information may be in the methods already, I would consider moving it. This will help the reader understand why beaver colonize these habitats and ultimately also how that could be applied to other areas.

RESPONSE: I added a paragraph to the Introduction that summarizes the principal differences between fluvial and tidal systems that beaver, and beaver biologists, must confront. I think this will be useful for beaver biologists who are typically unfamiliar with tidal ecosystems.

Additionally, from the current introduction, the research questions are not so clear to me and that could be achieved with a better structuring of the introduction clearly highlighting knowledge gaps.

RESPONSE: The issue of greater clarity in the goals of the paper was also mentioned by reviewer #3. I have edited the Introduction to clarify the structure of the paper and the goals of each structural component. I have also edited the Discussion to circle back on the goals described in the Introduction. With regard to highlighting knowledge gaps, there is almost nothing known about tidal beaver—almost everything is a knowledge gap (which I point out in the new edits), but I hope I have made it clear that I am addressing two particular gaps and pointing out the need for greater attention to tidal beaver in general.

Finally, I miss an overview or at least the question of other places where beavers push into saltwater/marine habitats. Is this know from other places than the Pacific Northwest? I think some literature is available. I think also beavers in Argentina were known to colonize islands separated by saltwater and feed on marine food like algae. Check Anderson et al. 2008 Mammal review doi: 10.1111/j.1365-2907.2008.00136.x as a starter.

RESPONSE: Yes, this story is well known. Beaver can swim through salty seas to disperse to islands or along shorelines. This has also been observed in Puget Sound. But dispersal is temporary transit. Occupation of estuarine (brackish) habitat is long-term and a much different phenomenon.

Line 35 please specify where each beaver is native (broadly) and which beaver species is dealt with in this manuscript.

RESPONSE: Parenthetical notes were made for each species at this point in the text. Given that the fieldwork in this manuscript takes place in North America, it did not seem necessary to explicitly say that this paper focused solely on C. canadensis—also there are literature-based comparisons of lodges and dams in a couple of tables that include both species, so to say that this paper is exclusively about C. canadensis is not precisely accurate. Both species have a lot in common, which is why I included C. fiber in the literature-based comparisons.

Line 60 I am not sure why suddenly the text is about BDA’s so much. I understand that there seems to be a management/knowledge gap, but I am not sure the specific examples (maybe limit to one) add much for the international reader. Maybe that is also something for the discussion?

RESPONSE: I have moved this text to the Discussion section dealing with habitat restoration. I have also edited it slightly to be more concise.

Line 65 As mentioned in the comment below, I would expand a little bit the physical differences of tidal ecosystems to more linear stream ecosystems. Maybe also worth to expand more generally about differences in biology of the beaver (why is it in the tidal marshes, what is it building dams out of, what is it feeding on)

RESPONSE: Line 65 (and this whole paragraph in general) has been moved to the Discussion, following the recommendation of another reviewer. I have also added a paragraph to the Introduction that describes the physical differences between fluvial and tidal systems, including differences in channel network geometry. However, with regard to differences in the biology of beaver between the two systems, nothing is actually known about these differences—there is no literature on this subject. The purpose of this paper is to explore some of these questions of tidal beaver biology. Thus, rather than discussing this in the Introduction, I think it more appropriate to do so in the Discussion.

Line 70 From here on, I got confused. The first sentence is clear, but then I feel the last sentence might be more appropriate and I also do not understand why the small stream are inhospitable.

RESPONSE: I am glad to see that the reviewer liked both sentences. I think they bracket the paragraph well. However, I revised the last part of the last sentence to clarify why small streams are inhospitable (low inflow = high salinity).

Maybe a broader introduction into the dynamics of tidal habitats could help follow the reader here. The sentence from line 71 I would move to the end and start with something like “to do this, we choose the following study regions representing ….”

RESPONSE: I added this recommended detail to the second paragraph of the “Study areas” section of the methods, where I discuss tides and salinity.

Methods

Whatever you decide to do with the results, follow the same logic in the methods.

I think a simple map, or at least a better description where these sites are and how much they are separated would help the reader.

RESPONSE: Maps of this kind was presented in Figs 1-3 (Fig 1 was revised to include the local-scale map in response to another comment) and Fig 9 in the results section (the reviewer liked Fig 9 a lot). It is challenging to keep figures to a minimum and avoid redundancy.

I am not even sure the country has been mentioned.

RESPONSE: A parenthetical statement was added to address this in the 1st line of the “Study Areas” section of the Methods.

I am generally confused by the many localities mentioned and specific histories that I am not sure are important for the paper’s questions.

RESPONSE: I have deleted mention of many of these localities in response to this reviewer’s comment and to reviewer #2’s recommendation that I be more “concise”.

Line 139 Spell out abbreviation for RTK

RESPONSE: Reviewer #3 made the same comment. Changed as suggested.

Results

The result need a lot of restructuring. Currently, this is not publishable, as it is hard to follow and often lost in details. I tried to suggest improvements, because I think the manuscript idea is great!

RESPONSE: In response to all three reviewers, I have edited the text to be more concise, remove unessential details (like the abundance of place names), and to focus on principal points.

I suggest 3-4 Figures, in total one per section.

RESPONSE: I have reduced the number of figures, but not to the extreme suggested by this reviewer.

Whitin the sections:

- Separate your results better. Answer the main questions.

- A lot of the site-specifics has to go to help the reader better follow. Consider to elaborate and describe site specifics in the appendix. The results need to be generalized (at least the text).

RESPONSE: In response to all three reviewers, I have edited the text to be more concise, and I have removed mention of place names as much as possible.

Maybe have two section types. First a section of the dams and transects (things you measured), then a section about naturalist history, eg how the dams are build (things you observed).

RESPONSE: I don’t think this distinction between measurement and observation is very clear or helpful. Measurements are a type of observation.

I think some of these Figure S1-S9 can go into a composite figure in the main manuscript to illustrate the habitat.

RESPONSE: I weighed this suggestion very carefully, because I love figures, but I also understand the need to be concise and focused. The Supporting Information document is a wonderful compromise although not every reader takes advantage of it. I don’t think even figures S1-9 do justice to the variety of tidal environments in which beaver dams can be found; all of the other figures in the Supplemental I

Attachment Submitted filename: Response to Reviewers.docx

# Peer review: Decision Letter 1

**DOI:** 10.1371/journal.pone.0349313.r003

**Authors:** Goulven G Laruelle

14 Apr 2026

PONE-D-25-54787R1

Beaver in tidal habitat: Examples from the Pacific Northwest

PLOS One

Dear Dr. Hood,

Thank you for submitting your manuscript to PLOS ONE. After careful consideration, we feel that it has merit but does not fully meet PLOS ONE’s publication criteria as it currently stands. Therefore, we invite you to submit a revised version of the manuscript that addresses the points raised during the review process.

As you can see, both reviewers were largely satisfied with the corrections you did to the manuscript and convinced it now is close to a state in which it could be published. One of them nevertheless asks for a clarification regarding figure 7 and what the notations NF blind, NF distrib stand for and the other one, while acknowledging that the manuscript is very well written, suggestions that the editing could still be a bit improved, especially but making the research goals clearer.

Not being a native English speaker myself, I feel a little awkward asking one to improved his writing, especially because I also enjoyed reading the manuscript that I do find very well written indeed. However I also agree that some minor editing and attention to keep the flow of the text focused on the research question might help "holding the hand" of the reader along this really nice study.

I thus call this minor revisions because the option "superficial modifications" does not exist in the system. I will be very happy to accept the next version of your manuscript without sending it back for review if you agree to take into consideration these small requests.

Please submit your revised manuscript by May 29 2026 11:59PM. If you will need more time than this to complete your revisions, please reply to this message or contact the journal office at plosone@plos.org. When you’re ready to submit your revision, log on to https://www.editorialmanager.com/pone/ and select the ‘Submissions Needing Revision’ folder to locate your manuscript file.

Please include the following items when submitting your revised manuscript:

- A letter that responds to each point raised by the academic editor and reviewer(s). You should upload this letter as a separate file labeled ‘Response to Reviewers’.
- A marked-up copy of your manuscript that highlights changes made to the original version. You should upload this as a separate file labeled ‘Revised Manuscript with Track Changes’.
- An unmarked version of your revised paper without tracked changes. You should upload this as a separate file labeled ‘Manuscript’.

If you would like to make changes to your financial disclosure, please include your updated statement in your cover letter. Guidelines for resubmitting your figure files are available below the reviewer comments at the end of this letter.

If applicable, we recommend that you deposit your laboratory protocols in protocols.io to enhance the reproducibility of your results. Protocols.io assigns your protocol its own identifier (DOI) so that it can be cited independently in the future. For instructions see: https://journals.plos.org/plosone/s/submission-guidelines#loc-laboratory-protocols. Additionally, PLOS ONE offers an option for publishing peer-reviewed Lab Protocol articles, which describe protocols hosted on protocols.io. Read more information on sharing protocols at https://plos.org/protocols?utm_medium=editorial-email&utm_source=authorletters&utm_campaign=protocols.

As the corresponding author, your ORCID iD is verified in the submission system and will appear in the published article. PLOS supports the use of ORCID, and we encourage all coauthors to register for an ORCID iD and use it as well. Please encourage your coauthors to verify their ORCID iD within the submission system before final acceptance, as unverified ORCID iDs will not appear in the published article. *Only*   the individual author can complete the verification step; PLOS staff *cannot*   verify ORCID iDs on behalf of authors.

We look forward to receiving your revised manuscript.

Kind regards,

Goulven G Laruelle

Academic Editor

PLOS One

Journal Requirements:

If the reviewer comments include a recommendation to cite specific previously published works, please review and evaluate these publications to determine whether they are relevant and should be cited. There is no requirement to cite these works unless the editor has indicated otherwise.

Please review your reference list to ensure that it is complete and correct. If you have cited papers that have been retracted, please include the rationale for doing so in the manuscript text, or remove these references and replace them with relevant current references. Any changes to the reference list should be mentioned in the rebuttal letter that accompanies your revised manuscript. If you need to cite a retracted article, indicate the article’s retracted status in the References list and also include a citation and full reference for the retraction notice.

[Note: HTML markup is below. Please do not edit.]

Reviewers’ comments:

Reviewer’s Responses to Questions

**Comments to the Author**

1. If the authors have adequately addressed your comments raised in a previous round of review and you feel that this manuscript is now acceptable for publication, you may indicate that here to bypass the “Comments to the Author” section, enter your conflict of interest statement in the “Confidential to Editor” section, and submit your "Accept" recommendation.

Reviewer #1: All comments have been addressed

Reviewer #2: (No Response)

**********

2. Is the manuscript technically sound, and do the data support the conclusions?

The manuscript must describe a technically sound piece of scientific research with data that supports the conclusions. Experiments must have been conducted rigorously, with appropriate controls, replication, and sample sizes. The conclusions must be drawn appropriately based on the data presented.

Reviewer #1: Yes

Reviewer #2: Yes

**********

3. Has the statistical analysis been performed appropriately and rigorously?

Reviewer #1: Yes

Reviewer #2: Yes

**********

4. Have the authors made all data underlying the findings in their manuscript fully available?

The PLOS Data policy requires authors to make all data underlying the findings described in their manuscript fully available without restriction, with rare exception (please refer to the Data Availability Statement in the manuscript PDF file). The data should be provided as part of the manuscript or its supporting information, or deposited to a public repository. For example, in addition to summary statistics, the data points behind means, medians and variance measures should be available. If there are restrictions on publicly sharing data—e.g. participant privacy or use of data from a third party—those must be specified.

Reviewer #1: Yes

Reviewer #2: Yes

**********

5. Is the manuscript presented in an intelligible fashion and written in standard English?

PLOS ONE does not copyedit accepted manuscripts, so the language in submitted articles must be clear, correct, and unambiguous. Any typographical or grammatical errors should be corrected at revision, so please note any specific errors here.

Reviewer #1: Yes

Reviewer #2: Yes

**********

6. Review Comments to the Author

Please use the space provided to explain your answers to the questions above. You may also include additional comments for the author, including concerns about dual publication, research ethics, or publication ethics. (Please upload your review as an attachment if it exceeds 20,000 characters)

Reviewer #1: Thanks to the authors for a good revision, I think the manuscript has improved significantly, and I think it makes a valuable contribution to beaver and tidal science.

It was a lot easier to read now, and I appreciate the authors trying to better explain the different terms.

one small comment:

Figure 7: It is still not easy to understand what is NF blind, NF distrib. The abbreviation NF is not explained elsewhere in the manuscript as far as I saw. I assume it is North Fork. Maybe that can be put in the figure legend.

Reviewer #2: This is an epic piece of work, well written and informative and novel. I just find it could still benefit with robust editing as it is very long and detailed that I’m not sure improves the readability of the text eg vegetation descriptions, I’m getting a bit lost with the relevance to the study, which i still feel could have clearer research goals

**********

7. PLOS authors have the option to publish the peer review history of their article (what does this mean?). If published, this will include your full peer review and any attached files.

If you choose “no”, your identity will remain anonymous but your review may still be made public.

**Do you want your identity to be public for this peer review?**   For information about this choice, including consent withdrawal, please see our Privacy Policy.

Reviewer #1: No

Reviewer #2: No

**********

[NOTE: If reviewer comments were submitted as an attachment file, they will be attached to this email and accessible via the submission site. Please log into your account, locate the manuscript record, and check for the action link "View Attachments". If this link does not appear, there are no attachment files.]

To ensure your figures meet our technical requirements, please review our figure guidelines: https://journals.plos.org/plosone/s/figures

You may also use PLOS’s free figure tool, NAAS, to help you prepare publication quality figures: https://journals.plos.org/plosone/s/figures#loc-tools-for-figure-preparation.

NAAS will assess whether your figures meet our technical requirements by comparing each figure against our figure specifications.

# Author comment: Author response to Decision Letter 2

**DOI:** 10.1371/journal.pone.0349313.r004

22 Apr 2026

Responses to Reviewers and Editor (part 2) 22 April 2026

As you can see, both reviewers were largely satisfied with the corrections you did to the manuscript and convinced it now is close to a state in which it could be published. One of them nevertheless asks for a

[Reviewer] clarification regarding figure 7 and what the notations NF blind, NF distrib stand for

I made two changes to address this recommendation: [1] I added a parenthetical “(NF)” after the text “North Fork” in the manuscript figure legend (see track changes) to show that “North Fork” was being abbreviated to “NF”, and [2] I edited the legend box in the top figure changing “blind” to “tidal” to be consistent with the rest of the text, as the term “blind channel” was changed to merely “tidal” channel in response to previous recommendations by reviewers—I simply missed making the change in this figure; I also fully spelled out “distributary” to replace to previous abbreviation “distrib”.

and the other one, while acknowledging that the manuscript is very well written, [Reviewer] suggestion that the editing could still be a bit improved, especially by making the research goals clearer.

[Editor] I also agree that some minor editing and attention to keep the flow of the text focused on the research question might help "holding the hand" of the reader along this really nice study.

I have addressed these concerns by making edits in two locations in the manuscript. The first section of new text occurs from line 84 to 91 and consists of the following new text (underlined and blue) with original text in black and mostly removed. I believe this first section more explicitly details the goals of the paper, as requested.

“The goal of this focus is to explore spatial patterns in dam and lodge locations (a) to provide insight into the abundance of beaver structures and their distribution relative to estuarine habitats, e.g., characterized by vegetation communities and channel networks; (b) to allow comparison with fluvial beaver, e.g., are there significant differences in dam or lodge density that might suggest differences in habitat quality or beaver behavior; (c) to understand the function of dams for beaver in tidal environments and how that compares with fluvial dams; and (d) to contribute guidance for tidal wetland restoration design that accommodates beaver and the ecosystem functions they may provide. infer the role of beaver dams in tidal systems, from the perspective of beaver, and contrast it with their role in fluvial systems. An additional contrast is how beaver choose lodge locations in a two-dimensional estuarine landscape compared to a mostly one-dimensional riverine landscape.”

In a second location, from line 367 to 374, I added a paragraph (see text below) that describes a hypothesis that provides context for the results described in subsequent paragraphs and in Fig. 7. In three locations in the results, I also added phrases that indicated consistency or inconsistency with the hypothesis.

“It is reasonable to think that channel size could affect beaver dam construction. If we have a simplistic hypothesis that assumes tidal dams are generally relatively uniform in design (with some variation around a mean), then each dam would have a similar height and water head, so that a nearly constant number of dams would be found in tidal channels regardless of channel size (length), because the difference between low tide channel bottom elevations and MHHW is similar for almost all channels (except small tributaries to tidal channels). If true, then short channels should have steeper gradient and closely spaced dams while long tidal channels should have lower gradient and more widely spaced dams.”

Attachment Submitted filename: Response to Reviewers 22Apr2026.docx

# Editor report: Decision Letter 2

**DOI:** 10.1371/journal.pone.0349313.r005

**Authors:** Goulven G Laruelle

29 Apr 2026

Beaver in tidal habitat: Examples from the Pacific Northwest

PONE-D-25-54787R2

Dear Dr. Hood,

We’re pleased to inform you that your manuscript has been judged scientifically suitable for publication and will be formally accepted for publication once it meets all outstanding technical requirements.

Within one week, you’ll receive an e-mail detailing the required amendments. When these have been addressed, you’ll receive a formal acceptance letter and your manuscript will be scheduled for publication.

An invoice will be generated when your article is formally accepted. Please note, if your institution has a publishing partnership with PLOS and your article meets the relevant criteria, all or part of your publication costs will be covered. Please make sure your user information is up-to-date by logging into Editorial Manager at Editorial Manager® and clicking the ‘Update My Information’ link at the top of the page. For questions related to billing, please contact billing support.

If your institution or institutions have a press office, please notify them about your upcoming paper to help maximize its impact. If they’ll be preparing press materials, please inform our press team as soon as possible -- no later than 48 hours after receiving the formal acceptance. Your manuscript will remain under strict press embargo until 2 pm Eastern Time on the date of publication. For more information, please contact onepress@plos.org.

Kind regards,

Goulven G Laruelle

Academic Editor

PLOS One

Additional Editor Comments (optional):

As I said in my previous message, the final round of revisions was very minor and I appreciate that you took the time to address them carefully. I am now very happy to accept your manuscript as it now stands and forward it to the production team.

Reviewers’ comments:

# Editor report: Acceptance letter

**DOI:** 10.1371/journal.pone.0349313.r006

**Authors:** Goulven G Laruelle

PONE-D-25-54787R2

PLOS One

Dear Dr. Hood,

I’m pleased to inform you that your manuscript has been deemed suitable for publication in PLOS One. Congratulations! Your manuscript is now being handed over to our production team.

At this stage, our production department will prepare your paper for publication. This includes ensuring the following:

* All references, tables, and figures are properly cited

* All relevant supporting information is included in the manuscript submission,

* There are no issues that prevent the paper from being properly typeset

You will receive further instructions from the production team, including instructions on how to review your proof when it is ready. Please keep in mind that we are working through a large volume of accepted articles, so please give us a few days to review your paper and let you know the next and final steps.

Lastly, if your institution or institutions have a press office, please let them know about your upcoming paper now to help maximize its impact. If they’ll be preparing press materials, please inform our press team within the next 48 hours. Your manuscript will remain under strict press embargo until 2 pm Eastern Time on the date of publication. For more information, please contact onepress@plos.org.

You will receive an invoice from PLOS for your publication fee after your manuscript has reached the completed accept phase. If you receive an email requesting payment before acceptance or for any other service, this may be a phishing scheme. Learn how to identify phishing emails and protect your accounts at https://explore.plos.org/phishing.

If we can help with anything else, please email us at customercare@plos.org.

Thank you for submitting your work to PLOS ONE and supporting open access.

Kind regards,

PLOS ONE Editorial Office Staff

on behalf of

Dr. Goulven G Laruelle

Academic Editor

PLOS One
